# Induction by *Bradyrhizobium diazoefficiens* of Different Pathways for Growth in D-mannitol or L-arabinose Leading to Pronounced Differences in CO_2_ Fixation, O_2_ Consumption, and Lateral-Flagellum Production

**DOI:** 10.3389/fmicb.2018.01189

**Published:** 2018-06-05

**Authors:** Carolina Cogo, Julieta Pérez-Giménez, Chandrasekar B. Rajeswari, María F. Luna, Aníbal R. Lodeiro

**Affiliations:** ^1^Instituto de Biotecnología y Biología Molecular, Facultad de Ciencias Exactas-UNLP y CCT La Plata-CONICET, La Plata, Argentina; ^2^Departamento de Ciencias Básicas, Facultad de Ingeniería-UNLP, La Plata, Argentina; ^3^Centro de Investigación y Desarrollo en Fermentaciones Industriales, Facultad de Ciencias Exactas-UNLP y CCT La Plata-CONICET, Comisión de Investigaciones Científicas de la Provincia de Buenos Aires, La Plata, Argentina

**Keywords:** *Bradyrhizobium*, CBB pathway, PP pathway, L-KDA pathway, flagella, O_2_ consumption

## Abstract

*Bradyrhizobium diazoefficiens*, a soybean N_2_-fixing symbiont, constitutes the basic input in one of the most prominent inoculant industries worldwide. This bacterium may be cultured with D-mannitol or L-arabinose as carbon-plus-energy source (C-source) with similar specific growth rates, but with higher biomass production with D-mannitol. To better understand the bacterium’s carbon metabolism, we analyzed, by liquid chromatography and tandem mass spectrometry (MS), the whole set of proteins obtained from cells grown on each C-source. Among 3,334 proteins identified, 266 were overproduced in D-mannitol and 237 in L-arabinose, but among these, only 22% from D-mannitol cultures and 35% from L-arabinose cultures were annotated with well defined functions. In the D-mannitol-differential pool we found 19 enzymes of the pentose-phosphate and Calvin–Benson–Bassham pathways and accordingly observed increased extracellular-polysaccharide production by D-mannitol grown bacteria in a CO_2_-enriched atmosphere. Moreover, poly-3-hydroxybutyrate biosynthesis was increased, suggesting a surplus of reducing power. In contrast, the L-arabinose-differential pool contained 11 enzymes of the L-2-keto-3-deoxyarabonate pathway, 4 enzymes for the synthesis of nicotinamide-adenine dinucleotide from aspartate, with those cultures having a threefold higher O_2_-consumption rate than the D-mannitol cultures. The stoichiometric balances deduced from the modeled pathways, however, resulted in similar O_2_ consumptions and ATP productions per C-mole of substrate. These results suggested higher maintenance-energy demands in L-arabinose, which energy may be used partly for flagella-driven motility. Since *B. diazoefficiens* produces the lateral-flagella system in only L-arabinose, we calculated the O_2_-consumption rates of a *lafR*::Km mutant devoid of lateral flagella cultured in L-arabinose or D-mannitol. Contrary to that of the wild-type, the O_2_-consumption rate of this mutant was similar on both C-sources, and accordingly outcompeted the wild-type in coculture, suggesting that the lateral flagella behaved as parasitic structures under these conditions. Proteomic data are available via ProteomeXchange with identifier PXD008263.

## Introduction

*Bradyrhizobium diazoefficiens*—one of the principal N_2_-fixing symbionts of soybean—is cultured industrially to obtain legume inoculants worldwide. As other rhizobia, *B. diazoefficiens* is able to use a diversity of carbon sources including carbohydrates, polyols, organic acids, and aminoacids (Delamuta et al., 2013). These nutrients cause differences in growth yield and infectivity, and therefore must be carefully selected for culture media formulation by the inoculant industry to obtain symbiotically competent rhizobia along with a high biomass yield, at the lowest possible cost. In addition, each carbon source may produce different physiologic effects in the bacteria, which might have an impact on the inoculant’s performance in the field.

In *Bradyrhizobium* laboratory research, the two most widely used carbon-plus-energy sources (C-sources) for *B. diazoefficiens* are D-mannitol (Mtl) and L-arabinose (Ara) (e.g., Mathis et al., 1986; Sadowsky et al., 1987; Brunel et al., 1988; Hauser et al., 2007). Early studies demonstrated that the O_2_-consumption rate varied widely with one or the other of those carbon sources (Thorne and Burris, 1940); but, to the best of our knowledge, such studies were never confirmed with more modern technologies involving well characterized type strains. In addition, the catabolic pathways of those molecules seemed substantially different, although a certain controversy exists on the pathway for Mtl consumption (Pedrosa and Zancan, 1974; Mulongoy and Elkan, 1977; Stowers, 1985; Sosa-Saavedra et al., 2001; Fuhrer et al., 2005; Polcyn and Podeszwa, 2009; Koch et al., 2014). The differences in the metabolism of those two carbon sources lead to differences in the production of EPSs-that include the exo- and capsular-polysaccharides-as well as to soybean lectin binding (Tully, 1985; Karr et al., 2000), which have a positive influence on adhesion to plant roots, rhizosphere colonization, infectivity, and competitiveness for nodulation (López-García et al., 2001; Quelas et al., 2006, 2010; Pérez-Giménez et al., 2009).

More recently, it was reported that *B. diazoefficiens* possesses two independent flagellar systems (Kanbe et al., 2007; Althabegoiti et al., 2008, 2011; Quelas et al., 2016a). Normally, bacteria with dual flagellar systems express one for swimming in liquid medium and the other for swarming over wet surfaces (Partridge and Harshey, 2013); but *B. diazoefficiens*, together with *Shewanella putrefaciens*, are the only bacteria known to produce both functional systems simultaneously in liquid medium (Bubendorfer et al., 2014; Quelas et al., 2016a). In the example of *B. diazoefficiens*, the primary (subpolar) flagellar system is expressed constitutively, while the secondary (lateral) system is fully expressed with Ara but is undetectable with Mtl as the carbon source (Covelli et al., 2013; Mongiardini et al., 2017). Once expressed, both flagellar systems are active in liquid medium and integrate their functions in an emergent swimming performance that cannot be explained by the additive effects of each flagellar system (Quelas et al., 2016a). The numerous lateral flagella, however—which probably consume more energy for their synthesis and functioning than the single subpolar flagellum—are seemingly unable to respond to known chemotactic stimuli (Quelas et al., 2016a) and are unnecessary for the competitiveness of the bacterium for rhizosphere colonization and soybean nodulation (Althabegoiti et al., 2011), thus raising questions about the adaptive value of those flagella in establishing the bacteria in such symbiotic—and otherwise advantageous—niches.

Thus, the use of one or the other carbon and energy source may have profound impacts on bacterial morphology and physiology, beyond the differences in the catabolic-pathway biochemistry *per se*. Nevertheless, up to the present, no clear physiologic linkages have been established between these catabolic pathways, the O_2_-consumption rate, and the functionality of different extracellular products and/or structures such as the EPS and the flagella, both of which features mediate key aspects of soybean-root infection and subsequent symbiotic N_2_-fixation. To better characterize all these relationships, in the work reported here, we analyzed the differential protein-expression profiles of *B. diazoefficiens* USDA 110 in medium containing Mtl or Ara by liquid chromatography coupled in tandem to MS employing a high-resolution and high–mass-accuracy Orbitrap equipment. On the basis of this information, and using complementary bioinformatics approaches, we identified enzymes that might be involved in Mtl and Ara degradation, modeled the Mtl- and Ara-catabolic pathways, and compared the energetic efficiencies between those two degradative schemes through experimental measurements of the O_2_-consumption rates on either Mtl or Ara medium in both the wild-type *B diazoefficiens* and a mutant devoid of lateral flagella.

## Materials and Methods

### Strains and Culture Conditions

The wild-type *B. diazoefficiens* strain employed in this work is a spontaneous Sm resistant derivative from the type-strain USDA 110. The *lafR*::Km mutant strain is a USDA 110 insertional mutant in which the Km resistance cassette from the plasmid pUC4K was inserted into the coding region of *lafR* (Mongiardini et al., 2017). We grew the bacteria for inoculum in liquid HM salts–yeast-extract medium (HMY) supplemented with either 0.5% (w/v) Mtl or 0.5% (w/v) Ara (Mongiardini et al., 2017) as the C sources (HMY-Mtl or HMY-Ara cultures, respectively), taking into account that the C-mole of each substrate is very similar (30.3 and 30.0 g.C-mole^-1^ for Mtl and Ara, respectively). These cultures were grown at 30°C with rotary shaking at 180 rpm. We carried out the batch cultures of the wild-type and the mutant strain in an LH-210 Bioreactor (Inceltech, Toulouse, France) with a working volume of 4 l in HMY-Mtl or HMY-Ara at 30°C and 250 rpm with air flushed at 25–30 l.h^-1^.

### Protein Isolation

We studied the differential proteomes in *B. diazoefficiens* USDA 110 with protein samples obtained from bacteria in the exponential phase of growth (OD_500__nm_ = 1.0) in HMY-Mtl or HMY-Ara. We obtained the rhizobia samples from three independent cultures per condition by centrifugation at 7,150 × *g*, followed by two washes of the pellets with low-salt washing buffer (3 mM KCl, 1.5 mM KH_2_PO_4_, 68 mM NaCl, 9 mM NaH_2_PO_4_; Pérez-Giménez et al., 2012) and then resuspended the pellets in 10 mM Tris-HCl pH 8.5 up to an OD_500__nm_ = 10, adding 1 μl of phenyl methyl sulfonyl fluoride in Tris-HCl per ml. We then lysed the cells by ultrasound with a Sonifier 150 (Branson) sonicator employing six 3-min pulses at 38% power in an ice bath and centrifuged the lysate at 13,520 × *g* for 30 min to remove the unbroken cells and cell debris. We next centrifuged the resulting supernatant at 66,226 × *g* for 2 h to pellet the microsomal fraction, though also retaining that supernatant. To collect the membrane-enriched–protein fraction, we resuspended the pellets in 250 μl of rehydration solution (7 M urea, 2 M thiourea, 10% [v/v] isopropanol, 2% [v/v] triton X-100). In parallel, to each of those supernatants, we added 4 volumes of acetone for at least 12 h at -20°C to precipitate the cytoplasm-enriched proteins, before a final centrifugation at 13,520 × *g* for 20 min and resuspension in 400 μl of rehydration solution containing 7 M urea and 2 M thiourea. We quantified the proteins according to the method of Bradford (1976) and assessed the quality of the samples by one- and two-dimensional sodium-dodecyl-sulfate–polyacrylamide-gel electrophoresis (SDS-PAGE; Laemmli, 1970; Supplementary Figure S1).

### Protein Separation and Analysis

We reduced the protein samples in rehydration solution with dithiothreitol in 50 mM ammonium bicarbonate at a final concentration of 10 mM (45 min, 56°C) followed by alkylation with iodoacetamide at a final concentration of 20 mM in the same solution (40 min, room temperature, in the dark). We precipitated the protein solution with 0.2 volumes of trichloroacetic acid at -20°C for at least 2 h followed by centrifugation at 14,000 × *g* for 10 min (4°C) and then washed the pellet twice with cold acetone before a drying at room temperature. In the CEQUIBIEM facility (Center of Chemical and Biochemical Studies by Mass Spectrometry, Universidad de Buenos Aires, Argentina), we resuspended the proteins to a final concentration of 2 mg ml^-1^ in 50 mM ammonium bicarbonate, pH 8, followed by digestion with 15 μg ml^-1^ trypsin (Promega V5111). We then purified and desalted the peptides with ZipTip C18 columns (Millipore) before performing a nano-HPLC separation coupled to MS on an EASY-nLC 1000 instrument (Proxeon, Thermo Scientific) using an Easy-Spray Column PepMap RSLC (P/N ES801) suitable for separating protein complexes with a high degree of resolution by reverse-phase chromatography (C18, 2 μm, 100 Å, 50 μm × 150 mm). The flow rate used for the nano column was 300 nl min^-1^ with the solvents A (0.1% [v/v] formic acid in water) and B (0.1% [v/v] formic acid in acetonitrile), ranging from 93% A: 7% B (5 min) to 65% A: 35% B (120 min), and a 2-μl injection volume. The MS equipment contained a high-collision dissociation cell for fragmentation and an Orbitrap analyzer (Thermo Scientific, Q-Exactive).

For data acquisition, we used the XCalibur 3.0.63 (Thermo Scientific) software with an equipment configuration that performed a simultaneous peptide identification during the chromatographic separation. The equipment made a full MS (resolution: 70,000) and a tandem MS/MS with the 15 best signal-to-noise–ratio peaks in each cycle (resolution: 17,500), with a dynamic exclusion range to avoid fragmentation of the same peak more than once during the same elution profile of the chromatogram. This method facilitated the highest number of cycles per unit time. A voltage of 3.5 kV was used for electro-spray ionization (Thermo Scientific, EASY-SPRAY).

We calculated the areas under the peaks by means of the algorithms used by the Proteome Discoverer software (Thermo Scientific, 1.4 version). The program was designed to search within the *B. diazoefficiens* USDA 110 data base UP000002526 (Uniprot) after the execution of a virtual trypsin digestion. We performed Proteome Discoverer searches with a precursor-ion tolerance of 10 ppm and a fragment-ion-mass tolerance of 0.05 Da specifying the oxidation of methionine as the dynamic modification and carbamidomethylation as the static modification. We set the peptide level of confidence at “high” (at a 1% false-discovery rate) and finally processed the data of the areas obtained with the Perseus software (Max Planck Institute of Biochemistry, 1.5.5.3 version, freely available), which informatics executes an in-depth statistical analysis. We replaced the missing values with 0 when proteins were present in both instances (i.e., Mtl and Ara) and with 1 when proteins were absent in one (i.e., either Mtl or Ara). Through this algorithm, we were able to identify 3,334 proteins, which species are detailed in Supplementary Table S1.

### Bioinformatics Analyses

We searched the protein lists obtained differentially with Mtl or Ara with the Rhizobase^1^, Uniprot^2^, and KEGG Pathways^3^ databases to look for each protein’s possible function. We then classified the proteins according to the pathways in which those species might take part and used the proteins’ gene tags from Rhizobase to locate them in the KEGG-Pathways maps. Then, we focused on those pathways for which groups of enzymes-instead of single enzymes-were differentially found either in HMY-Mtl or HMY-Ara-grown cultures, to raise hypotheses about the differential metabolic fluxes in each carbon source. Finally, to validate these hypotheses, we continued evaluating whether the other enzymes of each candidate pathway were actually detected by our proteomic experiments, even if these latter enzymes were not included in the differential pools (Supplementary Table S1). With this information, we modeled the stoichiometry of the pathways, considering only those proteins that we detected experimentally at least once in our biological samples (Supplementary Tables S6–S12).

To look for possible operon organization, we searched those proteins that had successive gene tags (Rhizobase), in either differential pool of proteins, with the MicrobesOnline^4^ to corroborate whether those proteins constituted parts of operons in the *B. diazoefficiens* USDA 110 genome (Supplementary Tables S2, S3).

### Poly-3-Hydroxybutyrate Extraction and Determination

We extracted the PHB from whole cells by two successive centrifugations at 12,000 × *g* during 40 min each. Then, we homogenized pellets with sodium hypochlorite overnight at room temperature, precipitated with 1:1 alcohol-acetone, and resuspended in chloroform. Next, we quantified the PHB by a previously described spectrophotometric method (Law and Slepecky, 1961).

### Analysis of Growth Parameters

We determined the O_2_ and CO_2_ concentrations in the supplied and emitted gasses using a paramagnetic oxygen analyzer (Servomex 1100 A; Norwood, MA, United States) and an infrared CO_2_ analyzer (Horiba PIR 2000; Japan) and measured the gas-flow rates with a bubble flow meter. We determined biomass dry weight as described (Herbert et al., 1971) and measured the substrate (Mtl or Ara) concentration in culture supernatants (from either the used HMY-Mtl or the HMY-Ara) by HPLC. To this end, we filtered the samples through 0.22-μm nitrocellulose membranes and manually injected 10 μl of each sample with a rheodyne 7725i injector into a Carbopac PA1 column maintained at 35 °C. The chromatography involved a mobile phase of 0.1 M NaOH and a flow rate of 1.0 ml.min^-1^, with peak heights measured by a Waters 2465 electrochemical detector and the substrate concentrations quantified by means of the software Empower Pro, 2002 Waters.

We calculated the *μ*_max_ from the linear regression of the Napierian logarithmic values (*ln*) of biomass dry weight during the exponential growth phase and determined the *qO*_2max_ plus the CO_2_ production by a mass-balance method (Cooney et al., 1977). We calculated the biomass yield and specific rates of substrate consumption as described (Roels, 1983).

### Cocultivation of Different *B. diazoefficiens* Strains to Determine Relative Growth-Competitive Abilities

We grew the wild-type and the *lafR*::Km mutant from starter cultures as described above, and when the bacteria reached the exponential growth phase, diluted each resulting suspension to (1.5 ± 0.2).10^9^ colony-forming units (CFUs).ml^-1^ either in HMY-Mtl or HMY-Ara. We then inoculated 100 μl of each of these diluted suspensions into 10 ml of each medium followed by cultivation at 30°C with rotary shaking at 180 rpm. In parallel, we mixed 50 μl of each strain together and inoculated the resulting 100 μl of the 1:1 admixture into 10 ml of the corresponding culture medium, in order to ensure that at time zero of the experiment, all cultures started with the same total cell densities. Then, we continued cultivation under the same conditions until the cultures reached the stationary phase, which growth took 4.0 days in HMY-Mtl or 2.5 days in HMY-Ara. At these times we removed two 100-μl aliquots from each culture, used one for serial dilutions and CFU counts, and reinoculated the other into 10 ml fresh medium for further cultivation under the same conditions to strengthen the competition between the two strains. Once these subcultures reached the stationary phase, we repeated the process once more to obtain a third subculture, counting CFUs from each subculture after the appropriate dilution in yeast-extract mannitol agar (YMA) supplemented with either Sm (400 μg.ml^-1^) for wild-type selection or Km (150 μg.ml^-1^) for mutant selection in at least five replicas. The whole experiment was repeated in its entirety.

### Statistical Procedures

For proteomic comparisons, we employed duplicate or triplicate independent cultures (biologic replicates) from each culture medium and each subcellular fraction. With these replicates we constructed volcano plots to compare the samples using the logarithmic values of the ratio of the areas under the peaks along with the statistical significance of those values assessed by the Student-*t*-test (not shown). Hence, for each Mtl/Ara pair we plotted -log_10_ Student-*t*-test *p*-value on the *y* axis *versus* log_2_ fold change (Mtl area/Ara area) on the *x* axis. We considered differentially expressed proteins to be those with fold-changes greater than 2 (less than -1 or greater than 1 on the *x* axis of the graph) and with *p*-values lower than 0.05 (of values greater than 1.3 on the *y* axis of the graph). We also employed the same *p*-value threshold to determine the proteins present under only one condition (either growth in Mtl or Ara); then, for each C-source, combined the differentially expressed proteins—i.e., those with significantly greater fold-changes on that C-source and those exclusively present under that growth condition—in a single group to obtain the two lists comprising the Mtl-differential pool (MtlDP, Supplementary Table S2), and the Ara- differential pool (AraDP, Supplementary Table S3). Comparisons between these differential pools were carried out only for the groups of enzymes that belonged to the same pathway or part of a pathway, and that demonstrated consistent patterns in their variation. For all other experiments we used duplicate independent biologic replicas with at least three technical replicas each. The values reported are the means ± SD.

### Data Submission

The mass spectrometry proteomics data have been deposited to the ProteomeXchange Consortium via the PRIDE (Vizcaíno et al., 2016) partner repository with the dataset identifier PXD008263 and 10.6019/PXD008263.

## Results

### Differential-Protein Profiles

We obtained the total proteins from the cytoplasm- or membrane-enriched fractions of *B. diazoefficiens* USDA 110 grown in triplicate independent batch cultures in HMY medium, either with 0.5% (w/v) Mtl or 0.5% (w/v) Ara as the C-source. The bacteria were harvested in the exponential-growth phase at an OD_500__nm_ = 1.0. Upon assessing the quality of the samples by SDS-PAGE, we also corroborated that lateral flagellins were produced in only the HMY-Ara (Supplementary Figure S1). Next, the entire set of proteins obtained was analyzed by nano-HPLC coupled in tandem to mass spectrometry (nLC-MS/MS). Through an in-depth analysis of the proteomics data, we identified the 3,334 proteins listed in Supplementary Table S1, those representing 40% of the 8,317 predicted proteins of the whole *B. diazoefficiens* proteome (Kaneko et al., 2002), despite the differences in yield among the samples (**Table 1**). The membrane-enriched fractions obtained in HMY-Mtl and HMY-Ara contained 43 and 38% of the total proteins respectively (**Table 1**), which agrees with the 40% of proteins classified as cell wall (GO: 0005618), cell envelope (GO: 030313), and cell membrane (GO: 0044425) in the cell part fraction (GO: 0044464) in Microbes OnLine (consulted on March 28, 2018)^5^. We then looked for possible differences among the proteomes from HMY-Mtl or HMY-Ara cultures, including differential protein abundances along with the presence or absence of proteins in a given sample and averaged the results from at least two biologic replicas per condition and subcellular fraction. In this way, we identified 266 differential proteins pertaining to the HMY-Mtl cultures (Supplementary Table S2) and 237 to the HMY-Ara cultures (Supplementary Table S3)—that in the following will be referred to as the Mtl-differential pool (MtlDP) and the Ara-differential pool (AraDP), respectively. Although the proteins belonging to these pools were obtained from the cytoplasm- or membrane-enriched fractions, 115 proteins (43%) and 91 proteins (38%) appeared in both fractions in MtlDP and AraDP respectively. In addition, about one-third of the proteins that were observed as differentially expressed between Ara and Mtl in one fraction were not significantly so in the other, in at least one sample per fraction (Supplementary Tables S2, S3).

**Table 1:** Summary of the proteins identified by nLC-MS/MS with an Orbitrap instrument in three biological replicas (samples) of *B. diazoefficiens* growing in the indicated liquid media.

	Number of different proteins detected in the fractions			
	HMY-Mtl		HMY-Ara	
Sample #	Cytoplasm-enriched	Membrane-enriched	Cytoplasm-enriched	Membrane-enriched
1	407	597	1390	31
2	1948	1531	1797	1583
3	1966	1729	1784	1854
				
Average	1440	1286	1657	1156
				
Total	3008		3018
% Superposition cytoplasm/membrane	37.7		35.3

We looked for the possible functions of these proteins in the Rhizobase, the Uniprot, and the KEGG databases and found that only 21.8% of the proteins in the MtlDP and 35.0% in the AraDP were annotated with well defined functions (Supplementary Table S4). This subset of proteins is listed in Supplementary Table S5, there classified by function according to the gene-ontology (GO)–biological-processes assignment in Uniprot. On the basis of this subset, we intended to evaluate the differential cellular status in the HMY-Mtl, and HMY-Ara cultures; focussing on the enzymes clearly identified as participating in, at once, Mtl and Ara catabolism, PHB synthesis, and biotin metabolism along with those proteins involved in cell motility and chemotaxis.

In our attempt to deduce the metabolic pathways operating with one or the other C-source, we considered only sets of differentially expressed enzymes acting in the same pathway of *B. diazoefficiens* according to the KEGG database, since metabolic fluxes are seldom influenced by changes in a single enzyme of one pathway (Fell, 2005). By this means, we were able to identify substantial differences between the two metabolic pathways that may be inferred from the protein profiles obtained (**Table 2**).

**Table 2:** Selected proteins from the MtlDP and the AraDP that seemed to play roles in the metabolic pathways and in motility.

Gene tag (Rhizobase)	Protein ID (Uniprot)	Gene name	Description	Fold-change (log_2_ Mtl area/Ara area)	
				Cytoplasm	Membrane
			*Mtl catabolic pathway*		
blr0337	Q89XH7		Putative carbon monoxide dehydrogenase medium chain.	1.1132	NS
bll1419	Q89UJ7	*metF*	Methylenetetrahydrofolate reductase.	In only Mtl	NS
bll1521	Q89U96		Putative fructose-1.6-bisphosphate aldolase protein.	1.9439	NS
blr2168	Q89T82		Putative transketolase alpha subunit protein.	In only Mtl	-
blr2581	Q8GKS1	*fbp*	Fructose-1.6-bisphosphatase class 1.	In only Mtl	-
blr2582	H7C6I1	*cbbP*	Phosphoribulokinase.	In only Mtl	-
blr2583	H7C6U6	*cbbT*	Transketolase.	In only Mtl	In only Mtl
blr2584	H7C7W4	*cbbA*	Fructose-bisphosphate aldolase.	In only Mtl	In only Mtl
blr2585	Q9ZI34	*cbbL*	Ribulose bisphosphate carboxylase large chain.	In only Mtl	In only Mtl
blr2586	Q9ZI33	*cbbS*	Ribulose bisphosphate carboxylase small chain.	In only Mtl	In only Mtl
blr2588	Q89S24	*cbbE*	Ribulose-phosphate 3-epimerase.	In only Mtl	-
blr2815	Q89RF7		Putative transketolase family protein. Pyruvate dehydrogenase E1 component.	In only Mtl	In only Mtl
blr3224	Q89QA6		ABC transporter ATP-binding protein. Putative sorbitol/mannitol transport system.	In only Mtl	NS
blr3225	Q89QA5		Oxidoreductase. Similar to putative Glucose/ribitol oxidoreductase.	5.0927	In only Mtl
blr3226	Q89QA4		Ribitol kinase	3.9234	-
blr3227	Q89QA3		Putative fructokinase.	In only Mtl	-
bll3754	Q89NT0	*gph*	Phosphoglycolate phosphatase.	In only Mtl	NS
bll6549	Q89FZ9		D-3-phosphoglycerate:NAD^+^ oxidoreductase	In only Mtl	-
blr7063	Q89EL0		Probable 2-ketogluconate reductase.	2.5998	-
			*Ara catabolic pathway*		
bll1188	Q89V68	*atpB*	ATP synthase subunit a.	-	In only Ara
blr2316	Q89ST4		Probable NADH-ubiquinone oxidoreductase chain F.	In only Ara	-
blr2929	Q89R43		Hydroxypyruvate isomerase.	NS	In only Ara
blr2974	Q89QZ8		Dehydratase. Similar to a putative D-galactarate dehydratase.	-1.2323	-
bll3156	Q89QH2	*frc*	Formyl-CoA:oxalate CoA-transferase.	-2.1294	In only Ara
bll3157	Q89QH1	*oxc*	Oxalyl-CoA carboxy-lyase.	-2.3829	NS
blr3166	Q89QG2	*gcl*	Glyoxylate carboligase.	In only Ara	In only Ara
blr3167	Q89QG1	*hyi*	Hydroxypyruvate isomerase.	In only Ara	In only Ara
blr3168	Q89QG0		Tartronate semialdehyde reductase.	In only Ara	In only Ara
blr3205	Q89QC3		Dehydrogenase. Similar to Galactose 1-dehydrogenase.	-	-3.3450
blr3207	Q89QC1		L-arabinonolactonase.	-4.7706	In only Ara
bll7287	Q89E00	*dgoA*	2-dehydro-3-deoxyphosphogalactonate aldolase.	-1.4610	-
			*NAD and FAD production*		
blr0430	Q89X84	*nadD*	Probable nicotinate-nucleotide adenylyltransferase.	-1.3324	-
bll2541	Q89S64	*nadB*	L-aspartate oxidase.	In only Ara	-1.7782
bll4547	Q89LJ7	*nadE*	Glutamine-dependent NAD(+) synthetase.	-1.2616	-
bll7452	Q89DI7	*ribH2*	6,7-dimethyl-8-ribityllumazine synthase 2.	In only Ara	-
blr8123	Q89BM5		MutT/nudix family protein.	-1.3450	NS
			*PHB synthesis and degradation*		
bll0225	Q89XT2	*phaB*	Acetoacetyl CoA reductase.	2.7381	NS
blr0227	Q89XT0	*phaR*	Transcriptional regulator of polyhydroxybutirate synthesis.	1.6828	NS
blr0908	Q89VY7		Hypothetical protein. Similar to PHB depolymerase.	In only Ara	-2.0085
bll4360	Q89M33	*phaC1*	Poly-3-hydroxybutyrate synthase.	2.7271	NS
bll6073	Q89HC1	*phaC2*	Polyhydroxybutirate synthase PhaC2.	In only Ara	NS
			*Biotin synthesis*		
blr2097	H7C6S8	*bioF*	8-amino-7-oxononanoate synthase.	-	In only Mtl
blr2098	Q9AMS4	*bioD*	ATP-dependent dethiobiotin synthetase.	In only Mtl	-
blr2099	H7C6S2	*bioA*	Adenosylmethionine-8-amino-7-oxononanoate aminotransferase.	In only Mtl	-
blr2221	Q89T29	*bioA*	Adenosylmethionine-8-amino-7-oxononanoate aminotransferase	1.1619	-
bll6273	Q89GS2		Probable biotin sulfoxide reductase.	In only Ara	-
			*Cell motility and chemotaxis*		
blr2343	Q89SQ7	*cheA*	Chemotaxis two-component sensor histidine kinase.	NS	-1.8082
blr2344	Q89SQ6	*cheWII*	CheWII protein.	In only Ara	NS
blr2345	Q89SQ5	*mcpK*	Methyl-accepting chemotaxis protein.	NS	-1.8623
blr2346	Q89SQ4	*cheW*	CheW protein.	In only Ara	-
blr2347	Q89SQ3		Methyl-accepting chemotaxis protein.	NS	-1.5919
blr2349	Q89SQ1	*cheB*	Chemotaxis response regulator protein-glutamate methylesterase of group 3 operon.	-	In only Ara
blr2931	Q89R41		Putative methyl-accepting chemotaxis protein.	-	In only Ara
bll6858	Q89F43	*flgE_L_*	Lateral flagellar hook protein	In only Ara	-
bll6865	Q89F36	*lafA2*	Lateral flagellin 2	In only Ara	In only Ara
bll6866	Q89F35	*lafA1*	Lateral flagellin 1	In only Ara	In only Ara

*The proteins were found in the cytoplasmic-enriched fraction (Cytoplasm column) or in the membrane-enriched fraction (Membrane column). The log_*2*_ fold-change values of the Mtl/Ara ratio of the areas under the chromatogram peaks were considered significant when they were > 1 (at least 2-fold upregulated in HMY-Mtl) or < -1 (at least twofold upregulated in HMY-Ara), with a Student-*t*-test *p* < 0.05. In addition, those proteins identified under only one nutritional condition are indicated. Otherwise, certain proteins were represented differently in one of the fractions, but not significantly so (NS) in the other. Only those proteins identified in at least two biologic replicas are included. The descriptions are based on data from Rhizobase, Uniprot, KEGG, and our previous work*.

### Catabolism of Mtl and Ara

**FIGURE 1:**
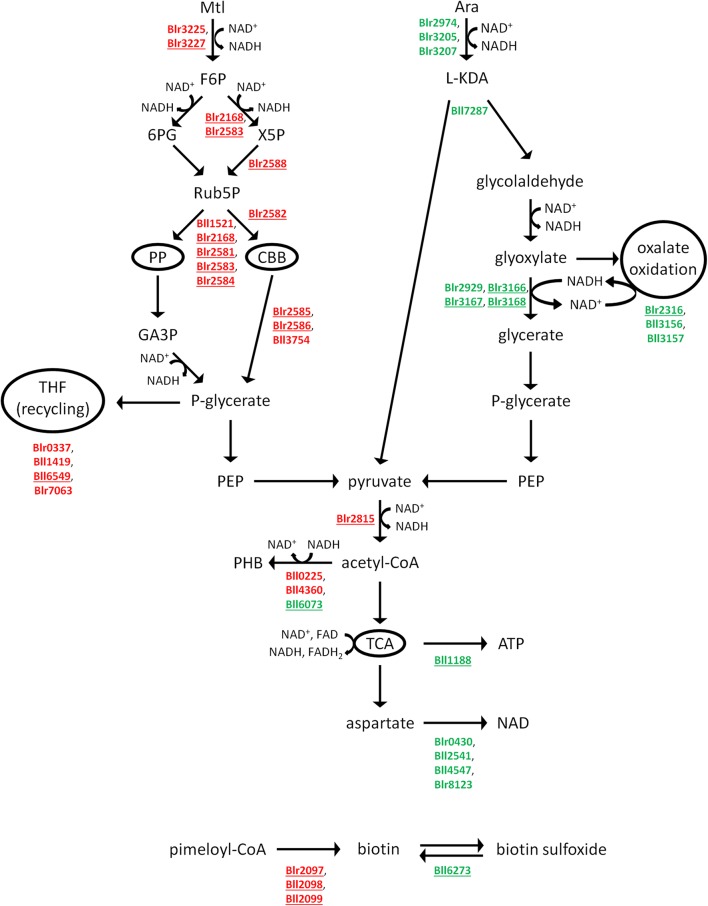
Summary of the Mtl **(left)** and Ara **(right)** catabolic pathways. The pathways were deduced from our proteomics data, while all the enzymes catalyzing these reactions were detected in our samples. The enzymes that appeared as differential between Mtl and Ara are indicated by their annotation in Rhizobase, with those in red belonging to the MtlDP and those in green to the AraDP. The enzyme indications underlined are present under only one culture condition. PP, pentose-phosphate pathway; CBB, Calvin–Benson–Bassham pathway; THF, tetrahydrofolate; TCA, tricarboxylic-acid cycle.

Mtl and Ara appeared to be catabolized by the two rather different pathways that are summarized in **Figure 1**. The proteome of the Mtl-grown cells contained two salient features: (i) the lack of phosphofructokinase (Bll2580 and Blr4659; EC 2.7.1.11; Supplementary Table S1), indicating, in agreement with previous reports, that the Emden–Meyerhof–Parnas pathway is inactive under this condition (Sosa-Saavedra et al., 2001), and ii) the strong induction of phosphoribulokinase (Blr2582; EC 2.7.1.19) along with both subunits of ribulose-bisphosphate carboxylase-oxygenase (RuBisCO; EC 4.1.1.39), the latter encoded by the *cbbL* (blr2585) and *cbbS* (blr2586) loci. Because these enzymes are key members of the CBB cycle, their presence in only the MtlDP suggested that the carboxylation and/or oxygenation of ribulose 1,5 bisphosphate might have been taking place in the HMY-Mtl cultures. Five other enzymes were present in only the MtlDP that catalyzed reactions shared by the pentose-phosphate pathway (PP) and the CBB cycle (**Figure 1** and **Table 2**). In addition, the Mtl-dehydrogenase activity, which catalysis is required to oxidize Mtl to D-fructose, was measured experimentally (Kuykendall and Elkan, 1977) but the gene encoding that enzyme was not annotated in the *B. diazoefficiens* genome. We observed that Blr3225, which enzyme is similar to the glucose/ribitol oxidoreductase, is present in the MtlDP. The corresponding structural gene is a neighbor of three genes encoding proteins also found in MtlDP—namely the putative sorbitol/Mtl transport system (blr3224), a D-ribitol kinase (EC 2.7.1.47; blr3226), and the D-fructokinase (EC 2.7.1.4; blr3227) that may be required to phosphorylate the D-fructose produced after Mtl reduction (**Table 2**). Therefore, we would suggest that Blr3225 is the missing inducible Mtl-dehydrogenase EC 1.1.1.67 described by Kuykendall and Elkan (1977). In addition, two other enzymes in the MtlDP cytoplasm-enriched fraction are also present at similar levels in the membrane-enriched fractions from HMY-Mtl and HMY-Ara cultures. Those proteins, which are the (putative) fructose-1,6-bisphosphate aldolase (Bll1521; EC 4.1.2.13) and the phosphoglycolate phosphatase (Bll3754; EC 3.1.3.18), might also carry out functions in HMY-Ara metabolism.

**FIGURE 2:**
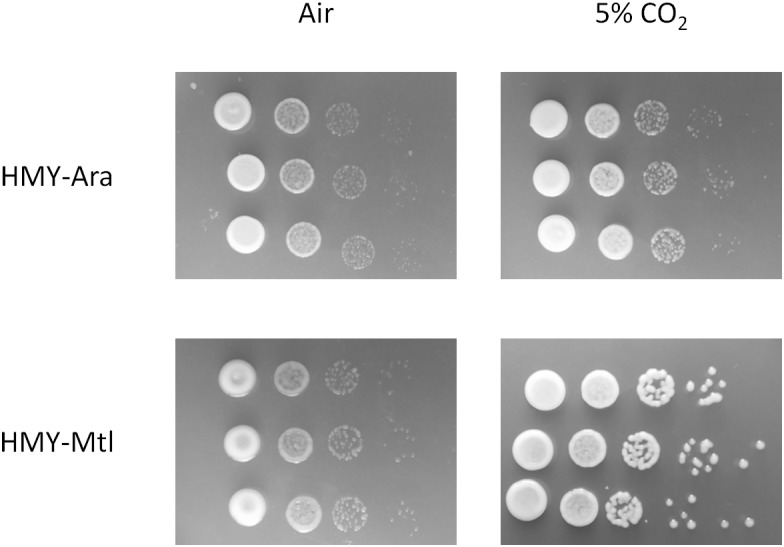
Effect of CO_2_ on the growth of *B. diazoefficiens.* The bacteria were grown in liquid HMY-Ara or HMY-Mtl and serially diluted 1/10 and then 5-μl drops were inoculated in the same medium supplemented with 1.5% (w/v) agar. The bacteria were incubated for 7 days in contact with regular air, or with air enriched with 5% CO_2_. From left to right, increasing serial dilutions.

Data from the literature indicate that *B. diazoefficiens* is able to fix CO_2_ through the CBB pathway (Hanus et al., 1979; Masuda et al., 2010), and that the affinity ratio of the *B. diazoefficiens* RuBisCO for CO_2_ and O_2_ is in the order of that of higher plants (Horken and Tabita, 1999). To ascertain whether *B. diazoefficiens* possessed activity of the RuBisCO carboxylase when grown in HMY-Mtl, we compared the development of bacterial colonies in solid HMY-Mtl or HMY-Ara, either in a normal atmosphere, or in one enriched with 5% (v/v) CO_2_ (**Figure 2**). In a normal atmosphere the HMY-Mtl colonies had a slightly higher degree of mucoidy than the HMY-Ara colonies; but the colonies developed in HMY-Mtl in the atmosphere with 5% CO_2_ were substantially more mucoid, indicating that under this condition *B. diazoefficiens* USDA 110 produced more EPS. In contrast, the colonies formed in HMY-Ara at 5% CO_2_ were indistinguishable from those developing in the normal atmosphere, thus further corroborating that the changes observed were restricted to the Mtl-growth condition.

Ara degradation, for its part, is carried out by the L-KDA pathway—similar to the Entner–Doudoroff scheme—coupled to oxalate oxidation (Watanabe et al., 2006; Koch et al., 2014). Accordingly, 11 enzymes of those pathways were found in the AraDP pool (**Table 2**). The L-KDA sequence bifurcates into the pyruvate and glycolaldehyde branches upon the formation of the L-KDA intermediate, the latter being further oxidized to glyoxylate (**Figure 1**). Whereas pyruvate is decarboxylated to acetyl-CoA, glyoxylate may be in part converted to oxalate and oxidized to CO_2_ through the participation of formyl-CoA:oxalate CoA-transferase (Frc, Bll3156; EC 2.8.3.16) and oxalyl-CoA carboxy-lyase (Oxc, Bll3157; EC 4.1.1.8). Another portion of the glyoxylate pool may be converted to glycerate through reaction with the NADH formed by oxalate oxidation and then continue on the pathway to pyruvate (**Figure 1**). Among the differentially expressed proteins, the Oxc was present in the AraDP cytoplasm-enriched fraction and was also observed at similar levels in the Ara and Mtl membrane-enriched fractions (**Table 2**). Therefore, the activity of this enzyme might be required in concert with that of the Frc—which enzyme, though being more abundant in the AraDP cytoplasm-enriched fraction, was absent in the MtlDP membrane-enriched fraction—in order to detoxify intracellular oxalate, which detoxification might also be required in cells grown on HMY-Mtl.

### Stoichiometric Balances of ATP and Pyruvate Production and of O_2_ Consumption

We obtained the balances of potential ATP production and O_2_ consumption per C-mole of substrate to estimate the efficiency of energy generation by each one of the pathways schematized in **Figure 1** as well as the production of pyruvate before that intermediate enters the TCA-cycle in order to assess the anaplerotic potential of the metabolic flux in that direction. To compare the energetic potentials of these pathways, we assumed that all the C-source is fully oxidized with maximum energy recovery as ATP, and assumed maximal P/O values of 3 for NADH and 2 for FADH_2_. In addition, we assumed that all the NADH and FADH_2_ produced in these pathways transfer their electrons to O_2_, which under the present circumstances, would be the final electrons acceptor.

The energetic balance of the deduced Mtl catabolic pathway was difficult because, in lacking direct enzyme-activity measurements, we could not estimate to what extent the PP and CBB pathways were operating and, if the CBB pathway was in fact active, what the proportion of carboxylase *versus* oxygenase activity was in the RuBisCO catalysis. Therefore, we obtained the pathways from KEGG with the enzymes that we actually detected in our samples—independently of whether they were differentially expressed or not—and calculated the stoichiometries taking into account all three possibilities, as summarized in **Table 3**. If, as one possibility, the RuBisCO were not functional, we would expect that Mtl was catabolized by the PP pathway, as shown in Supplementary Figure S2, in which circumstance the stoichiometry can be readily calculated (Supplementary Table S6). If, alternatively, the RuBisCO were functional, the question would remain regarding the relative participation of the carboxylase *versus* the oxygenase activity, which consideration we approached by establishing three different scenarios: oxygenase activity alone, 50% of each activity, or carboxylase activity alone. When we tried to calculate the stoichiometries assuming oxygenase as the sole activity in the RuBisCO catalysis, we found an imbalance of three C atoms, which discrepancy we could compensate for in two ways: either by delivering the glycolate produced in the ribulose-1,5-bisphosphate oxygenation to the oxalate-oxidation pathway (Supplementary Figure S3), or by recycling part of the C atoms through the THF pathway (Supplementary Figure S4). This latter possibility was suggested by the induction of Bll6549 (**Table 2**); which locus, according to the KEGG Pathways, encodes a D-3-phosphoglycerate: NAD^+^ oxidoreductase (EC 1.1.1.95) catalyzing the conversion of D-3-phosphoglycerate into 3-phosphonooxypyruvate, and that product may then enter the THF pathway after transaminations from serine and glycine (Supplementary Figure S4). Supplementary Tables S7, S8, respectively, summarize the resulting stoichiometries. In addition to the higher yield of ATP and pyruvate upon THF-recycling (**Table 3**), we wish to specify that the oxalate oxidation requires at least two enzymes that are in the AraDP (Supplementary Table S7). Although these two enzymes are also present in Mtl-grown cultures (**Table 2**), all these results indicate that C-recycling through the THF pathway should be more likely than the direct glycolate oxidation through the oxalate-oxidation pathway. Nevertheless, we maintained the same set of alternative possibilities when we considered that RuBisCO catalysis involved 50% carboxylase and 50% oxygenase activities, as exemplified in Supplementary Figures S5, S6, which postulation led to the stoichiometries of Supplementary Tables S9, S10. Finally, the assumption of a sole RuBisCO-carboxylase activity (Supplementary Figure S7) led to a pathway that did not produce glycolate, so that all the carbon atoms of ribulose 1,5-bisphosphate, together with the fixed CO_2_, were converted to glycerate, whose further metabolism to pyruvate would enable the possibility of entering into the TCA-cycle (Supplementary Table S11).

**Table 3:** Stoichiometric balances for maximal ATP production, O_2_ consumption, and pyruvate production before this intermediate enters the TCA cycle.

C-source	Proposed pathway^a^	Production/consumption (mole.C-mole of substrate^-1^)		
		ATP	O_2_	Pyruvate
Mtl	PP	6.7	1.1	0.2
	CBB (O-OXA)	4.2	1.2	0.1
	CBB (O-THF)	4.4	1.2	0.3
	CBB (CO-OXA)	5.3	1.2	0.2
	CBB (CO-THF)	5.4	1.1	0.3
	CBB (C)	6.4	1.1	0.3
Ara	L-KDA	5.2	1.1	0.3

^a^*The catabolic pathways were equilibrated upon consideration of the possibility that Mtl was catabolized through either the PP, or the CBB pathway. In the latter instance, different models were proposed according to whether the RuBisCO acted as an oxygenase alone (O), an oxygenase plus a carboxylase (CO), or a carboxylase alone (C): If an oxygenase activity, part of the carbons could be either oxidized in the oxalate pathway (OXA) or recycled through the tetrahydrofolate (THF) pathway. For further details, *cf*. Supplementary Tables S6–S12*.

Regarding the L-KDA pathway in the Ara cultures, we confirmed the existence of the pathway deduced by Koch et al. (2014) and propose that the glycerate originating in one of the metabolic branches from glyoxylate is converted to pyruvate, which intermediate would then enter the TCA-cycle (Supplementary Figure S8). This proposal results in the construction of the stoichiometry of Supplementary Table S12.

According to these stoichiometric calculations, the potential yield of ATP was, at the most, 1.3 times higher in the Mtl- than in the Ara-catabolism calculations, whereas the potential O_2_ consumption per C-mole of substrate was, at the most, 1.1 times higher in the Mtl than in the Ara calculations (**Table 3**). Furthermore, a high pyruvate production per C-mole of substrate was calculated in the Ara-catabolism calculations, which C yield might indicate a certain anaplerotic potential.

### NAD and FAD Production

Four proteins related to the synthesis of nicotinamide-adenine dinucleotide (NAD) from L-aspartate are present in AraDP, although all of those gene products are simply upregulated; indicating that, as may be expected, none is completely absent from the HMY-Mtl cultures. (**Figure 1** and **Table 2**). In addition, the 6,7-dimethyl-8-ribityllumazine synthase 2 (EC 2.5.1.78), which enzyme is involved in the synthesis of flavin-adenine dinucleotide (FAD), was observed in only the AraDP cytoplasmic-enriched fraction (**Table 2**). In parallel, one paralog of the A-chain of the ATP synthase (Bll1188) was observed in only the membrane-enriched fraction of the AraDP (**Table 2**). These results indicate that a higher production of electron acceptors might occur under these conditions, suggesting the possibility of a higher electron flux to the ETC in the HMY-Ara cultures than in the HMY-Mtl.

In contrast to these consistent trends, the changes in the ETC proteins were scattered (Supplementary Table S5), indicating either that we could not adequately separate these membrane-embedded proteins for MS analysis, or that a possible higher electron-flux through the ETC might be attained without major changes in the abundance of the ETC proteins.

### PHB Synthesis and Degradation

Four proteins of the PHB pathway were found as differentially expressed in the cytoplasmic-enriched fractions between the two culture conditions. The regulator PhaR (Blr0227; Quelas et al., 2016b), the acetoacetyl-CoA reductase PhaB (Blr0225; EC 1.1.1.36), and the PHB synthase PhaC1 (Bll4360; EC 2.3.1.-)—all of which are involved in PHB synthesis—were found upregulated in MtlDP; whereas the inactive PhaC2 paralog of PHB synthase (Blr6073; Quelas et al., 2013) was present in only the cytoplasmic-enriched fraction of the AraDP (**Table 2**). In addition, the hypothetical protein Blr0908, which predicted gene product has more than a 65% identity to rhizobial PHB depolymerases, was likewise found in the AraDP in both the cytoplasmic- and the membrane-enriched fractions (**Table 2**). To our surprise, we also observed the truncated PHB synthase PhaC4 (Blr2885)—which protein appears to be inactive in PHB metabolism (Quelas et al., 2013)—in only the membrane-enriched fraction of the MtlDP (Supplementary Table S2).

If the PHB granules had coprecipitated with the membranes (Jendrossek and Pfeiffer, 2014), the presence of the proteins in the membrane-enriched fractions may actually be reflecting the fraction bound to PHB granules. Hence, we measured the PHB contents of HMY-Mtl- or HMY-Ara–grown cells in the stationary phase (OD_500__nm_ = 6.7 in the HMY-Mtl and 4.8 in the HMY-Ara cultures) and observed that HMY-Mtl-grown cells had 20.7 ± 0.6 fg of PHB.CFU^-1^, whereas the HMY-Ara-grown cells had 13.7 ± 1.2 fg.CFU^-1^, thus corroborating that the PHB synthesis and accumulation was higher under growth in HMY-Mtl.

### Biotin Synthesis

Three proteins related to biotin biosynthesis were observed in only the MtlDP: 8-amino-7-oxononanoate synthase (Blr2097; EC 2.3.1.47) in the membrane-enriched fraction plus an ATP-dependent dethiobiotin synthetase (Blr2098; EC 6.3.3.3) and adenosylmethionine-8-amino-7-oxononanoate aminotransferase (Blr2099 and Blr2221; EC 2.6.1.62) in the cytoplasmic-enriched fraction—with the last of these (i.e., Blr2221) upregulated in that fraction. By contrast, in the AraDP a probable biotin-sulfoxide reductase encoded in bll6273 could be observed in only the cytoplasmic-enriched fraction (**Table 2**). These results suggested that a more active synthesis of biotin occurred in the HMY-Mtl-grown cells; whereas in the HMY-Ara cultures an accumulation of oxidized biotin would seem prevented (**Figure 1**). Biotin is a cofactor of various carboxylases, among which are the EC 6.4.1 ligases that catalyze the formation of carbon-carbon bonds (Erb, 2011). We observed two such carboxylases in the MtlDP: a putative propionyl-CoA carboxylase beta chain (Blr3940; EC 6.4.1.3) was present in only the membrane-enriched fraction, and a 3-methylcrotonyl-CoA carboxylase (alpha subunit Blr 4421 along with a beta subunit Blr0986; EC 6.4.1.4) was upregulated in both the cytoplasmic- and membrane-enriched fractions (Supplementary Table S2). In contrast, no carboxylases were detected in the AraDP.

The presence of the probable biotin-sulfoxide reductase in the AraDP cytoplasmic-enriched fraction suggested a higher oxidation rate of biotin. Therefore, upon searching for possible oxidative-damage–protecting proteins in the MtlDP and AraDP, we found two related to oxidative-damage mitigation in the MtlDP, and six in the AraDP (Supplementary Table S5). Their functions are somewhat superimposed, however; and in two instances the proteins that appeared as differentially expressed in one subcellular fraction were at similar levels in the other. In addition, since many paralogs exist for these functions, whether or not those differences are reflected in a true differential metabolism directed toward oxidative-damage mitigation remains unclear.

### Cell Motility and Chemotaxis

In agreement with our previous observations (Covelli et al., 2013; Mongiardini et al., 2017) and the results presented in Supplementary Figure S1B, we found the lateral-flagellar–filament structural flagellins Bll6865 and Bll6866, as well as the lateral-flagellar–hook protein Bll6858 in the AraDP (**Table 2**), but we failed to detect the majority of the other lateral-flagellar components. Nevertheless, we recently found the transcripts for these proteins produced in the HMY-Ara but not in the HMY-Mtl, under the control of the *lafR* transcriptional regulator (Mongiardini et al., 2017). Because flagellins and the flagellar-hook protein are the most abundant flagellar gene products, the lack of detection of the other proteins might have resulted from a low abundance or from a tight association with the flagellar structure that perhaps we did not manage to disassemble during the extraction. Indeed, we detected the rotor protein FliN_L_ (Bll6879) and the stator protein MotA (Bll6882)—but in only one sample each—in the HMY-Ara cultures (Supplementary Table S1). Moreover, the secreted flagellins observed in Supplementary Figure S1B require that at least the flagellar-export apparatus and the hook be assembled (Evans et al., 2014).

In addition to the lateral-flagellar structural proteins, we detected most of the proteins of one of the chemotaxis clusters in the AraDP. These proteins comprise those encoded in blr2343 to blr2347 plus blr2349—which loci are part of a functional chemotaxis cluster (Donati et al., 2011)—along with the putative methyl-accepting chemotaxis protein encoded in blr2931 (**Table 2**). This finding was somewhat surprising because the lateral flagella seemed not to respond to known chemotaxis signals, and Ara is not a strong chemoattractant for this strain (Quelas et al., 2016a). Moreover, although these proteins were detected in the cytoplasmic-enriched fraction, the majority were differential in the membrane-enriched fraction (**Table 2**). In particular, the functioning of the methyl-accepting chemotaxis proteins (Blr2345 and Blr2347) should occur in the membrane compartment.

### Analysis of Growth and O_2_-Consumption Rates

According to **Table 3**, the efficiency of ATP production per C-mole of substrate (*y*_ATP/S_), as well as the balance of O_2_ consumption per C-mole of substrate would have been similar in the HMY-Mtl or HMY-Ara cultures. Nonetheless, earlier studies on the respiration of soybean-nodulating rhizobia had demonstrated that the O_2_-consumption rate of the strain 505 was higher in Ara- than in Mtl-grown cells (Thorne and Burris, 1940). In agreement with that report, in the present work, we detected that in HMY-Ara cultures an activation of NAD- and FAD-biosynthetic enzymes, a reduction of PHB accumulation, and an increase in the biotin-reducing activity occurred, all of which observations would indicate that HMY-Ara–grown cells might harbor a shortage of reducing power and a surplus of oxidative species relative to HMY-Mtl–grown rhizobia. We therefore calculated the *qO*_2max_ and other growth parameters of *B. diazoefficiens* USDA 110 growing in HMY-Mtl or HMY-Ara in independent duplicate bioreactor batch cultures during the exponential-growth phase. The specific growth rate in the exponential phase (*μ*_max_) proved to be not significantly different between those two carbon sources, but the *qO*_2max_ of the wild-type strain was almost three times higher in the HMY-Ara than in the HMY-Mtl (**Table 4**), thus corroborating the results obtained by Thorne and Burris (1940). In addition, the *y*_x/s_ was two times higher in the HMY-Mtl (**Table 4**). In agreement with these observations, the cultures in HMY-Ara entered the stationary phase of growth at around 40–50 h of culture, while those in HMY-Mtl were continuing in the exponential phase even after 70 h, thus producing a substantially higher biomass by this time (data not shown).

**Table 4:** Specific rates (±SD) of substrate consumption, product formation, and growth yields of *B. diazoefficiens* growing in batch cultures in the exponential growth phase.

Property^a^	Wild-type			*lafR*::Km mutant		
	HMY-Mtl	HMY-Ara	Ratio Ara/Mtl	HMY-Mtl	HMY-Ara	Ratio Ara/Mtl
*μ*_max_ (h^-1^)	0.04 ± 0.01	0.04 ± 0.02	1.0	0.03 ± 0.01	0.03 ± 0.01	1.0
*qO*_2max_ (mmol.g^-1^.h^-1^)	0.86 ± 0.02	2.53 ± 0.11	2.9	1.26 ± 0.12	1.63 ± 0.07	1.3
*y*_x/s_ (C-mole.C-mole^-1^)	0.50 ± 0.10	0.27 ± 0.08	0.5	0.52 ± 0.13	0. 41 ± 0.07	0.8
*qS _max_* (mC-mole.g^-1^ .h^-1^)	2.81 ± 0.46	7.66 ± 0.47	2.7	2.17 ± 0.34	2.90 ± 0.64	1.3
*qCO_2__max_* (mmole.g^-1^ .h^-1^)	0.67 ± 0.03	2.39 ± 0.05	3.6	0.60 ± 0.01	1.05 ± 0.08	1.7

^a^*μ_*max*_, specific growth rate in the exponential phase; *qO_*2 max*_*, specific rate of O_*2*_ consumption; *y_*x/s*_*, biomass yield per C-mole of substrate consumed; *qS_*max*_*, the specific rate of substrate consumption; *qCO_*2 max*_*, the specific rate of CO_*2*_ production; mC-mole, 10^-*3*^ C-moles*.

In accordance with the *qO*_2max_ values, the specific rates of substrate consumption (*qS*_max_) and CO_2_ production (*qCO*_2max_) also were around three times higher in the cultures of *B. diazoefficiens* in the HMY-Ara than in those in the HMY-Mtl (**Table 4**). Since the amount of biomass produced per mole of ATP (*y_x/ATP_*) was the same for both culture conditions and the stoichiometric balances failed to reveal any substantial differences in the efficiency of ATP production per C-mole of substrate, the results would indicate that the cultures in the HMY-Ara consumed a larger proportion of the catabolic energy produced for simple maintenance—encompassing processes different from the production of biomass or C-products—than did those in the HMY-Mtl. One of these maintenance processes requiring energy may be the generation and use of the lateral flagella, which appendages are present in the HMY-Ara cultures but are scanty in the HMY-Mtl cultures (Mongiardini et al., 2017; see also Supplementary Figure S1B). To evaluate the contribution of the lateral flagella to *qO*_2max_ in HMY-Ara, we repeated the above measurements in both culture media with a *B. diazoefficiens* USDA 110 *lafR*::Km insertional mutant lacking the class IB regulator of lateral-flagellum synthesis and therefore unable to produce any lateral flagellar structures, including the flagellar motor (Mongiardini et al., 2017). We observed that *μ*_max_ remained within the same values; but in contrast to the considerable elevation in O_2_-consumption on the part of the wild strain in HMY-Ara, the *qO*_2max_ of the *lafR*::Km mutant was similar in both the HMY-Ara and the HMY-Mtl (**Table 4**), thus indicating that the absence of lateral flagella substantially reduced the enhancement in O_2_-consumption rate with Ara as the sole C-source. In agreement with these results, the values of *y*_x/s_, *qS*_max_, and *qCO*_2max_ in the *lafR*::Km mutant were similar upon growth in the HMY-Mtl and the HMY-Ara (**Table 4**); but entry into the stationary phase remained earlier in the HMY-Ara cultures than in the HMY-Mtl.

### Ability of the Mutant Devoid of Lateral Flagella to Outcompete the Wild Type in Coculture

The differences in *qO*_2max_ and *y*_x/s_ among the wild-type and the *lafR*::Km mutant in HMY-Ara (**Table 4**) suggested that this mutant might outcompete the wild-type if both strains were to grow together. To investigate this possibility, we coinoculated the strains either in HMY-Ara or HMY-Mtl, initiating the cultures with 1:1 admixtures containing *ca*. (7.5 ± 0.2).10^6^ CFUs.ml^-1^ of each strain at time zero. We left the cultures until they reached the stationary phase and then counted the numbers of CFUs.ml^-1^ using different antibiotic resistances to distinguish the wild-type and the mutant. As controls, we maintained parallel cultures of each strain alone without competition [initial CFU ml^-1^ of each strain alone at time zero: (1.5 ± 0.2).10^7^] and observed that 100% of the CFUs obtained from those populations carried the antibiotic-resistance mark of the corresponding strain, indicating that no antibiotic-resistant spontaneous mutants arose in significant numbers during the course of the experiment.

**FIGURE 3:**
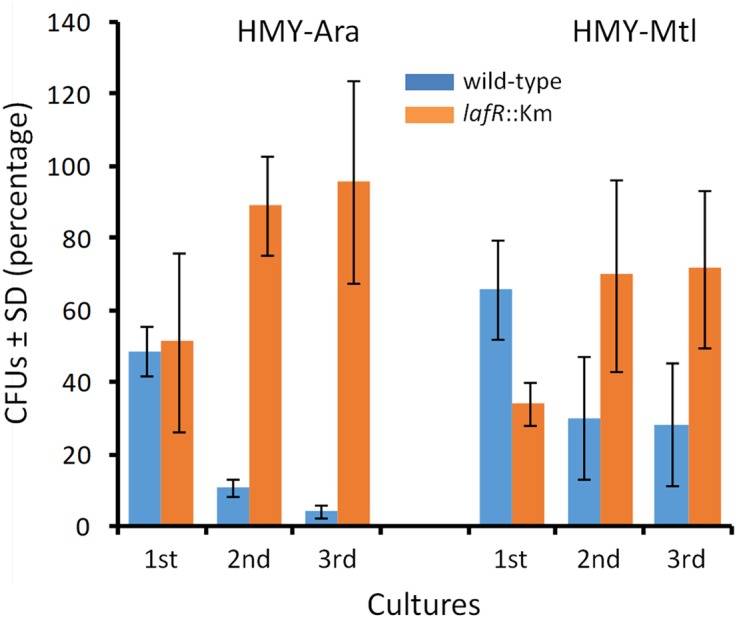
*Bradyrhizobium diazoefficiens* bacteria with or without lateral flagella competing in three successive cultivations. The wild-type strain and a *lafR*::Km mutant devoid of lateral flagella were inoculated together, in 1:1 admixures containing *ca*. (7.5 ± 0.2).10^6^ CFUs.ml^-1^ of each strain at time zero in the indicated media and then grown until stationary phase for the first cultures; from these initial cultures, 1/100 dilutions were made in the same medium for establishing the second cultures; and finally that entire process was repeated to generate the third cultures. Once each culture attained the stationary phase, samples were removed, and the colony-forming units (CFUs) per ml of each strain—distinguished through the use of differential antibiotic-resistance labels—were counted in the selective media. In the figure, the relative number of CFUs between the two strains (blue bars, wild-type; orange bars, mutant), expressed as a percent ± the SD, are plotted on the *ordinate* for each of the successive cultures indicated on the *abscissa* in the corresponding medium denoted above the bars. In parallel controls containing each strain alone (not shown), the CFU numbers were equivalent, so that none of the CFUs registered in that control contained the antibiotic-resistance label of the other strain; thus indicating that during the course of the experiment no losses of intrinsic viability occurred under each selective growth condition and that the potential appearance of spontaneous antibiotic-resistant mutants was not significant.

At the time of sampling for the CFU counting of the cocultures, we also diluted these stationary-phase cells to initiate new cultures, repeating the initial process twice in order to strengthen the competition among the strains in the second and third subcultures. Thus, we observed that after replicating the bacterial admixtures in successive cultures, the *lafR*::Km mutant clearly outcompeted the wild-type strain in HMY-Ara, reaching more than 95% of the cell population by the third subculture (**Figure 3**). Higher numbers of the mutant cells, however, also developed in the HMY-Mtl, but in this instance the competition was not so pronounced as in the HMY-Ara. These results were not attributable to intrinsic differences in survival since in the controls where the strains were cultured axenically the total number of CFUs.ml^-1^ remained constant throughout the successive subcultures (not shown).

## Discussion

Our results have demonstrated that the growth of *B. diazoefficiens* with either Mtl or Ara as the sole C-source leads to pronounced differences in the bacterium’s metabolic and physiologic properties, even when either nutrient was highly efficacious in terms of growth rate and biomass production.

Among the differential phenotypes observed in HMY-Mtl was a higher mucoidy, especially in a CO_2_-rich atmosphere (**Figure 2**). At the molecular level, a significant induction of RuBisCO and phosphoribulokinase occurred in HMY-Mtl (**Figure 1**), with both enzymes constituting key steps in the CBB pathway. These observations suggested that *B. diazoefficiens* was able to use atmospheric CO_2_ to increase the bacterium’s EPS content, although at present we cannot estimate the proportion of carboxylase activity in the catalysis by the *B. diazoefficiens* RuBisCO under our culture conditions. Nevertheless, even though our stoichiometric calculations indicated that no net CO_2_ consumption occurred, what was yet remarkable to us was that this strain still had a functioning carboxylase activity in the rich HMY-Mtl medium containing an abundant carbon source—that finding in contrast to previous reports stating that chemoautotrophic growth conditions are required for RuBisCO activation either using H_2_ under microaerobic conditions (Hanus et al., 1979; Franck et al., 2008) or thiosulfate in the air (Masuda et al., 2010) as electron donors. This activity might well be advantageous in the edaphic atmosphere, where the CO_2_ concentration is 10–50 times higher than in the air (Lamborg et al., 1983). In addition, the greater amount of EPS formed in HMY-Mtl—and especially in a CO_2_-rich soil atmosphere—would very likely favor the adhesion of the bacteria to the plant-root surfaces along with the formation of biofilms in the rhizosphere (Pérez-Giménez et al., 2009; Quelas et al., 2010).

Furthermore, we observed that, in addition to EPS, *B. diazoefficiens* also produced more PHB in HMY-Mtl than in HMY-Ara. In *B. diazoefficiens*, PHB production increased under unbalanced growth conditions, especially when the cell density was high-which may implicate high reducing power-and carbon source were in excess (Quelas et al., 2013, 2016b). We had previously found that the regulator PhaR was required for PHB synthesis in *B. diazoefficiens* (Quelas et al., 2016b) and that PHB regulation may be exerted also at the level of enzyme activity. The PHB synthase PhaC, which enzyme in general is active as a dimer (Rehm, 2003), has five paralogs in *B. diazoefficiens*, among which PhaC1 and PhaC2 are the most relevant subunits (Quelas et al., 2016b). Whereas PhaC1 is active, PhaC2 is not, and probably the relative abundance of the possible dimers PhaC1/PhaC1, PhaC1/PhaC2, or PhaC2/PhaC2 determines the overall PHB-synthase activity (Quelas et al., 2013, 2016b). In the present work, we observed that the PhaR regulator was itself upregulated in HMY-Mtl; whereas the expressions of PhaC1 and PhaC2 were inversely regulated between the two media, with PhaC1 being upregulated in HMY-Mtl and PhaC2 upregulated in HMY-Ara. In addition, the acetoacetyl-CoA reductase PhaB, which enzyme catalyzes the second step in the PHB biosynthetic pathway (Jendrossek and Pfeiffer, 2014), was likewise upregulated in HMY-Mtl; while the enzyme Blr0908—it being similar to the PHB depolymerases—conversely, was upregulated in HMY-Ara. These results suggest that the lower *qO*_2max_ in HMY-Mtl might lead to a channeling of part of the surplus of reducing power into PHB synthesis.

In this regard, we furthermore corroborated that this bacterium has a *qO*_2max_ three times higher when growing in HMY-Ara than in HMY-Mtl, a phenomenon for which we conceived of two alternative explanations. The first possibility would be that cells grown in HMY-Ara possess lateral flagella—although we do not know exactly how many exist per cell—whereas the same bacteria cultured in HMY-Mtl scarcely produce those extracellular appendages (Mongiardini et al., 2017). Because lateral flagella seem powered by the same proton-motive force that drives ATP synthase (Kanbe et al., 2007), the flagellar rotation in HMY-Ara might compete with ATP synthesis, thus requiring a higher *qO*_2max_ in HMY-Ara than in HMY-Mtl to maintain the equivalent *μ*_max_, exemplified in **Table 4**. Alternatively, the differences in the catabolic pathways deduced from the proteomic analysis might imply that *y*_ATP/S_ from Ara could be less efficient than from Mtl, thus requiring a higher rate of ATP production per unit time. Our stoichiometric calculations of maximal theoretical *y*_ATP/S_ suggest that this latter possibility may be ruled out, so that the hypothesis that the high *qO*_2max_ results from the generation and functioning of the lateral flagella seems plausible. In agreement with this hypothesis, we observed that the *lafR*::Km mutant devoid of lateral flagella had similar *qO*_2max_ values in HMY-Ara and HMY-Mtl, and outcompeted the wild strain in coculture. This overgrowth, though certainly occurring to some extent in HMY-Mtl, was especially evident in HMY-Ara, suggesting that either the low *lafR* expression in Mtl (Mongiardini et al., 2017) produced some lateral flagellar structures, or another physiologic difference(s) enhanced the mutant’s competition in that C-source. Nevertheless, the differences in competition attributable to the absence of lateral flagella were significantly higher in HMY-Ara, indicating that the energy consumption by those organelles was a principal component in the ability of the mutant to overgrow the wild-type.

Despite these results and considerations, we have difficulty in assessing whether lateral-flagellum expression is the cause or the consequence of the higher *qO*_2max_ in HMY-Ara, because the data from the literature would indicate that lateral-flagellum synthesis is also stimulated in response to permanent exposure to moderate oxidative stress (Donati et al., 2011), or is inhibited in microaerobiosis (Pessi et al., 2007) or iron deficiency (Yang et al., 2006), pointing out that the oxygenation and/or respiratory status of the cell is a key element in lateral-flagellum control. Our results indicate that the increased *qO*_2max_ in HMY-Ara is not independent of the presence of the lateral flagella, thus suggesting that the synthesis of those organelles might be the cause of, rather than the response to, the high *qO*_2max_.

In addition, other species of the genus *Bradyrhizobium*, such as *B. elkanii*, do not possess lateral flagella (Quelas et al., 2016a), and yet they dwell in the same soil habitat and establish comparable symbiotic interactions with the soybean as does *B. diazoefficiens*. A similar situation transpires in the close relative *Rhodopseudomonas palustris*, a non-sulfur purple bacterial species in which strains possessing or lacking a similar secondary flagellar system coexist in the same small portion of sediment (Oda et al., 2008). Moreover, mutant strains of *B. diazoefficiens* lacking lateral flagellins were found to be more competitive than the wild-type in nodulating the soybean or in colonizing the soybean rhizosphere (Althabegoiti et al., 2011). Furthermore, the lateral flagella seem not to be contributing to the swimming speed or chemotaxis of *B. diazoefficiens* (Quelas et al., 2016a) and are not the only flagella responsible for swarming (Covelli et al., 2013). The only role that has been proposed for lateral flagella thus far is their interaction with subpolar flagella for swimming near surfaces or in viscous media (Quelas et al., 2016a), but these extremely specialized attributes seem not to constitute an obvious advantage or one teleologically sufficient to justify the high energy expenditure observed here macroscopically. From these energetic and ecologic points of view, the expression of lateral flagella in liquid medium, a property that *B. diazoefficiens* shares with only *Shewanella putrefaciens* (Bubendorfer et al., 2014; Quelas et al., 2016a) might be regarded as a parasitic trait instead of an advantageous property, a conclusion supported by the high competitiveness of the *lafR*::Km mutant against the wild-type under conditions permissive for lateral flagella expression in liquid medium. Since evidence for the horizontal acquisition of the lateral flagellar gene cluster has not yet been found in *B. diazoefficiens* (Liu and Ochman, 2007; Quelas et al., 2016a) the present observations indicate the need to perform further in-depth studies on the evolutionary relationships of these genes within the genus *Bradyrhizobium*, as well as to deduce which selection pressure has favored either the presence or the absence of lateral flagella in the different *Bradyrhizobium* lineages.

## Conclusion

This research has pointed out certain advantages of Mtl for use as C-source in inoculant production—such as providing a higher biomass and higher EPS and PHB contents along with the possibility of CO_2_ fixation in the rhizosphere—whereas cultivation in Ara may lead to a waste of energy by the lateral flagellar system during growth, and whose role in the interaction of the bacteria with soybean roots still remains not well understood. Since the advantages/disadvantages of Mtl and Ara as a C-sources seem related to the catabolic pathways employed by the cells, our results may help in the consideration of other carbon sources of lower cost according if they are catabolized by one or another pathway.

## Author Contributions

CC, JP-G, CR, and ML: performed the experiments, analyzed the data, and revised the work critically. AL: conceived the project, analyzed the data, revised the work critically, wrote the article, and obtained funding.

## Conflict of Interest Statement

The authors declare that the research was conducted in the absence of any commercial or financial relationships that could be construed as a potential conflict of interest.

## Footnotes

1 http://genome.annotation.jp/rhizobase/Bradyrhizobium

2 http://www.uniprot.org/

3 http://www.genome.jp/kegg/genes.html

4 http://www.microbesonline.org/

5 http://www.microbesonline.org/cgi-bin/vertiGo.cgi?term=18810&taxId=224911&goBrowse=0

## Abbreviations

*μ*_max_: Specific growth rate
6PG: D-gluconate-6-phosphate
Ara: L-arabinose
AraDP: L-arabinose-differential pool of proteins
CBB: Calvin–Benson–Bassham pathway
EPS: extracellular polysaccharide
ETC: electron-transport chain
F6P: D-fructose-6-phosphate
GA3P: D-glyceraldehyde-3-phosphate
HMY: HM salts-yeast extract medium
Km: kanamycin
L-KDA: L-2-keto-3-deoxyarabonate
Mtl: D-mannitol
MtlDP: D-mannitol-differential pool of proteins
nLC: nano-liquid chromatography
OD_500nm_: Optical density at 500 nm
P-glycerate: Glycerate-3-phosphate
PEP: phosphoenolpyruvate
PHB: Poly-3-hydroxybutyrate
Pi: inorganic phosphate
PP: pentose-phosphates pathway
*qCO*_2max_: specific CO_2_-production rate
*qO*_2max_, Specific O_2_-consumption rate, *qS*_max_: Specific substrate-consumption rate
Ru5P: D-Ribulose-5-phosphate
SD: standard deviation
Sm: Streptomycin
TCA-cycle: Tricarboxylic acids cycle
THF: Tetrahydrofolate
X5P: D-xylulose-5-phosphate
*y*_ATP/S_: adenosine triphosphate production per C-mole of substrate
*y_x/ATP_*: biomass produced per mole of adenosine triphosphate
*y*_x/s_: Biomass yield per C-mole of substrate consumed.
