# Supplementary material for: Induction by Bradyrhizobium diazoefficiens of Different Pathways for Growth in D-mannitol or L-arabinose Leading to Pronounced Differences in CO2 Fixation, O2 Consumption, and Lateral-Flagellum Production
Source: Front Microbiol. 2018 Jun 5;9:1189. doi: 10.3389/fmicb.2018.01189 (PMC5996035; doi:10.3389/fmicb.2018.01189)
Supplement: Supplementary file 2 [file Data_Sheet_1.PDF]

INDUCTION BY *BRADYRHIZOBIUM DIAZOEFFICIENS* OF DIFFERENT PATHWAYS FOR GROWTH IN D-MANNITOL OR L-ARABINOSE LEADING TO PRONOUNCED DIFFERENCES IN CO_2 FIXATION, O_2 CONSUMPTION, AND LATERAL-FLAGELLUM PRODUCTION

Carolina Cogo[1,2], Julieta Pérez-Giménez[1], Chandrasekar Balu Rajeswari[1], María Flavia Luna[3], Aníbal Roberto Lodeiro[1,]*

[1]Instituto de Biotecnología y Biología Molecular (IBBM). Facultad de Ciencias Exactas-UNLP y CCT La Plata-CONICET

[2]Departamento de Ciencias Básicas, Facultad de Ingeniería-UNLP

[3]Centro de Investigación y Desarrollo en Fermentaciones Industriales (CINDEFI). Facultad de Ciencias Exactas-UNLP y CCT La Plata-CONICET; CIC-PBA

Calles 47 y 115 (1900) La Plata, Argentina

*Corresponding author. Tel/Fax: +54-221-422-9777. E-mail: lodeiro@biol.unlp.edu.ar

SUPPLEMENTARY MATERIAL

Including:

Figure S1: Examples showing the quality of protein samples used for separation and anlysis in the Orbitrap equipment.

Table S1 (in separate Excel file): All the proteins detected in our study, listed by Proteome Discoverer. Mtl-Memb: Membrane fraction from HMY-Mtl; Mtl-Cyto: Cytoplasm fraction from HMY-Mtl; Ara-Memb: Membrane fraction from HMY-Ara; Ara-Cyto: Cytoplasm fraction from HMY-Ara. The biological replica numbers (#) are indicated after these labels.

Table S2: The D-mannitol-differential pool (MtlDP).

Table S3: The L-arabinose-differential pool (AraDP).

Table S4: Summary of the proteins found in the MtlDP and AraDP classified according to functional annotations.

Table S5: Proteins with well-defined annotated functions from the differential pools, classified according to gene-onthology (GO) biological processes.

Figure S2: Mtl catabolism through the PP pathway.

Table S6: Stoichiometric balance of the predicted reactions for the catabolism of D-mannitol in the pentose-phosphate (PP) pathway.

Figure S3: Mtl catabolism through the CBB pathway with RuBisCO catalysis involving 100% oxygenase activity along with glyoxylate oxidation occurring through the oxalate pathway.

Table S7: Stoichiometric balance of the predicted reactions for the catabolism of D-mannitol under the condition of oxygenase as being the sole activity in the RuBisCO catalysis and with glyoxylate oxidation in the oxalate pathway.

Figure S4: Mtl catabolism through the CBB pathway with RuBisCO catalysis involving 100% oxygenase activity along with C-recycling though the THF cycle.

Table S8: Stoichiometric balance of the predicted reactions for the catabolism of D-mannitol under the condition of oxygenase as being the sole activity in the RuBisCO catalysis and with carbon recycling through the tetrahydrofolate (THF) and glyoxylate cycles.

Figure S5: Mtl catabolism through the CBB pathway with 50% oxygenase and 50% carboxylase activities in the RuBisCO catalysis and glyoxylate oxidation through the oxalate pathway.

Table S9: Stoichiometric balance of the predicted reactions for the catabolism of D-mannitol under the condition of 50% oxygenase and 50% carboxylase activities in the RuBisCO catalysis and with glyoxylate oxidation in the oxalate pathway.

Figure S6: Mtl catabolism through the CBB pathway with 50% oxygenase and 50% carboxylase activities in the RuBisCO catalysis and C-recycling though the THF cycle.

Table S10: Stoichiometric balance of the predicted reactions for the catabolism of D-mannitol under the condition of 50% oxygenase and 50% carboxylase activities in the RuBisCO catalysis and with carbon recycling through the tetrahydrofolate (THF) and glyoxylate cycles.

Figure S7: Mtl catabolism through the CBB pathway with carboxylase as the sole activity in the RuBisCO catalysis.

Table S11: Stoichiometric balance of the predicted reactions for the catabolism of D-mannitol under the condition of 100% carboxylase activity in the RuBisCO catalysis.

Figure S8: Ara catabolism through the L-KDA pathway.

Table S12: Stoichiometric balance of the predicted reactions for the catabolism of L-arabinose.

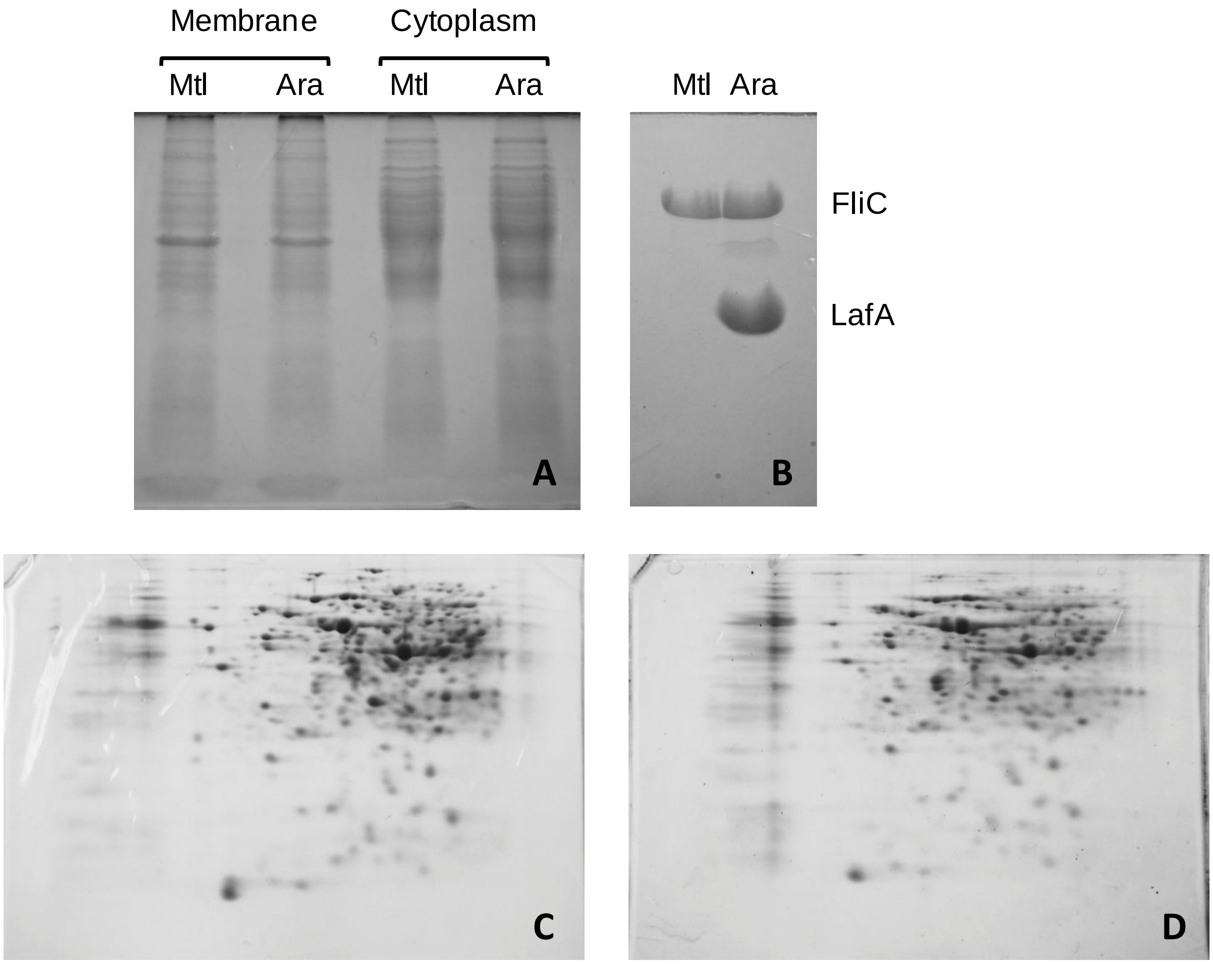

Fig. S1 Examples showing the quality of protein samples used for separation and anlysis in the Orbitrap equipment. A: One-dimensional SDS-PAGE of total proteins from the indicated fractions. B: One-dimensional SDS-PAGE of extracellular proteins, where the subpolar flagellins (FliC) and lateral fagellins (LafA), which were identified previously (ref. 23), are clearly visible. C-D: Two-dimensional SDS-PAGE (pI range: 4-7 in the horizontal dimension; MW in the vertical dimension) of samples from the Cytoplasm fractions of cells cultured in Mtl (C) or Ara (D). The protein extracts (A-D) are from sample #3. All other protein extracts mentioned in Table 1 produced similar results (not shown).

Table S2. The D-mannitol-differential pool (MtlDP) is composed of the proteins identified in the cytoplasmic-enriched fraction (Cytoplasm column) or in the membrane-enriched fraction (Membrane column) that were significantly more abundant in the Mtl-grown than in the Ara-grown cultures, as indicated by the Student-t-test. The $\log_2$ fold-change values of the Mtl/Ara ratio of the areas under the chromatogram peaks were considered significant when they were >1 (at least 2-fold upregulated in HMY-Mtl), with a Student-t-test p <0.05. In addition, those proteins identified in only Mtl are indicated. Otherwise, certain proteins were differentially expressed in one fraction, but appeared with no significant difference (NS) in the other. Only proteins identified in at least two biologic replicas are included. Gene tags circumscribed by squares or rectangles in the first column indicate those loci that were predicted as lying in the same operon by the MicrobesOnline server. The descriptions are based on data from Rhizobase, Uniprot, KEGG, and our previous work. The dashes indicate that the protein in question is absent in the fraction.

**Gene tag (Rhizobase)**	**Protein ID (Uniprot)**	**Gene name**	**Description**	**Fold-change ($\log_2$ Mtl area/Ara area)**	
				**Cytoplasm**	**Membrane**
blr0103	Q89Y51		Probable oxidoreductase	1.1521	—
blr0106	Q89Y49		Probable monooxygenase.	In only Mtl	1.0715
bll0128	Q89Y27		Hypothetical protein.	1.6187	In only Mtl
blr0135	Q89Y20		Hypothetical protein.	2.3932	NS
blr0172	Q89XY3		Hypothetical protein.	In only Mtl	NS
bll0187	Q89XW8	*aroB*	3-dehydroquinate synthase.	1.0842	—
bll0196	Q89XV9		ABC transporter substrate-binding protein.	In only Mtl	—
bll0198	Q89XV7		Amidase.	1.7150	—
bll0214	Q89XU2	*ubiG*	Ubiquinone biosynthesis O-methyltransferase.	In only Mtl	NS
bll0225	Q89XT2	*phaB*	Acetoacetyl CoA reductase.	2.7381	NS
blr0227	Q89XT0	*phaR*	Transcriptional regulator of polyhydroxybutirate synthesis.	1.6828	NS
bll0313	Q89XJ8		Hypothetical protein. Oxidoreductase activity.	In only Mtl	—
blr0337	Q89XH7		Putative carbon monoxide dehydrogenase medium chain.	1.1132	NS
bll0339	Q89XH5		4-hydroxyphenylpyruvate dioxygenase.	In only Mtl	—
bll0380	Q89XD4		ABC transporter ATP-binding protein.	—	In only Mtl
blr0418	Q89X96		Putative epoxide hydrolase.	In only Mtl	—
blr0518	Q89X09		Unknown protein.	In only Mtl	NS
bll0590	Q89WT8		Putative cytochrome C4 precursor.	In only Mtl	—
bll0598	Q89WT0		Hypothetical protein.	2.9749	NS
bll0605	Q89WS3		Unknown protein.	In only Mtl	NS
blr0614	Q89WR4		Hypothetical protein. Carbon-sulfur lyase activity.	In only Mtl	—
bll0679	Q89WK3		Hypothetical protein.	1.0977	—
bll0729	Q89WG1	*hspH*	Small heat shock protein.	In only Mtl	NS
bll0754	Q89WD6	*galF*	Nucleotidyltransferase family protein.	In only Mtl	NS
bll0792	Q89WA0		Magnesium and cobalt efflux protein.	In only Mtl	NS
bll0805	Q89W88		Hypothetical protein.	3.2930	—
bll0912	Q89VY3		Hypothetical protein.	1.5025	—
bll0913	Q89VY2		Hypothetical protein.	2.2672	—
blr0928	Q89VW7	*pcaG*	Protocatechuate 3.4-dioxygenase alpha chain.	1.2766	—
bll0954	Q89VU1		Hypothetical protein.	1.3589	—
blr0978	Q89VR7		Unknown protein.	In only Mtl	NS

blr0986	Q89LX3	*mccB*	3-methylcrotonoyl-CoA carboxylase beta subunit.	3.3406	—
bll0989	Q89VQ6		Peptide ABC transporter ATP-binding protein.	—	In only Mtl
bll0994	Q89VQ1		Probable acid-CoA ligase.	In only Mtl	NS
blr1003	Q89VP2		Hypothetical protein.	—	In only Mtl
bll1007	Q89VN8		Hypothetical protein.	In only Mtl	—
blr1046	Q89VJ9		Long-chain-fatty-acid-CoA ligase.	—	In only Mtl
bll1054	Q89VJ1		ABC transporter ATP-binding protein.	—	In only Mtl
bll1058	Q89VI7		ABC transporter substrate-binding protein.	2.6138	—
blr1062	Q89VI3	*bjaR1*	Quorum sensing transcriptional activator protein.	—	In only Mtl
blr1094	Q89VF2	*pstB*	Phosphate import ATP-binding protein.	—	1.1543
blr1123	Q89VC4		ABC transporter sugar-binding protein.	1.3196	—
blr1152	Q89V95		Unknown protein.	In only Mtl	—
bll1162	Q89V85	*attY*	Glutathione S-transferase.	1.0246	NS
blr1170	H7C6E5	*coxB*	Cytochrome c oxidase subunit 2.	1.3407	—
blr1249	Q89V09		Hypothetical protein.	1.1962	—
blr1279	Q89UY0		Transcriptional regulatory protein GntR family.	In only Mtl	NS
blr1309	Q89UV0	*acs*	Acetyl-coenzyme A synthetase.	NS	In only Mtl
blr1327	H7C6H7		Hypothetical protein. 2-nitropropane dioxygenase activity.	NS	In only Mtl
bll1406	Q89UL0		Hypothetical protein.	1.9458	—
bll1419	Q89UJ7	*metF*	Methylenetetrahydrofolate reductase.	In only Mtl	NS
bll1438	Q89UH9	*ctpE*	Pilus assembly protein.	—	In only Mtl
blr1454	Q89UG3	*ureA*	Urease subunit gamma.	—	In only Mtl
blr1496	Q89UC1		Unknown protein. UPF0337	—	In only Mtl
blr1506	Q89UB1		Hypothetical protein.	—	In only Mtl
blr1508	Q89UA9		HlyB/MsbA family ABC transporter.	In only Mtl	NS
bll1511	Q89UA6		Hypothetical protein.	In only Mtl	NS
blr1515	Q89UA2	*acrA*	RND multidrug efflux membrane permease.	In only Mtl	NS
bll1521	Q89U96		Putative fructose-1.6-bisphosphate aldolase protein.	1.9439	NS
blr1564	Q89U55		Unknown protein	—	In only Mtl
bll1569	Q89U50		Unknown protein.	—	In only Mtl
blr1571	Q89U48		Unknown protein.	In only Mtl	NS
blr1889	H7C811		ABC transporter substrate-binding protein.	In only Mtl	—
blr2097	H7C6S8	*bioF*	8-amino-7-oxononanoate synthase.	—	In only Mtl
blr2098	Q9AMS4	*bioD*	ATP-dependent dethiobiotin synthetase.	In only Mtl	—
blr2099	H7C6S2	*bioA*	Adenosylmethionine-8-amino-7-oxononanoate aminotransferase.	In only Mtl	—
blr2167	Q89T83		Oxidoreductase. Similar to 3-oxoacyl-[acyl-carrier-protein] reductase.	In only Mtl	In only Mtl
blr2168	Q89T82		Putative transketolase alpha subunit protein.	In only Mtl	—
blr2169	Q89T81		Putative transketolase beta subunit protein.	In only Mtl	In only Mtl
blr2170	Q89T80		Probable ABC transporter substrate-binding protein.	In only Mtl	—
blr2194	Q89T56	*cheY*	Chemotaxis two-component response regulator.	2.3829	—
bll2211	Q89T39	*copB*	Copper tolerance protein.	—	In only Mtl
blr2221	Q89T29	*bioA*	Adenosylmethionine-8-amino-7-oxononanoate aminotransferase.	1.1619	—

blr2248	Q89T02		Hypothetical protein. Similar to oxygen-independent coproporphyrinogen III oxidase.	In only Mtl	NS
blr2279	Q89SX1		Hypothetical protein. Phosphogluconate dehydrogenase (decarboxylating) activity.	—	In only Mtl
blr2290	Q89SW0		Unknown protein. Similar to isocitrate lyase.	—	1.6801
bll2291	Q89SV9		Unknown protein.	2.5341	NS
bll2304	Q89SU6		Probable ABC transporter.	2.4744	—
blr2397	Q89SK3		GMC type oxidoreductase. Choline dehydrogenase.	In only Mtl	NS
blr2438	Q89SG2		ABC transporter ATP-binding protein.	In only Mtl	NS
bll2465	Q89SD5		MoxR family protein.	1.0860	2.7405
bll2494	Q89SB1		Hypothetical protein. Similar to LemA protein.	In only Mtl	NS
bll2565	Q89S40		Hypothetical protein.	1.0905	—
blr2581	Q8GKS1	*fbp*	Fructose-1.6-bisphosphatase class 1.	In only Mtl	—
blr2582	H7C6I1	*cbbP*	Phosphoribulokinase.	In only Mtl	—
blr2583	H7C6U6	*cbbT*	Transketolase.	In only Mtl	In only Mtl
blr2584	H7C7W4	*cbbA*	Fructose-bisphosphate aldolase.	In only Mtl	In only Mtl
blr2585	Q9ZI34	*cbbL*	Ribulose bisphosphate carboxylase large chain.	In only Mtl	In only Mtl
blr2586	Q9ZI33	*cbbS*	Ribulose bisphosphate carboxylase small chain.	In only Mtl	In only Mtl
blr2587	Q89S25	*cbbX*	Protein CbbX of unknown function	—	In only Mtl
blr2588	Q89S24	*cbbE*	Ribulose-phosphate 3-epimerase.	In only Mtl	—
bll2624	Q89RY8	*pdxH*	Pyridoxine/pyridoxamine 5'-phosphate oxidase.	NS	In only Mtl
bll2655	Q89RV7		Putative enoyl-CoA hydratase.	In only Mtl	NS
bll2677	Q89RT5		Sugar ABC transporter ATP-binding protein.	—	In only Mtl
blr2739	Q89RM4		Hypothetical methyltransferase. 4 iron. 4 sulfur cluster binding.	In only Mtl	NS
blr2749	Q89RL4		Probable C4-dicarboxylate-binding protein.	1.0492	—
blr2762	H7C814		Hypothetical protein.	In only Mtl	NS
bll2784	Q89RI8		Hypothetical protein.	In only Mtl	—
blr2815	Q89RF7		Putative transketolase family protein. Pyruvate dehydrogenase E1 component.	In only Mtl	In only Mtl
blr2820	Q89RF2		Unknown protein.	1.2574	—
blr2837	Q89RD5		Hypothetical protein. Thiamine biosynthetic process.	In only Mtl	—
blr2840	Q89RD2		Oxidoreductase.	1.3541	—
blr2881	Q89R91	*ephA*	Epoxide hydrolase.	NS	In only Mtl
blr2885	Q89R87	*phaC4*	Probable poly-beta-hydroxybutyrate synthase.	—	In only Mtl
blr2891	Q89R81		Putative phenylacetic acid degradation protein.	—	In only Mtl
blr2895	Q89R77	*paaE*	Putative ferredoxin reductase electron transfer component protein.	In only Mtl	—
blr2897	Q89R75	*paaK*	Phenylacetate-coenzyme A ligase.	In only Mtl	NS
bll2905	Q89R67		Probable cytochrome P450.	In only Mtl	—
blr2912	Q89R60		Probable ABC transporter permease protein.	—	In only Mtl
blr2921	Q89R51		Hypothetical protein. UPF0317 protein.	—	In only Mtl
blr2978	Q89QZ4		Hypothetical protein. Similar to short-chain type dehydrogenase/reductase.	1.7474	—
blr2981	Q89QZ1		Hypothetical protein.	—	In only Mtl
bll3051	Q89QS3		Hypothetical protein.	1.2752	—
bll3180	Q89QE8		Probable 4-hydroxyacetophenone monooxygenase.	In only Mtl	NS

blr3183	Q89QE5		ABC transporter substrate-binding protein.	3.8769	—
blr3186	Q89QE2		ABC transporter ATP-binding protein.	—	In only Mtl
blr3187	Q89QE1		ABC transporter ATP-binding protein.	—	In only Mtl
blr3218	Q89QB2		Putative hydrolase phosphatase protein (rhizobase).	In only Mtl	NS
blr3219	Q89QB1		Probable transcriptional regulator. Similar to sorbitol/mannitol operon transcriptional regulator protein (SmoC).	In only Mtl	NS
blr3220	Q89QB0		Hypothetical protein. Similar to periplasmic sorbitol-binding protein.	In only Mtl	In only Mtl
blr3221	Q89QA9		ABC transporter substrate-binding protein. Similar to sorbitol/mannitol ABC transporter. Substrate-binding protein.	In only Mtl	In only Mtl
blr3224	Q89QA6		ABC transporter ATP-binding protein. Putative sorbitol/mannitol transport system.	In only Mtl	NS
blr3225	Q89QA5		Oxidoreductase. Similar to putative Glucose/ribitol oxidoreductase.	5.0927	In only Mtl
blr3226	Q89QA4		Ribitol kinase.	3.9234	—
blr3227	Q89QA3		Putative fructokinase.	In only Mtl	—
bll3259	Q89Q71	*cobQ*	Cobyric acid synthase.	In only Mtl	—
blr3392	Q89PT8		Dehydrogenase.	In only Mtl	NS
bll3426	Q89PQ4		ABC transporter substrate-binding protein.	1.8376	—
blr3650	Q89P33		Probable polysaccharide biosynthesis protein.	In only Mtl	NS
blr3678	Q89P05		Putative oxidoreductase. 2 iron. 2 sulfur cluster binding; electron carrier activity; FAD binding.	—	In only Mtl
blr3683	Q89P00	*groL4*	60 kDa chaperonin 4.	—	In only Mtl
blr3745	Q89NT9		Periplasmic mannitol-binding protein.	In only Mtl	—
blr3747	Q89NT7		Two-component response regulator.	—	In only Mtl
bll3754	Q89NT0	*gph*	Phosphoglycolate phosphatase.	In only Mtl	NS
blr3796	Q89NP2	*psd*	Phosphatidylserine decarboxylase proenzyme.	In only Mtl	NS
bll3856	Q89NI2		Probable medium-chain-fatty-acid-CoA ligase.	In only Mtl	NS
blr3887	Q89NF3	*pdxA1*	4-hydroxythreonine-4-phosphate dehydrogenase 1. Vitamin B6 metabolism.	In only Mtl	NS
blr3904	Q89ND8		Probable iron transport protein.	—	In only Mtl
blr3920	Q89NC2		ABC transporter permease protein.	In only Mtl	NS
bll3937	Q89NA5		Hypothetical protein.	3.3634	—
blr3940	Q89NA2		Putative propionyl-CoA carboxylase beta chain.	—	In only Mtl
bll3950	Q89N92		Putative enoyl-CoA hydratase.	In only Mtl	NS
blr3973	Q89N69		Probable NADP-dependent oxidoreductase.Similar to quinone oxidoreductase.	NS	2.0194
blr4037	Q89N05		ABC transporter ATP-binding protein.	In only Mtl	NS
bll4057	Q89MY5		Probable substrate-binding protein.	3.1767	—
bsl4127	Q89MR5		Cold shock protein.	1.0812	—
blr4219	Q89MH4		Hypothetical protein.	In only Mtl	NS
blr4240	Q89MF3		Hypothetical protein. FMN binding; pyridoxamine-phosphate oxidase activity.	In only Mtl	—
blr4245	Q89ME8		Unknown protein.	—	In only Mtl
bll4360	Q89M33	*phaC1*	Poly-3-hydroxybutyrate synthase.	2.7271	NS
bll4367	Q89M26		Hypothetical protein.	1.9503	NS
blr4371	Q89M22		Hypothetical protein.	1.5186	—
blr4419	Q89LX4	*acd*	Acyl-CoA dehydrogenase.	In only Mtl	NS
blr4421	Q89LX2	*mccA*	3-methylcrotonyl-CoA carboxylase alpha subunit.	1.8058	1.9852

blr4446	Q89LU7		ABC transporter amino acid-binding protein.	NS	In only Mtl
blr4449	Q89LU4		Amino acid ABC transporter ATP-binding protein.	In only Mtl	NS
bll4479	Q89LR4		Probable cytochrome C4.	—	In only Mtl
blr4489	Q89LQ4	*ntrY*	Two-component sensor histidine kinase.	In only Mtl	NS
blr4553	Q89LJ1		ABC transporter substrate-binding protein.	3.7020	—
blr4557	Q89LI7		ABC transporter ATP-binding protein. Similar to sn-glycerol 3-phosphate transport protein.	In only Mtl	NS
bll4560	Q89LI4		Hypothetical protein.	In only Mtl	NS
bll4608	Q89LD7	*glmU*	Bifunctional UDP-N-acetylglucosamine pyrophosphorylase and Glucosamine-1-phosphate N-acetyltransferase.	NS	In only Mtl
bll4634	Q89LB2		Hypothetical protein.	—	In only Mtl
blr4646	Q89LA1		Hypothetical protein. Arabinose-5-phosphate isomerase activity; keto-3-deoxy-D-manno-octulosonic acid biosynthetic process.	In only Mtl	NS
blr4699	Q89L49		Putative outer-membrane protein precursor.	—	In only Mtl
blr4701	Q89L47		Putative outer-membrane immunogenic protein precursor.	In only Mtl	NS
bll4707	Q89L41		Hypothetical protein.	1.9525	—
blr4723	Q89L25		Hypothetical protein. Similar to cytochrome c oxidase subunit I.	—	In only Mtl
bll4755	Q89KZ3		Hypothetical protein.	—	In only Mtl
blr4842	Q89KR2		ABC transporter substrate-binding protein.	4.4799	—
blr4845	Q89KQ9		ABC transporter ATP-binding protein. Similar to sn-glycerol 3-phosphate transport protein.	—	In only Mtl
blr4846	Q89KQ8		Putative oxidoreductase protein.	In only Mtl	In only Mtl
bll4873	Q89KN2		Hypothetical protein. Signal transducer activity.	—	In only Mtl
blr4884	Q89KM1		ABC transporter substrate-binding protein.	2.1980	In only Mtl
bll4885	Q89KM0		ABC transporter ATP-binding protein. Ribose transport system.	—	In only Mtl
bll4950	Q89KF7		Probable L-asparaginase.	1.3799	NS
bll4983	Q89KC4		Hypothetical protein.	NS	1.4482
blr4994	Q89KB3		Hypothetical protein.	—	2.5087
bll5004	Q89KA5		Unknown protein.	—	1.3950
bll5012	Q89K97		Putative type I restriction enzyme.	In only Mtl	NS
bll5026	Q89K83	*hppA*	K(+)-insensitive pyrophosphate-energized proton pump.	NS	2.0436
bll5154	Q89JW5	*clpS2*	ATP-dependent Clp protease adapter protein.	1.1206	NS
blr5308	Q89JH5		Anti-oxidant protein. Similar to Thioredoxin peroxidase.	1.3576	1.4189
bll5338	Q89JE5		Probable oxidoreductase. Similar to succinate-semialdehyde dehydrogenase.	In only Mtl	NS
blr5345	Q89JD8		Transcriptional regulatory protein.	In only Mtl	—
blr5359	Q89JC4		Unknown protein. Hydrolase activity.	In only Mtl	NS
bll5400	Q89J84	*rplC*	50S ribosomal protein L3.	1.3615	NS
blr5422	Q89J62		Hypothetical protein. Similar to bifunctional protein HldE.	1.7226	NS
blr5423	Q89J61		Probable dTDP-glucose-4.6 dehydratase.	1.0068	NS
blr5429	Q89J55		Hypothetical protein.	NS	In only Mtl
bll5494	Q89IZ0		Hypothetical protein.	4.1730	—
bll5495	Q89IY9		Hypothetical protein.	In only Mtl	—
bll5496	Q89IY8		Hypothetical metabolite transport protein.	—	In only Mtl
bll5697	Q89IE2		Putative long-chain-fatty-acid--CoA ligase.	1.8982	—

bll5745	Q89I94		Unknown protein.	—	In only Mtl
blr5765	Q89I74		Unknown protein.	NS	In only Mtl
bll5892	Q89HU7		Hypothetical protein.	—	1.8055
bll5943	Q89HP7		Unknown protein.	In only Mtl	—
blr5966	Q89HM4		ABC transporter substrate-binding protein.	2.5811	—
blr6000	Q89HJ1		Unknown protein.	In only Mtl	NS
blr6085	Q89HA9		Putative acid-CoA ligase.	—	In only Mtl
bll6129	Q89H66		Hypothetical protein.	2.2266	1.3807
blr6135	Q89H60		Unknown protein.	1.7699	NS
blr6158	Q89H37		ABC transporter substrate-binding protein.	1.0701	—
bll6159	Q89H36		Putative hydroxydechloroatrazine ethylaminohydrolase.	In only Mtl	—
blr6190	Q89H05		ABC transporter ATP-binding protein.	NS	1.0870
bll6196	Q89H00		Putative methanol oxidation protein.	In only Mtl	—
bll6221	Q89GX4		Rieske iron-sulfur protein; components of cytochrome bc1 complexes and cytochrome b6f complexes.	1.5177	—
blr6230	Q89GW5		Putative beta-lactamase precursor.	1.9939	—
bll6350	Q89GJ5		Similar to tagatose-1.6-diphosphate aldolase.	In only Mtl	NS
blr6465	Q89G80		Putative steroid monooxygenase.	2.3132	NS
bll6479	Q89G66		ABC transporter substrate-binding protein.	In only Mtl	—
bll6500	Q89G47		Unknown protein. S-adenosylmethionine-dependent methyltransferase activity.	—	In only Mtl
bll6549	Q89FZ9		Hypothetical protein. NAD binding. phosphoglycerate dehydrogenase activity.	In only Mtl	—
bll6551	Q89FZ7		Probable ABC transporter substrate-binding protein.	In only Mtl	—
bll6583	Q89FW6		Unknown protein.	In only Mtl	—
bll6613	Q89FT6		Hypothetical protein.	In only Mtl	—
bll6688	Q89FL1		Hypothetical protein.	In only Mtl	—
bll6704	Q89FJ6		Two-component sensor activity with GGDEF and PAS domains.	—	In only Mtl
blr6726	Q89FH4		AcrB/AcrD/AcrF family protein.	—	In only Mtl
bll6755	P59348		Hypothetical proteinl. UPF0229 protein.	In only Mtl	NS
blr6763	Q89FD8		Hypothetical protein.	1.3939	NS
blr6770	Q89FD1		Malto-oligosyltrehalose trehalohydrolase.	In only Mtl	—
bll6803	Q89F98		Hypothetical metabolite transport protein.	In only Mtl	NS
bll6830	Q89F71	*uxuA*	Mannonate dehydratase.	2.2793	—
bll6834	Q89F67		Probable ABC transporter substrate-binding protein.	1.2258	—
bll6888	Q89F13		Putative porin.	NS	1.3280
blr7061	Q89EL2		Hypothetical protein.	1.5061	—
blr7063	Q89EL0		Probable 2-ketogluconate reductase.	2.5998	—
blr7064	Q89EK9		Probable ABC transporter substrate-binding protein.	In only Mtl	—
bll7086	O31381	*hemN*	Oxygen-independent coproporphyrinogen-III oxidase.	In only Mtl	NS
blr7099	Q89EI6		Probable ABC transporter substrate-binding protein.	1.1252	In only Mtl
blr7131	Q89EF4		Hypothetical protein.	In only Mtl	2.0018
bll7139	Q89EE6		Transcriptional regulatory protein TetR family.	In only Mtl	NS
bll7168	Q89EB9		Hypothetical protein. Similar to glyoxalase family proteins.	In only Mtl	—
blr7242	Q89E45		Putative cytochrome P450.	2.7502	NS

blr7270	Q89E17		Probable acyl-CoA dehydrogenase.	In only Mtl	—
bll7414	Q89DM5		Probable Elongation factor G.	In only Mtl	NS
blr7460	Q89DH9		Transcriptional regulatory protein MarR family.	1.6117	—
bsr7468	Q89DH1	*cspA*	Cold shock protein.	In only Mtl	—
blr7499	Q89DE0		Unknown protein.	In only Mtl	NS
bll7531	Q89DA8		Hypothetical protein.	NS	1.2291
blr7589	Q89D54		Putative oxidoreductase.	—	In only Mtl
bll7689	Q89CV6		Putative monooxygenase.	—	In only Mtl
blr7695	Q89CV0		Putative outer-membrane immunogenic protein precursor.	—	1.6456
blr7746	Q89CQ0		Capsule expression protein, arabinose-5-phosphate isomerase.	In only Mtl	NS
bll7800	Q89CJ7		Putative acyl-CoA dehydrogenase.	1.9001	—
bll7801	Q89CJ6		Putative dehydrogenase.	1.4099	—
blr7806	Q89CJ1		Putative beta-ketoadipyl CoA thiolase.	1.1307	NS
blr7814	Q89CI3		Putative L-proline 4-hydroxylase.	In only Mtl	—
blr7816	Q89CI1		Probable mannitol-binding protein. Part of the tripartite ATP-independent periplasmic (TRAP) transport system.	In only Mtl	—
bll7820	Q89CH7		Putative long-chain-fatty-acid--CoA ligase.	1.2017	—
blr7823	Q89CH4		ABC transporter ATP-binding protein.	In only Mtl	NS
blr7824	Q89CH3		ABC transporter ATP-binding protein.	In only Mtl	NS
blr7829	Q89CG8		Hypothetical protein.	In only Mtl	—
blr7839	Q89CF8		Probable substrate-binding protein.	2.0919	—
blr7869	Q89CC8		ABC transporter permease protein.	—	In only Mtl
blr7890	Q89CA7		Putative beta-lactamase.	NS	In only Mtl
bll7898	Q89C99		Alcohol dehydrogenase.	3.4564	NS
bll7908	Q89C89		Hypothetical protein. Carboxy-lyase activity; hydrolase activity.	In only Mtl	—
bll7927	Q89C70		Hypothetical protein. Amino acid transport.	In only Mtl	—
bll7929	Q89C68		ABC transporter ATP-binding protein.	—	In only Mtl
bll7930	Q89C67		ABC transporter ATP-binding protein	—	In only Mtl
bll7962	Q89C36		Hypothetical protein.	2.2524	NS
blr7995	Q89C03		Hypothetical protein. Glutamate dehydrogenase (NAD^+) activity.	—	In only Mtl
bll8116	Q89BN2		DNA polymerase III tau subunit.	In only Mtl	NS

Table S3. The L-arabinose-differential pool (AraDP) is composed of the proteins identified in the cytoplasmic-enriched fraction (Cytoplasm column) or in the membrane-enriched fraction (Membrane column) that were significantly more abundant in the Ara-grown than in the Mtl-grown cultures, as indicated by the Student-t-test. The log_2 fold-change values of the Mtl/Ara ratio of the areas under the chromatogram peaks were considered significant when they were <1 (at least 2-fold upregulated in HMY-Ara), with a Student t test p <0.05. In addition, those proteins identified in only Ara are indicated. Otherwise, certain proteins were differentially expressed in one fraction, but appeared with no significant difference (NS) in the other. Only proteins identified in at least two biologic replicas are included. Gene tags circumscribed by squares or rectangles in the first column indicate those loci that were predicted as lying in the same operon by the MicrobesOnline server. The descriptions are based on data from Rhizobase, Uniprot, KEGG, and our previous work. The dashes indicate that the protein in question is absent in the fraction.

Gene tag (Rhizobase)	Protein ID (Uniprot)	Gene name	Description	Fold-change (log_2 Mtl area/Ara area) Cytoplasm	Membrane
bll0087	Q89Y68		Hypothetical protein.	-2.4862	—
bll0161	Q89XZ4		Hypothetical protein.	In only Ara	—
blr0252	Q89XQ6		Transcriptional regulatory protein.	—	In only Ara
blr0258	Q89XQ0		Two-component response regulator LuxR family.	In only Ara	—
blr0369	Q89XE5		Hypothetical protein. Metal ion transmembrane transporter activity.	NS	In only Ara
blr0372	Q89XE2		Putative succinoglycan biosynthesis protein.	In only Ara	—
blr0373	Q89XE1		Hypothetical aminotransferase.	-1.2957	NS
blr0424	Q89X90		Similar to monoamine oxidase regulatory protein.	In only Ara	—
blr0430	Q89X84	*nadD*	Probable nicotinate-nucleotide adenylyltransferase.	-1.3324	—
blr0462	Q89X53		Probable ABC transporter permease protein.	—	In only Ara
blr0495	Q89X27	*leuD*	3-isopropylmalate dehydratase small subunit.	-1.2598	—
blr0496	Q89X26		Hypothetical protein.	NS	-1.8490
bll0554	Q89WX4		Short-chain dehydrogenase.	—	-1.3643
bll0557	Q89WX1		Putative cytochrome P450.	—	-1.1230
blr0595	Q89FP4		Hypothetical protein.	In only Ara	In only Ara
blr0608	Q89WS0		Similar to ammonium transporter.	—	In only Ara
bll0664	Q89WL4	*ctpF*	Pilus CtpF protein.	—	-1.9284
blr0723	P30333	*rpoN2*	RNA polymerase sigma-54 factor 2.	NS	In only Ara
bll0761	Q89WD0	*ubiE*	Ubiquinone/menaquinone biosynthesis C-methyltransferase.	NS	In only Ara
blr0765	Q89WC6	*moeB*	Molybdopterin biosynthesis protein.	In only Ara	NS
blr0770	Q89WC2	*fabB*	3-oxoacyl-[acyl-carrier-protein] synthase I.	NS	In only Ara
bll0775	Q89WB7		Unknown protein.	In only Ara	NS
blr0817	Q89W76		Hypothetical protein.	—	In only Ara
bll0837	Q89W56		Carboxymethylenebutenolidase.	-1.3128	—
bll0886	Q89W07		ABC transporter ATP-binding protein.	—	In only Ara
bll0904	Q89VZ0	*regR*	Two-component response regulator.	NS	In only Ara
blr0908	Q89VY7		Hypothetical protein. Similar to PHB depolymerase.	In only Ara	-2.0085
blr0920	Q89VX5		Hypothetical protein.	-1.1171	NS
blr0936	Q89VV9	*ispG*	4-hydroxy-3-methylbut-2-en-1-yl diphosphate synthase (flavodoxin).	NS	-1.2799
bll0958	Q89VT7	*acd*	Acyl-CoA dehydrogenase.	NS	-1.0777
blr1100	Q89VE7	*hslO*	60 kDa chaperonin.	-1.2379	—

bll1188	Q89V68	*atpB*	ATP synthase subunit a.	—	In only Ara
blr1194	Q89V62	*tcsR*	Two-component response regulator.	NS	In only Ara
bll1195	Q89V61	*TtrR*	Transcriptional regulator.	—	In only Ara
bll1209	Q89V49		ABC transporter ATP-binding protein.	—	In only Ara
blr1253	Q89V05		Dehydrogenase. 3-keto sterol reductase activity.	—	In only Ara
blr1282	Q89UX7		Hypothetical protein.	-2.7013	—
bll1295	Q89UW4		Probable oxidoreductase.	-2.8273	—
bll1368	Q89UP3		Hypothetical protein.	In only Ara	—
bll1394	Q89UM2	*chaC*	Gamma-glutamylcyclotransferase.	—	In only Ara
bll1396	Q89UM0	*tyrC*	Prephenate dehydrogenase.	-1.5604	—
blr1399	Q89UL7	*metX*	Homoserine O-acetyltransferase.	In only Ara	—
blr1400	Q89UL6		Hypothetical protein. S-adenosylmethionine-dependent methyltransferase activity.	—	In only Ara
bll1403	Q89UL3		Hypothetical protein.	NS	In only Ara
bll1436	Q89UI1	*ctpG*	Pilus assembly protein.	—	-1.3979
bsl1446	Q89UH1	*infA*	Translation initiation factor IF-1.	NS	-1.1922
blr1460	Q89UF7	*ureG*	Urease accessory protein.	—	In only Ara
bll1476	Q89UE1	*cysD*	Sulfate adenylate transferase subunit 2.	-2.6911	-1.9273
blr1485	Q89UD2	*cysA*	Sulfate/thiosulfate import ATP-binding protein.	—	-2.0541
blr1487	Q89UD0		Hypothetical protein. Hydrolase. Lipid metabolic process.	—	In only Ara
bll1530	Q89U87		Putative aldehyde dehydrogenase protein.	In only Ara	—
blr1536	Q89U81	*ruvA*	Holliday junction ATP-dependent DNA helicase.	In only Ara	—
bll2268	Q89SY2		Probable transcriptional regulator.	In only Ara	—
blr2269	Q89SY1		ABC transporter sugar-binding protein.	-4.1959	In only Ara
blr2270	Q89SY0		Sugar ABC transporter ATP-binding protein.	In only Ara	NS
blr2271	Q89SX9		Sugar ABC transporter permease protein.	—	In only Ara
blr2272	Q89SX8		Porin. Carbohydrate transport.	In only Ara	-4.4685
blr2316	Q89ST4		Probable NADH-ubiquinone oxidoreductase chain F.	In only Ara	—
blr2343	Q89SQ7	*cheA*	Chemotaxis two-component sensor histidine kinase.	NS	-1.8082
blr2344	Q89SQ6	*cheWII*	CheWII protein.	In only Ara	NS
blr2345	Q89SQ5	*mcpK*	Methyl-accepting chemotaxis protein.	NS	-1.8623
blr2346	Q89SQ4	*cheW*	CheW protein.	In only Ara	—
blr2347	Q89SQ3		Methyl-accepting chemotaxis protein.	NS	-1.5919
blr2349	Q89SQ1	*cheB*	Chemotaxis response regulator protein-glutamate methylesterase of group 3 operon.	—	In only Ara
blr2404	Q89SJ6	*ggt*	Gamma-glutamyltranspeptidase.	-1.5158	NS
blr2406	Q89SJ3		Hypothetical protein.	In only Ara	—
bll2445	Q89SF5		Oxidoreductase. Similar to N-ethylmaleimide reductase.	-2.1907	—
bll2491	Q89SB4		Hypothetical protein.	-1.6199	—
bll2508	Q89S97		Hypothetical glutathione S-transferase like protein.	—	In only Ara
blr2522	Q89S83		Hypothetical protein.	—	-1.2363
bll2527	Q89S78		Putative hydrolase.	In only Ara	—
bll2541	Q89S64	*nadB*	L-aspartate oxidase.	In only Ara	-1.7782

bll2567	Q89S38		Hypothetical protein.	In only Ara	—
bll2568	Q89S37		Probable acetyltransferase.	—	In only Ara
bll2570	Q89S35		Threonylcarbamoyl-AMP synthase.	NS	In only Ara
bll2618	Q89RZ4		Hypothetical protein.	—	In only Ara
bll2633	Q89RX9		Hypothetical protein. Oxidoreductase activity.	In only Ara	—
bll2698	Q89RR4		Hippurate hydrolase. Metallodipeptidase activity	In only Ara	In only Ara
blr2720	Q89RP3		Unknown protein.	In only Ara	—
bll2736	Q89RM7		Putative aldehyde dehydrogenase.	In only Ara	—
bll2786	Q89RI6		Transcriptional regulatory protein LysR family.	NS	In only Ara
blr2826	Q89RE6	*ilvD*	Dihydroxy-acid dehydratase.	-1.3772	—
bll2869	Q89RA3		ABC transporter ATP-binding protein.	—	In only Ara
bll2872	Q89RA0	*ggt*	Gamma-glutamyltranspeptidase.	-1.3128	—
bll2917	Q89R55		Hypothetical oxidoreductase.	-1.1978	—
blr2929	Q89R43		Hydroxypyruvate isomerase.	NS	In only Ara
blr2931	Q89R41		Putative methyl-accepting chemotaxis protein.	—	In only Ara
blr2934	Q89R38	*ragC*	Probable cation efflux protein.	—	In only Ara
bll2957	Q89R15		Dehydrogenase	NS	-1.2017
bll2958	Q89R14		Hypothetical protein.	-1.9345	—
blr2961	Q89R11		Hypothetical protein.	NS	-2.3424
blr2974	Q89QZ8		Dehydratase. Similar to a putative D-galactarate dehydratase.	-1.2323	—
blr2986	Q89QY6		Two-component hybrid sensor and regulator.	NS	In only Ara
bll3009	Q89QW5		Hypothetical protein.	In only Ara	—
bll3052	Q89QS2		Hypothetical protein.	In only Ara	—
bll3058	Q89QR6		7.8-dihydroneopterin aldolase.	-1.6112	—
blr3074	Q89QQ0		Hypothetical protein.	In only Ara	—
bll3100	Q89QM4		Hypothetical protein.	—	In only Ara
bll3101	Q89QM3		Hypothetical protein.	—	In only Ara
bll3140	Q89QI8		Two-component sensor histidine kinase.	—	In only Ara
bll3143	Q89QI5		Hypothetical protein.	In only Ara	NS
bll3156	Q89QH2	*frc*	Formyl-CoA:oxalate CoA-transferase.	-2.1294	In only Ara
bll3157	Q89QH1	*oxc*	Oxalyl-CoA decarboxylase.	-2.3829	NS
blr3159	Q89QG9		Hypothetical protein.	-2.5440	—
blr3166	Q89QG2	*gcl*	Glyoxylate carboligase.	In only Ara	In only Ara
blr3167	Q89QG1	*hyi*	Hydroxypyruvate isomerase.	In only Ara	In only Ara
blr3168	Q89QG0		Tartronate semialdehyde reductase.	In only Ara	In only Ara
blr3200	Q89QC8		ABC transporter sugar-binding protein.	-3.0198	—
blr3201	Q89QC7		Sugar ABC transporter ATP-binding protein.	—	In only Ara
blr3202	Q89QC6		Sugar ABC transporter permease protein.	—	In only Ara
blr3204	Q89QC4		Transcriptional regulatory protein GntR family.	In only Ara	In only Ara
blr3205	Q89QC3		Dehydrogenase. Similar to Galactose 1-dehydrogenase.	—	-3.3450
blr3206	Q89QC2		Aldose 1-epimerase.	-5.0604	—

blr3207	Q89QC1		L-arabinonolactonase.	-4.7706	In only Ara
blr3208	Q89QC0		ABC transporter sugar-binding protein.	-2.3733	In only Ara
blr3209	Q89QB9		Sugar ABC transporter ATP-binding protein. Similar to L-arabinose transporter ATP-binding protein.	NS	-3.2407
blr3210	Q89QB8		Sugar ABC transporter permease protein.	—	In only Ara
blr3258	Q89Q72	*cobC*	Cobalamin biosynthetic protein.	In only Ara	NS
bll3359	Q89PX1		Hypothetical protein.	-1.8352	—
bll3388	Q89PU2		3-oxoacyl-(Acyl carrier protein) reductase.	NS	In only Ara
blr3570	Q89PB2		ABC transporter ATP-binding protein.	—	In only Ara
blr3571	Q89PB1		ABC transporter ATP-binding protein.	—	In only Ara
blr3573	Q89PA9		Hypothetical protein. Similar to dihydroxyacetone kinase phosphotransfer protein.	—	In only Ara
bll3740	Q89NU4		Dehydrogenase.	NS	In only Ara
bll3847	Q89NJ1		Hypothetical protein.	In only Ara	—
bll3874	Q89NG6		Putative amidase.	In only Ara	—
bll3876	Q89NG4		Aldehyde dehydrogenase.	NS	In only Ara
blr3878	Q89NG2		Putative hydrolase.	In only Ara	—
bll3902	Q89NE0		AcrB/AcrD/AcrF family protein.	—	-2.1371
bll3903	Q89ND9		Putative multidrug resistance protein.	—	-1.8690
bll3912	Q89ND0		Hypothetical protein.	In only Ara	—
bll3913	Q89NC9	*fabG*	3-oxoacyl-(Acyl-carrier protein) reductase.	-1.2802	NS
bll3914	Q89NC8		Oxidoreductase.	-1.3388	—
bll3915	Q89NC7		Dihydroxy-acid dehydratase.	-1.1402	NS
blr3922	Q89NC0	*kdgK*	2-dehydro-3-deoxygluconokinase.	-1.2287	NS
bll3965	Q89N77		Probable ATP-binding protein.	In only Ara	—
blr4134	Q89MQ8		Aminotransferase.	NS	In only Ara
bll4147	Q89MP5		Hypothetical protein.	In only Ara	—
blr4169	P04772	*glnII*	Glutamine synthetase 2	In only Ara	—
bll4270	Q89MC3		Unknown protein.	—	In only Ara
bll4288	Q89MA5		Two-component response regulator.	In only Ara	—
blr4340	Q89M53		Probable ATP-binding protein. Similar to SufC. Related to ABC transporter associated with Fe-S cluster assembly.	-1.1168	NS
blr4341	Q89M52		Hypothetical protein. Iron-sulfur cluster assembly. Similar to sufD. Needed for Fe-S center production/stability.	NS	In only Ara
blr4342	Q89M51	*nifS*	Cysteine desulfurase.	-1.5001	—
bll4389	Q89M04	*aroG*	Phospho-2-dehydro-3-deoxyheptonate aldolase.	—	In only Ara
bll4498	Q89LP5	*nthA*	Nitrile hydratase subunit alpha.	-1.1707	NS
blr4512	Q89LN1		Probable ABC transporter permease protein.	—	In only Ara
bll4547	Q89LJ7	*nadE*	Glutamine-dependent NAD(+) synthetase.	-1.2616	—
bll4573	Q89LH1		Two-component response regulator.	NS	In only Ara
bll4605	Q89LD9		Dehydrogenase.	NS	-2.1456
bll4607	P59362	*glmS*	Glutamine-fructose-6-phosphate aminotransferase.	NS	-3.1722
blr4657	Q89L91		Beta-glucosidase.	In only Ara	NS
bll4710	Q89L38		Hypothetical protein.	In only Ara	—

bll4822	Q89KT1	*ctpA*	Carboxy-terminal protease.	-1.6259	—
bll4849	Q89KQ5	*lpxA*	Acyl-[acyl-carrier-protein]-UDP-N-acetylglucosamine O-acyltransferase.	NS	In only Ara
bll4859	Q89KP5	*pyrH*	Uridylate kinase.	NS	In only Ara
bll4879	Q89KM6		Hypothetical protein.	In only Ara	—
bll5013	Q89K96		Unknown protein.	NS	In only Ara
blr5140	Q89JX9		Hypothetical protein.	In only Ara	—
blr5149	Q89JX0		Oxidoreductase.	-1.7391	NS
blr5227	P77829	*groL1*	60 kDa chaperonin 1.	NS	-1.7380
bll5254	Q89JM9		Unknown protein.	—	In only Ara
blr5306	Q89JH7		Hypothetical protein.	NS	In only Ara
bll5331	Q89JF2		Hypothetical protein.	-1.1074	—
bll5335	Q89JE8		Putative thiolase/acetyl-CoA acetyltransferase.	-1.7792	—
blr5434	Q89J50		Putative D-mycarose 3-C-methyltransferase.	NS	In only Ara
bll5452	Q89J32		Unknown protein.	In only Ara	—
bll5458	Q89J26		Hypothetical protein.	NS	-2.1557
blr5498	Q89IY6		Putative oxidoreductase protein.	In only Ara	—
bll5586	Q89IP8	*cysE*	Serine acetyltransferase.	-1.2358	In only Ara
blr5600	Q89IN4		ABC transporter ATP-binding protein.	NS	-1.0696
blr5601	Q89IN3		Hypothetical Microcystinase C.	NS	-2.5464
blr5603	Q89IN1	*gatA*	Glutamyl-tRNA amidotransferase subunit A.	-1.4826	—
blr5604	Q89IN0		Probable enoyl-CoA hydratase.	-1.3571	—
blr5623	Q89IL1		TPR domain protein.	—	In only Ara
blr5626	Q89IK8	*groL6*	60 kDa chaperonin 6.	NS	-1.3507
bll5638	Q89IJ6		Hypothetical protein.	—	In only Ara
blr5641	Q89IJ3		Hypothetical protein.	NS	In only Ara
bll5643	Q89IJ1		Hypothetical protein.	-2.5556	—
bll5662	H7C7U6		Hypothetical protein CO dehydrogenase.	—	-1.2432
blr5705	Q89ID4		Hypothetical protein.	—	In only Ara
blr5712	Q89IC7		Hypothetical protein.	In only Ara	—
blr5713	Q89IC6		Hypothetical protein	—	In only Ara
bll5856	Q89HY3		Hypothetical protein.	—	-2.9199
bll5859	Q89HY0		Unknown protein.	—	In only Ara
blr5884	Q89HV5		Hypothetical protein.	In only Ara	—
bll5993	Q89HJ7		Putative acetyltransferase.	In only Ara	—
bll6073	Q89HC1	*phaC2*	Polyhydroxybutirate synthase PhaC2.	In only Ara	NS
blr6255	Q89GU0		Hypothetical protein.	—	In only Ara
bll6273	Q89GS2		Probable biotin sulfoxide reductase.	In only Ara	—
blr6331	Q89GL4	*bkdA1*	2-oxoisovalerate dehydrogenase alpha subunit.	In only Ara	In only Ara
blr6332	Q89GL3	*bkdA2*	2-oxoisovalerate dehydrogenase beta subunit.	In only Ara	—
blr6333	Q89GL2	*bkdB*	Lipoamide acyltransferase component of branched-chain alpha-keto acid dehydrogenase complex E2.	In only Ara	—
blr6445	Q89GA0		ABC transporter ATP-binding protein.	—	In only Ara
bll6511	Q89G36		Acetyltransferase	In only Ara	—

blr6616	Q89FT3		Transcriptional regulatory protein GntR family.	—	In only Ara
blr6651	Q89FP8		Two-component response regulator (OmpR family).	—	In only Ara
blr6655	Q89FP4	*thiO*	Thiamine biosynthesis oxidoreductase.	In only Ara	In only Ara
blr6659	Q89FP0	*thiC*	Phosphomethylpyrimidine synthase. Thiamine biosynthesis protein.	-2.2400	NS
bll6724	Q89FH6		Unknown protein.	In only Ara	—
bll6800	Q89FA1		Hypothetical protein. Similar to fumarylacetoacetate hydroxylase.	-1.0973	In only Ara
bll6813	Q89F88	*iivG*	Acetolactate synthase large subunit.	-1.4470	NS
bll6858	Q89F43	*flgE$_L$*	Lateral flagellar hook protein	In only Ara	—
bll6865	Q89F36	*lafA2*	Lateral flagellin 2	In only Ara	In only Ara
bll6866	Q89F35	*lafA1*	Lateral flagellin 1	In only Ara	In only Ara
bll6897	Q89F04		ABC transporter ATP-binding protein.	—	In only Ara
bll6899	Q89F02		ABC transporter substrate-binding protein.	-2.0092	—
bll6900	Q89F01	*ahpD*	Alkyl hydroperoxide reductase.	In only Ara	—
blr7006	Q89ER6		Probable carboxypeptidase G2.	-2.0576	—
bll7042	Q89EN1		Hypothetical protein.	—	In only Ara
blr7044	Q89W59	*msrB*	Peptide methionine sulfoxide reductase.	In only Ara	—
blr7089	Q89EJ6	*nirK*	Respiratory nitrite reductase.	NS	In only Ara
blr7114	Q89EH1	*purE*	N5-carboxyaminoimidazole ribonucleotide mutase.	In only Ara	—
bll7134	Q89EF1		Hypothetical protein.	—	In only Ara
bll7180	Q89EA7		Hydantoin utilization protein.	NS	-1.4952
blr7239	Q89E48	*upp*	Uracil phosphoribosyltransferase.	NS	In only Ara
bll7287	Q89E00	*dgoA*	2-dehydro-3-deoxyphosphogalactonate aldolase.	-1.4610	—
bll7303	Q89DY4		Hypothetical protein.	—	In only Ara
blr7444	Q89DJ5	*lgt*	Prolipoprotein diacylglyceryl transferase.	—	In only Ara
blr7446	Q89DJ3		Laccase domain protein.	—	-1.2660
blr7448	Q89DJ1	*prs*	Ribose-phosphate pyrophosphokinase.	NS	-1.4528
bll7452	Q89DI7	*ribH2*	6,7-dimethyl-8-ribityllumazine synthase 2.	In only Ara	—
blr7466	Q89DH3		Ribonuclease.	NS	In only Ara
blr7525	Q89DB4	*hisG*	ATP phosphoribosyltransferase.	-1.1863	—
blr7556	Q89D86		Non-heme haloperoxidase.	In only Ara	—
bll7572	Q89D70		Unknown protein.	—	In only Ara
blr7575	Q89D67	*metA*	O-methyl transferase.	NS	In only Ara
blr7604	Q89D42	*mdlC*	Probable benzoylformate decarboxylase.	-1.8798	—
bll7783	Q89CL4		Hypothetical protein.	In only Ara	NS
bll7798	Q89CJ9		Putative nitroreductase.	NS	In only Ara
blr7827	Q89CH0		ABC transporter substrate-binding protein.	NS	In only Ara
bll7877	Q89CC0		Unknown protein.	—	In only Ara
bll7969	Q89C29		Putative dihydrodipicolinate synthase.	-3.9249	In only Ara
blr7970	Q89C28		Oxidoreductase.	In only Ara	—
blr8103	Q89BP5		Hypothetical protein.	—	In only Ara
blr8123	Q89BM5		MutT/nudix family protein.	-1.3450	NS
blr8140	Q89BK8		Hypothetical protein.	—	-1.2387

Table S4. Summary of the proteins found in the MtlDP and AraDP classified according to functional annotations.

	**MtlDP**		**AraDP**	
	Quantity	%	Quantity	%
Hypothetical	67	25.2	62	26.2
Unknown	22	8.3	10	4.2
Putative	38	14.3	16	6.7
Probable	28	10.5	14	5.9
Generic[a]	13	4.9	20	8.4
ABC transporters[b]	35	13.1	19	8.0
Regulators[c]	5	1.9	13	5.5
Well-defined	58	21.8	83	35.0
Total	266		237	

[a]Annotated with only their generic functions (*e. g.*, as "oxidoreductase").
[b]Not including the "probable" ABC transporters.
[c]Only regulators with generic definition are included here.

Table S5. Proteins with well defined annotated functions from the differential pools, classified according to gene-onthology (GO) biologic processes. The rows in gray indicate proteins that, while being differentially expressed in one subcellular fraction, were not so between the Ara and Mtl cultures in the other.

Pool	Protein ID (Uniprot)	Gene name	Description	GO Biological process (Uniprot)
			L-arabinose oxidation pathways	
AraDP	Q89QH2	*frc*	Formyl-CoA:oxalate CoA-transferase.	oxalate catabolic process
	Q89QH1	*oxc*	Oxalyl-CoA decarboxylase.	oxalate catabolic process
	Q89QG2	*gcl*	Glyoxylate carboligase.	glyoxylate catabolic process
	Q89QG1	*hyi*	Hydroxypyruvate isomerase.	
	Q89QG0		Tartronate semialdehyde reductase.	glyoxylate metabolic process
	Q89QC2		Aldose 1-epimerase.	galactose catabolic process via UDP-galactose; glucose metabolic process
	Q89QC1		L-arabinonolactonase.	galactose catabolic process via UDP-galactose; glucose metabolic process
	Q89R43		Hydroxypyruvate isomerase.	
	Q89E00	*dgoA*	2-dehydro-3-deoxyphosphogalactonate aldolase.	metabolic process
			D-mannitol oxidation pathways	
MtlDP	Q8GKS1	*fbp*	Fructose-1,6-bisphosphatase class 1.	fructose 1,6-bisphosphate metabolic process; fructose 6-phosphate metabolic process; fructose metabolic process; gluconeogenesis; sucrose biosynthetic process
	H7C6I1	*cbbP*	Phosphoribulokinase.	carbohydrate metabolic process; pyrimidine nucleobase metabolic process; pyrimidine nucleoside salvage
	H7C6U6	*cbbT*	Transketolase.	metabolic process
	H7C7W4	*cbbA*	Fructose-bisphosphate aldolase.	glycolytic process
	Q9ZI34	*cbbL*	Ribulose bisphosphate carboxylase large chain.	carbon fixation; reductive pentose-phosphate cycle
	Q9ZI33	*cbbS*	Ribulose bisphosphate carboxylase small chain.	carbon fixation; reductive pentose-phosphate cycle
	Q89S24	*cbbE*	Ribulose-phosphate 3-epimerase.	cellular carbohydrate metabolic process; pentose catabolic process; pentose-phosphate shunt, non-oxidative branch
	Q89NT0	*gph*	Phosphoglycolate phosphatase.	carbohydrate metabolic process; dephosphorylation; DNA repair; glycolate biosynthetic process
	Q89QA4		Ribitol kinase.	carbohydrate metabolic process
	Q89NT9		Periplasmic mannitol-binding protein.	transport
			Metabolism of NAD and FAD	
AraDP	Q89S64	*nadB*	L-aspartate oxidase.	NAD biosynthetic process
	Q89LJ7	*nadE*	Glutamine-dependent NAD(+) synthetase.	NAD biosynthetic process
	Q89DI7	*ribH2*	6,7-dimethyl-8-ribityllumazine synthase 2.	riboflavin biosynthetic process

		Respiration		
AraDP	Q89WD0	*ubiE*	Ubiquinone/menaquinone biosynthesis C-methyltransferase.	menaquinone biosynthetic process; ubiquinone biosynthetic process
	Q89V68	*atpB*	ATP synthase subunit a.	ATP synthesis coupled proton transport
MtlDP	Q89XU2	*ubiG*	Ubiquinone biosynthesis O-methyltransferase.	ubiquinone biosynthetic process
	H7C6E5	*coxB*	Cytochrome c oxidase subunit 2.	ATP synthesis coupled electron transport
	Q89GX4		Rieske iron-sulfur protein; components of cytochrome bc1 complexes and cytochrome b6f complexes.	oxidation-reduction process
		Oxidative damage mitigation		
AdaDP	Q89UM2	*chaC*	Gamma-glutamylcyclotransferase.	glutathione catabolic process
	Q89U81	*ruvA*	Holliday junction ATP-dependent DNA helicase.	DNA recombination; DNA repair; SOS response
	Q89SJ6	*ggt*	Gamma-glutamyltranspeptidase.	glutathione catabolic process
	Q89RA0	*ggt*	Gamma-glutamyltranspeptidase.	glutathione catabolic process
	Q89F01	*ahpD*	Alkyl hydroperoxide reductase.	cellular oxidant detoxification
	Q89D86		Non-heme haloperoxidase.	cellular oxidant detoxification
MtlDP	Q89FD1		Malto-oligosyltrehalose trehalohydrolase.	trehalose biosynthetic process
	Q89V85	*attY*	Glutathione S-transferase.	
		Metabolism of nitrogen compounds		
AraDP	Q89UF7	*ureG*	Urease accessory protein.	nitrogen compound metabolic process
	Q89LP5	*nthA*	Nitrile hydratase subunit alpha.	nitrogen compound metabolic process
	Q89EJ6	*nirK*	Respiratory nitrite reductase.	nitrogen compound metabolic process
MtlDP	Q89UG3	*ureA*	Urease subunit gamma.	urea catabolic process
		Metabolism of aminoacids		
AraDP	Q89X27	*leuD*	3-isopropylmalate dehydratase small subunit.	leucine biosynthetic process
	Q89UM0	*tyrC*	Prephenate dehydrogenase.	oxidation-reduction process; tyrosine biosynthetic process
	Q89UL7	*metX*	Homoserine O-acetyltransferase.	homoserine metabolic process; methionine biosynthetic process
	P04772	*glnII*	Glutamine synthetase 2	glutamine biosynthetic process; nitrogen fixation
	Q89M51	*nifS*	Cysteine desulfurase.	cysteine metabolic process
	Q89M04	*aroG*	Phospho-2-dehydro-3-deoxyheptonate aldolase.	aromatic amino acid family biosynthetic process; chorismate biosynthetic process
	P59362	*glmS*	Glutamine-fructose-6-phosphate aminotransferase.	carbohydrate derivative biosynthetic process; glutamine metabolic process
	Q89IP8	*cysE*	Serine acetyltransferase.	cysteine biosynthetic process; cysteine biosynthetic process from serine

	Q89GL4	*bkdA1*	2-oxoisovalerate dehydrogenase alpha subunit.	branched-chain amino acid catabolic process
	Q89GL3	*bkdA2*	2-oxoisovalerate dehydrogenase beta subunit.	branched-chain amino acid catabolic process
	Q89DB4	*hisG*	ATP phosphoribosyltransferase.	histidine biosynthetic process
MtlDP	Q89XW8	*aroB*	3-dehydroquinate synthase.	aromatic amino acid family biosynthetic process; chorismate biosynthetic process
	Q89XH5		4-hydroxyphenylpyruvate dioxygenase.	tyrosine catabolic process
	Q89UJ7	*metF*	Methylenetetrahydrofolate reductase.	methionine biosynthetic process; one-carbon metabolic process; tetrahydrofolate interconversion
			Metabolism of polyhydroxybutyrate	
AraDP	Q89HC1	*phaC2*	Polyhydroxybutirate synthase PhaC2.	poly-hydroxybutyrate biosynthetic process
MtlDP	Q89XT0	*phaR*	Transcriptional regulator of polyhydroxybutirate synthesis.	regulation of transcription, DNA-templated
	Q89XT2	*phaB*	Acetoacetyl CoA reductase.	poly-hydroxybutyrate biosynthetic process
	Q89M33	*phaC1*	Poly-3-hydroxybutyrate synthase.	poly-hydroxybutyrate biosynthetic process
			Metabolism of biotin	
MtlDP	H7C6S8	*bioF*	8-amino-7-oxononanoate synthase.	biosynthetic process
	Q9AMS4	*bioD*	ATP-dependent dethiobiotin synthetase.	biotin biosynthetic process
	H7C6S2	*bioA*	Adenosylmethionine-8-amino-7-oxononanoate aminotransferase.	biotin biosynthetic process
			Metabolism of nucleotides	
AraDP	Q89KP5	*pyrH*	Uridylate kinase.	'de novo' CTP biosynthetic process
	Q89EH1	*purE*	N5-carboxyaminoimidazole ribonucleotide mutase.	'de novo' IMP biosynthetic process
	Q89E48	*upp*	Uracil phosphoribosyltransferase.	pyrimidine nucleobase metabolic process, pyrimidine nucleoside salvage; UMP salvage; uracil salvage
	Q89DJ1	*prs*	Ribose-phosphate pyrophosphokinase.	5-phosphoribose 1-diphosphate biosynthetic process; nucleoside metabolic process; nucleotide biosynthetic process; ribonucleoside monophosphate biosynthetic process
			Cobalamin synthesis	
AraDP	Q89Q72	*cobC*	Cobalamin biosynthetic protein.	cobalamin biosynthetic process
MtlDP	Q89Q71	*cobQ*	Cobyric acid synthase.	Cobalamin biosynthesis; glutamine metabolic process
			Cell motility	
AraDP	Q89WL4	*ctpF*	Pilus CtpF protein.	
	Q89UI1	*ctpG*	Pilus assembly protein.	transport

	Q89SQ7	*cheA*	Chemotaxis two-component sensor histidine kinase.	chemotaxis
	Q89SQ6	*cheWII*	CheWII protein.	chemotaxis
	Q89SQ5	*mcpK*	Methyl-accepting chemotaxis protein.	chemotaxis
	Q89SQ4	*cheW*	CheW protein.	chemotaxis
	Q89SQ3		Methyl-accepting chemotaxis protein.	chemotaxis
	Q89SQ1	*cheB*	Chemotaxis response regulator protein-glutamate methylesterase of group 3 operon.	chemotaxis
	Q89F43	*flgE*$_L$	Lateral flagellar hook protein	bacterial-type flagellum-dependent cell motility
	Q89F36	*lafA2*	Lateral flagellin 2	bacterial-type flagellum-dependent cell motility
	Q89F35	*lafA1*	Lateral flagellin 1	bacterial-type flagellum-dependent cell motility
MtlDP	Q89UH9	*ctpE*	Pilus assembly protein.	
	Q89T56	*cheY*	Chemotaxis two-component response regulator.	phosphorelay signal transduction system
			Protein synthesis and degradation	
AraDP	Q89UH1	*infA*	Translation initiation factor IF-1.	translation initiation factor activity
	Q89S35		Threonylcarbamoyl-AMP synthase.	regulation of translational fidelity; tRNA threonylcarbamoyladenosine modification
	Q89IN1	*gatA*	Glutamyl-tRNA amidotransferase subunit A.	
	Q89KT1	*ctpA*	Carboxy-terminal protease.	
MtlDP	Q89JW5	*clpS2*	ATP-dependent Clp protease adapter protein.	protein catabolic process
	Q89J84	*rplC*	50S ribosomal protein L3.	translation
			Protein folding	
AraDP	Q89VE7	*hslO*	60 kDa chaperonin.	protein folding
	P77829	*groL1*	60 kDa chaperonin 1.	'de novo' protein folding; chaperone-mediated protein folding; protein refolding
	Q89IK8	*groL6*	60 kDa chaperonin 6.	'de novo' protein folding; chaperone-mediated protein folding; protein refolding
MtlDP	Q89P00	*groL4*	60 kDa chaperonin 4.	'de novo' protein folding; chaperone-mediated protein folding; protein refolding
			Membrane and extracellular polysaccharide structure synthesis and degradation	
AraDP	Q89KQ5	*lpxA*	Acyl-[acyl-carrier-protein]-UDP-N-acetylglucosamine O-acyltransferase.	lipid A biosynthetic process
MtlDP	Q89VJ9		Long-chain-fatty-acid-CoA ligase.	long-chain fatty acid metabolic process
	Q89LD7	*glmU*	Bifunctional UDP-N-acetylglucosamine pyrophosphorylase and Glucosamine-1-phosphate N-acetyltransferase.	cell morphogenesis; cell wall organization; lipid A biosynthetic process; lipopolysaccharide biosynthetic process; peptidoglycan biosynthetic process; regulation of cell shape; UDP-N-acetylglucosamine biosynthetic process
	Q89CQ0		Capsule expression protein.	carbohydrate derivative metabolic process; carbohydrate metabolic process

		Transcriptional regulation		
AraDP	P30333	*rpoN2*	RNA polymerase sigma-54 factor 2.	DNA-templated transcription, initiation; nitrogen fixation
MtlDP	Q89VI3	*bjaR1*	Quorum sensing transcriptional activator protein.	quorum sensing; regulation of transcription, DNA-templated; transcription, DNA-templated
		Others		
AraDP	Q89WC6	*moeB*	Molybdopterin biosynthesis protein.	
	Q89WC2	*fabB*	3-oxoacyl-[acyl-carrier-protein] synthase I.	metabolic process
	Q89W56		Carboxymethylenebutenolidase.	
	Q89VV9	*ispG*	4-hydroxy-3-methylbut-2-en-1-yl diphosphate synthase (flavodoxin).	isopentenyl diphosphate biosynthetic process, methylerythritol 4-phosphate pathway; terpenoid biosynthetic process
	Q89UE1	*cysD*	Sulfate adenylate transferase subunit 2.	metabolic process
	Q89UD2	*cysA*	Sulfate/thiosulfate import ATP-binding protein.	ATPase-coupled sulfate transmembrane transport
	Q89SX8		Porin.	Carbohydrate transport; ion transport
	Q89RR4		Hippurate hydrolase. Metallodipeptidase activity	metabolic process
	Q89RE6	*ilvD*	Dihydroxy-acid dehydratase.	metabolic process
	Q89QR6		7,8-dihydroneopterin aldolase.	folic acid biosynthetic process; tetrahydrofolate biosynthetic process
	Q89PU2		3-oxoacyl-(Acyl carrier protein) reductase.	metabolic process
	Q89NC9	*fabG*	3-oxoacyl-(Acyl-carrier protein) reductase.	
	Q89NC7		Dihydroxy-acid dehydratase.	metabolic process
	Q89NC0	*kdgK*	2-dehydro-3-deoxygluconokinase.	
	Q89L91		Beta-glucosidase.	cellulose catabolic process; glycosyl compound metabolic process; single-organism carbohydrate catabolic process
	Q89GL2	*bkdB*	Lipoamide acyltransferase component of branched-chain alpha-keto acid dehydrogenase complex E2.	metabolic process
	Q89FP4	*thiO*	Thiamine biosynthesis oxidoreductase.	
	Q89FP0	*thiC*	Phosphomethylpyrimidine synthase. Thiamine biosynthesis protein.	thiamine biosynthetic process; thiamine diphosphate biosynthetic process
	Q89F88	*iivG*	Acetolactate synthase large subunit.	
	Q89W59	*msrB*	Peptide methionine sulfoxide reductase.	cellular protein modification process
	Q89DJ5	*lgt*	Prolipoprotein diacylglyceryl transferase.	lipoprotein biosynthetic process; protein lipoylation
	Q89DH3		Ribonuclease.	RNA phosphodiester bond hydrolysis, endonucleolytic
	Q89D67	*metA*	O-methyl transferase.	
MtlDP	Q89WG1	*hspH*	Small heat shock protein.	
	Q89WA0		Magnesium and cobalt efflux protein.	oxidation-reduction process
	Q89VW7	*pcaG*	Protocatechuate 3,4-dioxygenase alpha chain.	aromatic compound catabolic process
	Q89LX3	*mccB*	3-methylcrotonoyl-CoA carboxylase beta subunit.	
	Q89VF2	*pstB*	Phosphate import ATP-binding protein.	phosphate ion transmembrane transport; ATP hydrolysis coupled anion transmembrane transport

Q89UV0	*acs*	Acetyl-coenzyme A synthetase.	metabolic process
Q89UA2	*acrA*	RND multidrug efflux membrane permease.	drug transmembrane transport; response to antibiotic
Q89T39	*copB*	Copper tolerance protein.	transport
Q89T29	*bioA*	Adenosylmethionine-8-amino-7-oxononanoate aminotransferase.	
Q89RY8	*pdxH*	Pyridoxine/pyridoxamine 5'-phosphate oxidase.	pyridoxal phosphate biosynthetic process; pyridoxine biosynthetic process
Q89R91	*ephA*	Epoxide hydrolase.	
Q89R75	*paaK*	Phenylacetate-coenzyme A ligase.	phenylacetate catabolic process
Q89NP2	*psd*	Phosphatidylserine decarboxylase proenzyme.	phosphatidylethanolamine biosynthetic process
Q89NF3	*pdxA1*	4-hydroxythreonine-4-phosphate dehydrogenase 1. Vitamin B6 metabolism.	carbohydrate metabolic process; pyridoxine biosynthetic process
Q89MR5		Cold shock protein.	regulation of transcription, DNA-templated
Q89LX4	*acd*	Acyl-CoA dehydrogenase.	oxidation-reduction process
Q89LX2	*mccA*	3-methylcrotonyl-CoA carboxylase alpha subunit.	
Q89K83	*hppA*	K(+)-insensitive pyrophosphate-energized proton pump.	proton transport
Q89F71	*uxuA*	Mannonate dehydratase.	D-glucuronate catabolic process
Q89DH1	*cspA*	Cold shock protein.	regulation of transcription, DNA-templated
Q89C99		Alcohol dehydrogenase.	oxidation-reduction process
Q89BN2		DNA polymerase III tau subunit.	DNA-dependent DNA replication

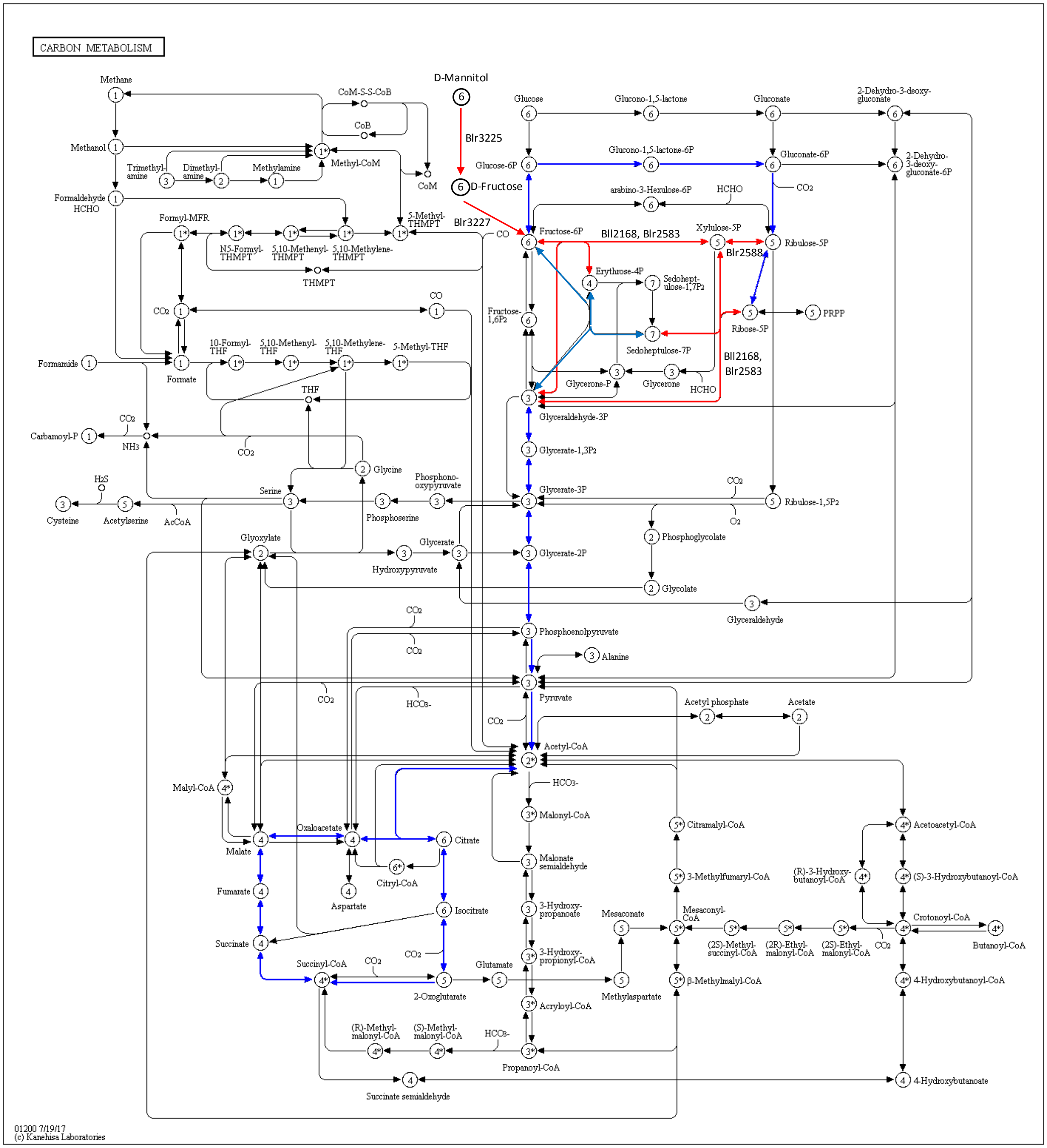

Fig. S2 Mtl catabolism through the PP pathway. The reactions (indicated by the corresponding Rhizobase code, as in text Fig. 1) in red are catalyzed by the enzymes detected in the MtlDP and in blue by the enzymes detected in the general pool but not differentially expressed between Ara- and Mtl-grown cultures. The number of C atoms of each metabolite is indicated inside circles.

The map was constructed with KEGG Mapper (http://www.genome.jp/kegg/tool/map_pathway2.html) from the list of proteins from Table S6 and the carbon metabolism (bja01200) map as a template. D-mannitol and D-fructose were added to the map by hand. The reaction catalyzed by Blr6758 (blue) was colored by hand because, although that step was annotated in the pentose-phosphate–pathway KEGG map (bja00030), the reaction was not annotated in the carbon-metabolism (bja01200) map.

Table S6. Stoichiometric balance of the predicted reactions for the catabolism of D-mannitol in the pentose-phosphate (PP) pathway. The enzymes in red belong to the MtlDP; the underlined enzymes were not observed in the Ara cultures. All the other enzymes were present in our samples, as detailed in Table S1.

Substrates	Products	Enzymes detected (Rhizobase code)
1 D-mannitol + 1 NAD^+	1 D-fructose + 1 NADH + 1 H^+	Blr3225
1 D-fructose + 1 ATP	1 D-fructose-6P + 1 ADP	Blr3227
3 D-fructose-6P	3 D-glucose-6P	Blr6758
3 D-glucose-6P + 3 NAD^+	3 D-glucono-1,5-lactone-6P + 3 NADH+ 3 H^+	Blr6760
3 D-glucono-1,5-lactone-6P + 3H_2O	3 D-gluconate-6P	Blr0368
3 D-gluconate-6P + 3 NAD^+	3 D-ribulose-5P + 3 CO_2 + 3 NADH+ 3 H^+	Blr6759
1 D-ribulose-5P	1 D-ribose-5P	Blr3755
2 D-ribulose-5P	2 D-xylulose-5P	Blr2588
1 ribose-5P + 1 D-xylulose-5P	1 D-sedoheptulose-7P + 1 D-glyceraldehyde-3P	Bll2168, Blr2583
1 D-sedoheptulose-7P + 1 D-glyceraldehyde-3P	1 D-fructose-6P + 1 D-erythrose-4P	Blr6758
1 D-erythrose-4P + 1 D-xylulose-5P	1 D-fructose-6P + 1 glyceraldehyde-3P	Bll2168, Blr2583
1 glyceraldehyde-3P + 1 Pi +1 NAD^+	1 glycerate-1,3P_2 + 1 NADH + 1 H^+	Bll1523
1 glycerate-1,3P_2 + 1 ADP	1 glycerate-3P + 1 ATP	Bll1522
1 glycerate-3P	1 glycerate-2P	Blr2630
1 glycerate-2P	1 phosphoenolpyruvate + 1 H_2O	Bll4794
1 phosphoenolpyruvate + 1 ADP	1 pyruvate + 1 ATP	Blr7138
1 pyruvate + 1 CoA + 1 NAD^+	1 acetyl-CoA + 1 CO_2 + 1 NADH + 1 H^+	Bll0449, Bll4778, Bll4779, Bll4782, Bll4783, Blr2815, Blr4839
1 acetyl-CoA + 1 oxaloacetate + 1 H_2O	1 citrate + 1 CoA	Bll0182, Blr4839
1 citrate	1 isocitrate	Bll0466
1 isocitrate + 1 NAD^+	1 α-ketoglutarate + 1 CO_2 + 1 NADH + 1 H^+	Blr5747
1 α-ketoglutarate + 1 CoA + 1 NAD^+	1 succinyl-CoA + 1 CO_2 + 1 NADH + 1 H^+	Bll0449, Bll0451, Bll0452, Bll4778, Blr6334
1 succinyl-CoA + 1 GDP + 1 Pi	1 succinate + 1 CoA + 1 GTP	Bll0455
1 succinate + 1 FAD	1 fumarate + 1 $FADH_2$	Blr0514, Blr0515
1 fumarate + 1 H_2O	1 malate	Bll0286, Bll5796, Blr6519
1 malate + 1 NAD^+	1 oxaloacetate + 1 NADH + 1 H^+	Bll0456

Balance:

1 $C_6H_{14}O_6$ (Mtl) + 12 NAD^+ + 1 FAD + 1 ADP + 1 GDP + 2 Pi + 4 H_2O + $\rightarrow$
6 CO_2 + 12 NADH + 12 H^+ + 1 $FADH_2$ + 1 ATP + 1 GTP

ATP yield:

[ATP + GTP]:	1 + 1	= 2
NADH:	12×3	= 36
$FADH_2$:	1×2	= 2
Total:		40
ATP/Mtl:		40.0
ATP/C-mole:		6.7

O_2 consumed:

From pathway		0
Rx 1: [$2\ NADH + 2\ H^+ + O_2 \rightarrow 2\ NAD^+ + 2\ H_2O$]:	12/2	= 6.0
Rx 2: [$2\ FADH_2 + O_2 \rightarrow 2\ FAD + 2\ H_2O$]:	1/2	= 0.5
Total:		6.5
O_2/Mtl:		6.5
O_2/C-mole:		1.1

Pyruvate produced (before TCA):

Total:	1
Pyruvate/Mtl:	1.0
Pyruvate/C-mole:	0.2

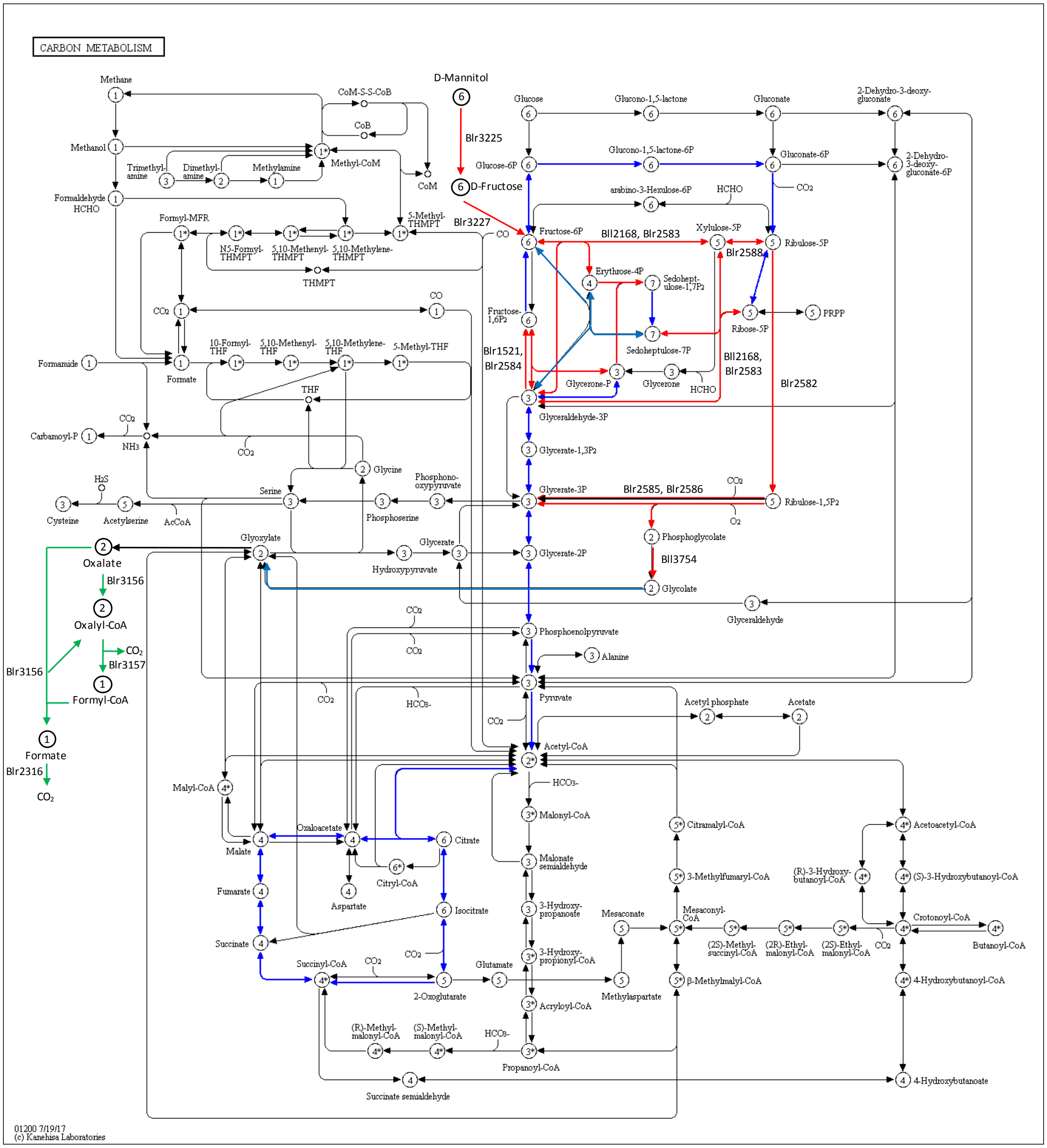

Fig. S3 Mtl catabolism through the CBB pathway with RuBisCO catalysis involving 100% oxygenase activity along with glyoxylate oxidation occurring through the oxalate pathway. The reactions (indicated by their Rhizobase code) in red are catalyzed by the enzymes detected in the MtlDP; in green, by the enzymes detected in the AraDP; and in blue, by the enzymes detected in the general pool, but not differentially expressed between Ara- and Mtl-grown cultures. The number of C atoms of each metabolite is indicated inside circles.

The map was constructed with KEGG Mapper (http://www.genome.jp/kegg/tool/map_pathway2.html) from the list of proteins from Table S7 and the carbon-metabolism (bja01200) map as a template. D-mannitol, D-fructose, and the oxalate-oxidation pathway were added to the map by hand. The reactions catalyzed by Blr6758, Bll7540, Bll7541, Bll7543 (all in blue), and Bll3754 (in red) were colored by hand because, although those steps were annotated in the pentose-phosphate–pathway (bja00030) and the glyoxylate- and carboxylate-metabolism (bja00630) KEGG maps, the reactions were not annotated in the carbon-metabolism (bja01200) map. The RuBisCO carboxylase activity has been colored in black to indicate the absence of that catalysis in this model.

Table S7. Stoichiometric balance of the predicted reactions for the catabolism of D-mannitol under the condition of oxygenase as being the sole activity in the RuBisCO catalysis and with glyoxylate oxidation in the oxalate pathway. The enzymes in red belong to the MtlDP and those in green to the AraDP. The red underlined enzymes belonging to the MtlDP were not observed in the Ara cultures, while the green underlined enzymes belonging to AraDP were not observed in the Mtl cultures. All the other enzymes were present in our samples, as detailed in Table S1.

**Substrates**	**Products**	**Enzymes detected (Rhizobase code)**
3 D-mannitol + 3 NAD^+	3 D-fructose + 3 NADH + 3 H^+	Blr3225
3 D-fructose + 3 ATP	3 D-fructose-6P + 3 ADP	Blr3227
6 D-fructose-6P	6 D-glucose-6P	Blr6758
6 D-glucose-6P + 6 NAD^+	6 D-glucono-1,5-lactone-6P + 6 NADH+ 6 H^+	Blr6760
6 D-glucono-1,5-lactone-6P + 6 H_2O	6 D-gluconate-6P	Blr0368
6 D-gluconate-6P + 6 NAD^+	6 D-ribulose-5P + 6 CO_2 + 6 NADH+ 6 H^+	Blr6759
1 D-ribulose-5P	1 D-ribose-5P	Blr3755
2 D-ribulose-5P	2 D-xylulose-5P	Blr2588
1 D-ribose-5P + 1 D-xylulose-5P	1 D-sedoheptulose-7P + 1 D-glyceraldehyde-3P	Bll2168, Blr2583
2 D-sedoheptulose-7P + 2 glyceraldehyde-3P	2 D-fructose-6P + 2 D-erythrose-4P	Blr6758
1 D-erythrose-4P + 1 D-xylulose-5P	1 D-fructose-6P + 1 D-glyceraldehyde-3P	Bll2168, Blr2583
1 D-glyceraldehyde-3P	1 glycerone-P	Bll4807
1 D-erythrose-4P + 1 glycerone-P	1 D-sedoheptulose-1,7P_2	Blr1521, Blr2584
1 D-sedoheptulose-1,7P_2 + 1 H_2O	1 D-sedoheptulose-7P + 1 Pi	Blr4363
3 D-ribulose-5P + 3 ATP	3 D-ribulose-1,5P_2 + 3 ADP	Blr2582
3 D-ribulose-1,5P_2 + 3 O_2	3 phosphoglycolate + 3 glycerate-3P	Blr2585, Blr2586
3 phosphoglycolate + 3 H_2O	3 glycolate + 3 Pi	Bll3754
3 glycolate + 3 O_2	3 glyoxylate + 3 H_2O_2	Bll7540, Bll7541, Bll7543
3 glyoxylate + 3 O_2 + 3 H_2O	3 oxalate + 3 H_2O_2	Non-Annotated
3 oxalate + 3 formyl-CoA	3 oxalyl-CoA + 3 formate	Bll3156
3 oxalyl-CoA	3 formyl-CoA + 6 CO_2	Bll3157
3 formate + 3 NAD^+	3 CO_2 + 3 NADH + 3 H^+	Blr2316, Blr2317
1 glycerate-3P + 1 ATP	1 glycerate-1,3P_2 + 1 ADP	Bll1522
1 glycerate-1,3P_2 + 1 NADH + 1 H^+	1 D-glyceraldehyde-3P + 1 Pi + 1 NAD^+	Bll1523
2 glycerate-3P	2 glycerate-2P	Blr2630
2 glycerate-2P	2 phosphoenolpyruvate + 2 H_2O	Bll4794
2 phosphoenolpyruvate + 2 ADP	2 pyruvate + 2 ATP	Blr7138
2 pyruvate + 2 CoA + 2 NAD^+	2 acetyl-CoA + 2 CO_2 + 2 NADH + 2 H^+	Bll0449, Bll4778, Bll4779, Bll4782, Bll4783, Blr2815, Blr4839
2 acetyl-CoA + 2 oxalacetate + 2 H_2O	2 citrate + 2 CoA	Bll0182, Blr4839
2 citrate	2 isocitrate	Bll0466
2 isocitrate + 2 NAD^+	2 α-ketoglutarate + 2 CO_2 + 2 NADH + 2 H^+	Blr5747
2 α-ketoglutarate + 2 CoA + 2 NAD^+	2 succinyl-CoA + 2 CO_2 + 2 NADH + 2 H^+	Bll0449, Bll0451, Bll0452, Bll4778, Blr6334
2 succinyl-CoA + 2 GDP + 2 Pi	2 succinate + 2 CoA + 2 GTP	Bll0455

2 succinate + 2 FAD	2 fumarate + 2 $FADH_2$	Blr0514, Blr0515
2 fumarate + 2 H_2O	2 malate	Bll0286, Bll5796, Blr6519
2 malate + 2 NAD^+	2 oxalacetate + 2 NADH + 2 H^+	Bll0456

Balance:

3 $C_6H_{14}O_6$ (Mtl) + 25 NAD^+ + 2 FAD + 5 ATP + 2 GDP + 15 H_2O + 9 O_2 $\rightarrow$
18 CO_2 + 25 NADH + 25 H^+ + 2 $FADH_2$ + 5 ADP + 2 GTP + 3 Pi + 6 H_2O_2

ATP yield:

[ATP + GTP]:	$-5 + 2$	=	-3
NADH:	25×3	=	75
$FADH_2$:	2×2	=	4
Total:			76
ATP/Mtl:			25.3
ATP/C-mole:			4.2

O_2 consumed:

From pathway			9
Rx 1: [2 NADH + 2 H^+ + O_2 $\rightarrow$ 2 NAD^+ + 2 H_2O]:	25/2	=	12.5
Rx 2: [2 $FADH_2$ + O_2 $\rightarrow$ 2 FAD + 2 H_2O]:	2/2	=	1.0
Total:			13.5
O_2/Mtl:			7.5
O_2/C-mole:			1.2

Pyruvate produced (before TCA):

Total:	2
Pyruvate/Mtl:	0.7
Pyruvate/C-mole:	0.1

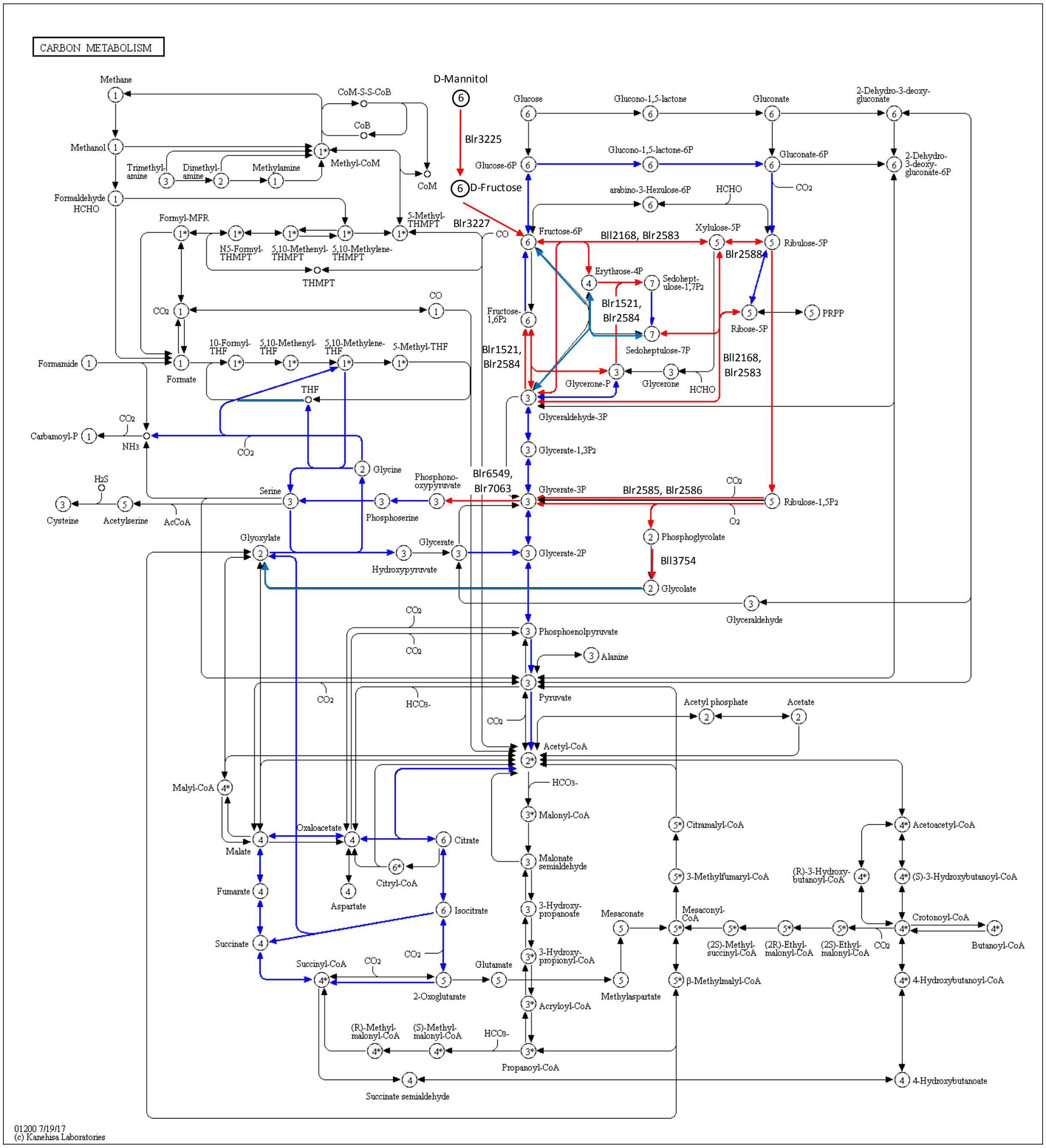

Fig. S4 Mtl catabolism through the CBB pathway with RuBisCO catalysis involving 100% oxygenase activity along with C-recycling though the THF cycle. The reactions (indicated by their Rhizobase code) in red are catalyzed by the enzymes detected in the MtlDP; in blue, by the enzymes detected in the general pool, but not differentially expressed between Ara- and Mtl-grown cultures. The number of C atoms of each metabolite is indicated inside circles.
The map was constructed with KEGG Mapper (http://www.genome.jp/kegg/tool/map_pathway2.html) from the list of proteins from Table S8 and the carbon-metabolism (bja01200) map as a template. D-mannitol and D-fructose were added to the map by hand. The reactions catalyzed by Blr6758, Bll7540, Bll7541, Bll7543 (all in blue), and Bll3754 (in red) were colored by hand because, although those steps were annotated in the pentose-phosphate–pathway (bja00030) and the glyoxylate- and carboxylate-metabolism (bja00630) KEGG maps, the reactions were not annotated in the carbon-metabolism (bja01200) map. The RuBisCO carboxylase activity has been colored in black to indicate its absence in this model

Table S8. Stoichiometric balance of the predicted reactions for the catabolism of D-mannitol under the condition of oxygenase as being the sole activity in the RuBisCO catalysis and with carbon recycling through the tetrahydrofolate (THF) and glyoxylate cycles. The enzymes in red belong to the MtlDP; the underlined enzymes were not observed in the Ara cultures. All the other enzymes were present in our samples, as detailed in Table S1.

Substrates	Products	Enzymes detected (Rhizobase code)
3 D-mannitol + 3 NAD^+	3 D-fructose + 3 NADH + 3 H^+	Blr3225
3 D-fructose + 3 ATP	3 D-fructose-6P + 3 ADP	Blr3227
6 D-fructose-6P	6 D-glucose-6P	Blr6758
6 D-glucose-6P + 6 NAD^+	6 D-glucono-1,5-lactone-6P + 6 NADH+ 6 H^+	Blr6760
6 D-glucono-1,5-lactone-6P + 6 H_2O	6 D-gluconate-6P	Blr0368
6 D-gluconate-6P + 6 NAD^+	6 D-ribulose-5P + 6 CO_2 + 6 NADH+ 6 H^+	Blr6759
1 D-ribulose-5P	1 D-ribose-5P	Blr3755
2 D-ribulose-5P	2 D-xylulose-5P	Blr2588
1 D-ribose-5P + 1 D-xylulose-5P	1 D-sedoheptulose-7P + 1 D-glyceraldehyde-3P	Bll2168, Blr2583
2 D-sedoheptulose-7P + 2 glyceraldehyde-3P	2 D-fructose-6P + 2 D-erythrose-4P	Blr6758
1 D-erythrose-4P + 1 D-xylulose-5P	1 D-fructose-6P + 1 D-glyceraldehyde-3P	Bll2168, Blr2583
1 D-glyceraldehyde-3P	1 glycerone-P	Bll4807
1 D-erythrose-4P + 1 glycerone-P	1 D-sedoheptulose-1,7P_2	Blr1521, Blr2584
1 D-sedoheptulose-1,7P_2 + 1 H_2O	1 D-sedoheptulose-7P + 1 Pi	Blr4363
3 D-ribulose-5P + 3 ATP	3 D-ribulose-1,5P_2 + 3 ADP	Blr2582
3 D-ribulose-1,5P_2 + 3 O_2	3 phosphoglycolate + 3 glycerate-3P	Blr2585, Blr2586
3 phosphoglycolate + 3 H_2O	3 glycolate + 3 Pi	Bll3754
3 glycolate + 3 O_2	3 glyoxylate + 3 H_2O_2	Bll7540, Bll7541, Bll7543
3 glycerate-3P + 3 NAD^+	3 3-phosphonooxypyruvate + 3 NADH + 3 H^+	Bll2918, Bll6549, Bll6814, Bll7401, Bll7965, Blr3173, Blr4558, Blr7063
3 3-phosphonooxypyruvate + 3 L-glutamate	3 phospho-L-serine + 3 α-ketoglutarate	Bll7402
3 phospho-L-serine + 3 H_2O	3 L-serine + 3 Pi	Blr6505
3 L-glycine + 3 THF + 3 NAD^+	3 5,10-methenyl-THF + 3 NH_4^+ + 3 CO_2 + 3 NADH + 3 H^+	Bll0449, Bll4778, Blr5751, Blr5753, Blr6334
3 L-glycine + 3 5,10-methenyl-THF + 3 H_2O	3 L-serine + 3 THF	Bll5033, Bll5912
3 α-ketoglutarate + 3 NH_4^+ + 3 NADH + 3 H^+	3 L-glutamate + 3 NAD^+ + 3 H_2O	Blr7995
1 glycerate-2P	1 glycerate-3P	Blr2630
1 glycerate-3P + 1 ATP	1 glycerate-1,3P_2 + 1 ADP	Bll1522
1 glycerate-1,3P_2 + 1 NADH + 1 H^+	1 D-glyceraldehyde-3P + 1 Pi + 1 NAD^+	Bll1523
5 glycerate-2P	5 phosphoenolpyruvate + 5 H_2O	Bll4794
5 phosphoenolpyruvate + 5 ADP	5 pyruvate + 5 ATP	Blr7138
5 pyruvate + 5 CoA + 5 NAD^+	5 acetyl-CoA + 5 CO_2 + 5 NADH + 5 H^+	Bll0449, Bll4778, Bll4779, Bll4782, Bll4783, Blr2815, Blr4839
5 acetyl-CoA + 5 oxalacetate + 5 H_2O	5 citrate + 5 CoA	Bll0182, Blr4839
5 citrate	5 isocitrate	Bll0466
3 isocitrate	3 glyoxylate + 3 succinate	Blr2455

6 glyoxylate + 6 L-serine	6 hydroxypyruvate + 6 L-glycine	Blr0373, Bll6037
6 hydroxypyruvate + 6 NADH + 6 H^+	6 glycerate + 6 NAD^+	Non-annotated
6 glycerate + 6 ATP	6 glycerate-2P + 6 ADP	Blr7830
2 isocitrate + 2 NAD^+	2 α-ketoglutarate + 2 CO_2 + 2 NADH + 2 H^+	Blr5747
2 α-ketoglutarate + 2 CoA + 2 NAD^+	2 succinyl-CoA + 2 CO_2 + 2 NADH + 2 H^+	Bll0449, Bll0451, Bll0452, Bll4778, Blr6334
2 succinyl-CoA + 2 GDP + 2 Pi	2 succinate + 2 CoA + 2 GTP	Bll0455
5 succinate + 5 FAD	5 fumarate + 5 $FADH_2$	Blr0514, Blr0515
5 fumarate + 5 H_2O	5 malate	Bll0286, Bll5796, Blr6519
5 malate + 5 NAD^+	5 oxalacetate + 5 NADH + 5 H^+	Bll0456

Balance:

$$3\ C_6H_{14}O_6\ (\text{Mtl}) + 25\ NAD^+ + 5\ FAD + 8\ ATP + 2\ GDP + 18\ H_2O + 6\ O_2 \rightarrow$$
$$18\ CO_2 + 25\ NADH + 25\ H^+ + 5\ FADH_2 + 8\ ADP + 2\ GTP + 6\ Pi + 3\ H_2O_2$$

ATP yield:

[ATP + GTP]:	$-8 + 2$	=	-6
NADH:	25×3	=	75
$FADH_2$:	5×2	=	10
Total:			79
ATP/Mtl:			26.3
ATP/C-mole:			4.4

O_2 consumed:

From pathway			6
Rx 1: [$2\ NADH + 2\ H^+ + O_2 \rightarrow 2\ NAD^+ + 2\ H_2O$]:	25/2	=	12.5
Rx 2: [$2\ FADH_2 + O_2 \rightarrow 2\ FAD + 2\ H_2O$]:	5/2	=	2.5
Total:			21.0
O_2/Mtl:			7.0
O_2/C-mole:			1.2

Pyruvate produced (before TCA):

Total:	5
Pyruvate/Mtl:	1.7
Pyruvate/C-mole:	0.3

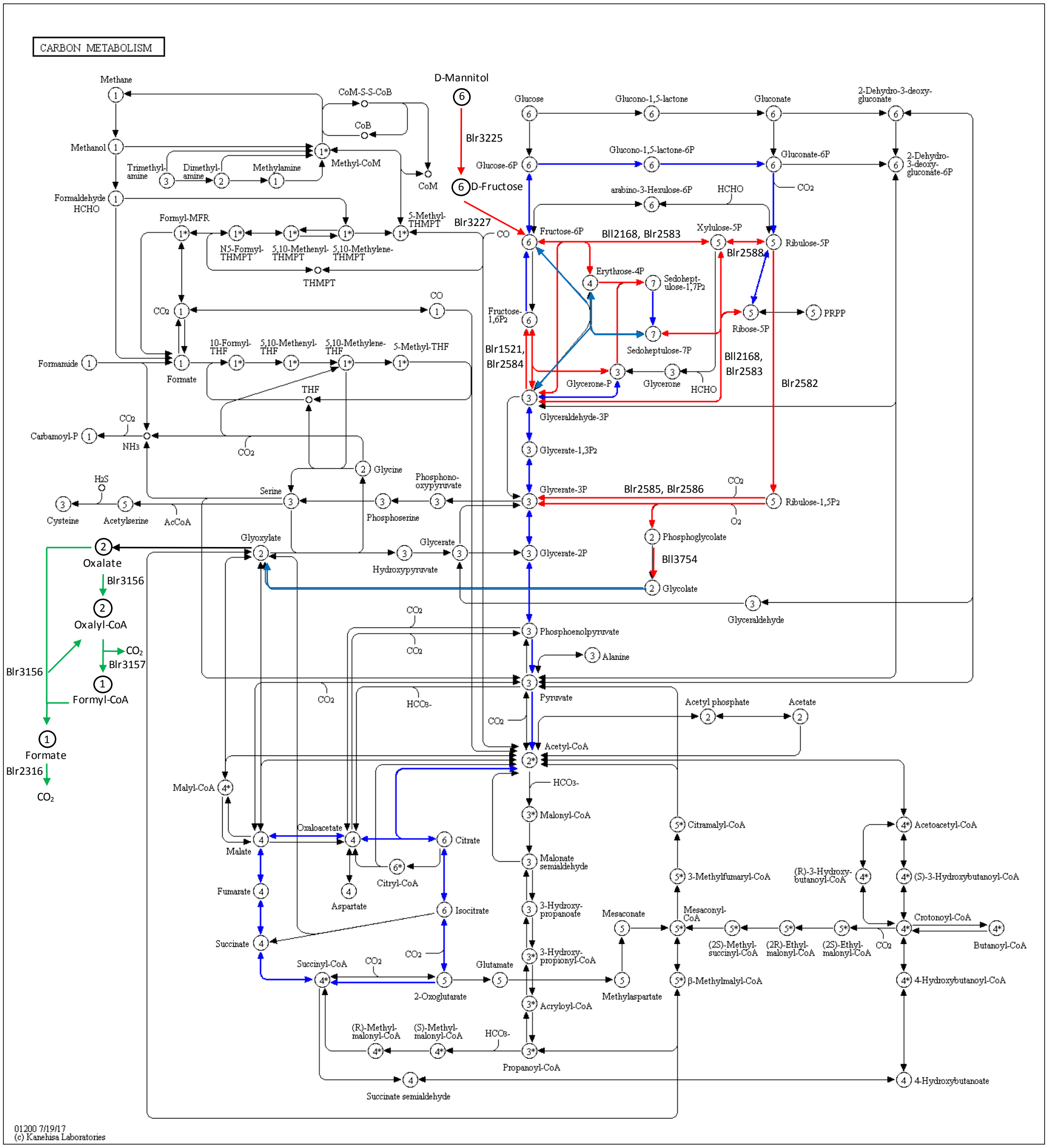

Fig. S5 Mtl catabolism through the CBB pathway with 50% oxygenase and 50% carboxylase activities in the RuBisCO catalysis and glyoxylate oxidation through the oxalate pathway. The reactions (indicated by their Rhizobase code) in red are catalyzed by the enzymes detected in the MtlDP; in green, by the enzymes detected in the AraDP; in blue, by the enzymes detected in the general pool, but not differentially expressed between Ara- and Mtl-grown cultures. The number of C atoms of each metabolite is indicated inside circles.

The map was constructed with KEGG Mapper (http://www.genome.jp/kegg/tool/map_pathway2.html) from the list of proteins from Table S9 and the carbon-metabolism (bja01200) map as a template. D-mannitol, D-fructose, and the oxalate oxidation pathway were added to the map by hand. The reactions catalyzed by Blr6758, Bll7540, Bll7541, Bll7543 (all in blue), and Bll3754 (in red) were colored by hand because, although those steps were annotated in the pentose-phosphate–pathway (bja00030) and the glyoxylate- and carboxylate-metabolism (bja00630) KEGG maps, the reactions were not annotated in the carbon-metabolism (bja01200) map.

Table S9. Stoichiometric balance of the predicted reactions for the catabolism of D-mannitol under the condition of 50% oxygenase and 50% carboxylase activities in the RuBisCO catalysis and with glyoxylate oxidation in the oxalate pathway. The enzymes in red belong to the MtlDP and those in green to the AraDP. The red underlined enzymes belonging to the MtlDP were not observed in the Ara cultures, while green underlined enzymes belonging to AraDP were not observed in the Mtl cultures. All the other enzymes were present in our samples, as detailed in Table S1.

**Substrates**	**Products**	**Enzymes detected (Rhizobase code)**
6 D-mannitol + 6 NAD^+	6 D-fructose + 6 NADH + 6 H^+	Blr3225
6 D-fructose + 6 ATP	6 D-fructose-6P + 6 ADP	Blr3227
12 D-fructose-6P	12 D-glucose-6P	Blr6758
12 D-glucose-6P + 12 NAD^+	12 D-glucono-1,5-lactone-6P + 12 NADH+ 12 H^+	Blr6760
12 D-glucono-1,5-lactone-6P + 12 H_2O	12 D-gluconate-6P	Blr0368
12 D-gluconate-6P + 12 NAD^+	12 D-ribulose-5P + 12 CO_2 + 12 NADH+ 12 H^+	Blr6759
2 D-ribulose-5P	2 D-ribose-5P	Blr3755
4 D-ribulose-5P	4 D-xylulose-5P	Blr2588
2 D-ribose-5P + 2 D-xylulose-5P	2 D-sedoheptulose-7P + 2 D-glyceraldehyde-3P	Bll2168, Blr2583
4 D-sedoheptulose-7P + 4 glyceraldehyde-3P	4 D-fructose-6P + 4 D-erythrose-4P	Blr6758
2 D-erythrose-4P + 2 D-xylulose-5P	2 D-fructose-6P + 2 D-glyceraldehyde-3P	Bll2168, Blr2583
2 D-glyceraldehyde-3P	2 glycerone-P	Bll4807
2 D-erythrose-4P + 2 glycerone-P	2 D-sedoheptulose-1,7P_2	Blr1521, Blr2584
2 D-sedoheptulose-1,7P_2 + 2 H_2O	2 D-sedoheptulose-7P + 2 Pi	Blr4363
6 D-ribulose-5P + 6 ATP	6 D-ribulose-1,5P_2 + 6 ADP	Blr2582
3 D-ribulose-1,5P_2 + 3 O_2	3 phosphoglycolate + 3 glycerate-3P	Blr2585, Blr2586
3 D-ribulose-1,5P_2+ 3 CO_2 + 3 H_2O	6 glycerate-3P	Blr2585, Blr2586
3 phosphoglycolate + 3 H_2O	3 glycolate + 3 Pi	Bll3754
3 glycolate + 3 O_2	3 glyoxylate + 3 H_2O_2	Bll7540, Bll7541, Bll7543
3 glyoxylate + 3 O_2 + 3 H_2O	3 oxalate + 3 H_2O_2	Non-annotated
3 oxalate + 3 formyl-CoA	3 oxalyl-CoA + 3 formate	Bll3156
3 oxalyl-CoA	3 formyl-CoA + 3 CO_2	Bll3157
3 formate + 3 NAD^+	3 CO_2 + 3 NADH + 3 H^+	Blr2316, Blr2317
2 glycerate-3P + 2 ATP	2 glycerate-1,3P_2 + 2 ADP	Bll1522
2 glycerate-1,3P_2 + 2 NADH + 2 H^+	2 D-glyceraldehyde-3P + 2 Pi + 2 NAD^+	Bll1523
7 glycerate-3P	7 glycerate-2P	Blr2360
7 glycerate-2P	7 phosphoenolpyruvate + 7 H_2O	Bll4794
7 phosphoenolpyruvate + 7 ADP	7 pyruvate + 7 ATP	Blr7138
7 pyruvate + 7 CoA + 7 NAD^+	7 acetyl-CoA + 7 CO_2 + 7 NADH + 7 H^+	Bll0449, Bll4778, Bll4779, Bll4782, Bll4783, Blr2815, Blr4839
7 acetyl-CoA + 7 oxalacetate + 7 H_2O	7 citrate + 7 CoA	Bll0182, Blr4839
7 citrate	7 isocitrate	Bll0466
7 isocitrate + 7 NAD^+	7 α-ketoglutarate + 7 CO_2 + 7 NADH + 7 H^+	Blr5747
7 α-ketoglutarate + 7 CoA + 7 NAD^+	7 succinyl-CoA + 7 CO_2 + 7 NADH + 7 H^+	Bll0449, Bll0451, Bll0452, Bll4778, Blr6334

7 succinyl-CoA + 7 GDP + 7 Pi	7 succinate + 7 CoA + 7 GTP	Bll0455
7 succinate + 7 FAD	7 fumarate + 7 $FADH_2$	Blr0514, Blr0515
7 fumarate + 7 H_2O	7 malate	Bll0286, Bll5796, Blr6519
7 malate + 7 NAD^+	7 oxalacetate + 7 NADH + 7 H^+	Bll0456

Balance:

$6\ C_6H_{14}O_6$ (Mtl) + 59 NAD^+ + 7 FAD + 7 ATP + 7 GDP + 30 H_2O + 9 $O_2 \rightarrow$
36 CO_2 + 59 NADH + 59 H^+ + 7 $FADH_2$ + 7 ADP + 7 GTP + + 6 H_2O_2

ATP yield:

[ATP + GTP]:	–7 + 7	=	0
NADH:	59×3	=	177
$FADH_2$:	7×2	=	14
Total:			191
ATP/Mtl:			31.8
ATP/C-mole:			5.3

O_2 consumed:

From pathway			9
Rx 1: [2 NADH + 2 H^+ + $O_2 \rightarrow$ 2 NAD^+ + 2 H_2O]:	59/2	=	29.5
Rx 2: [2 $FADH_2$ + $O_2 \rightarrow$ 2 FAD + 2 H_2O]:	7/2	=	3.5
Total:			42.0
O_2/Mtl:			7.0
O_2/C-mole:			1.2

Pyruvate produced (before TCA):

Total:	7
Pyruvate/Mtl:	1.2
Pyruvate/C-mole:	0.2

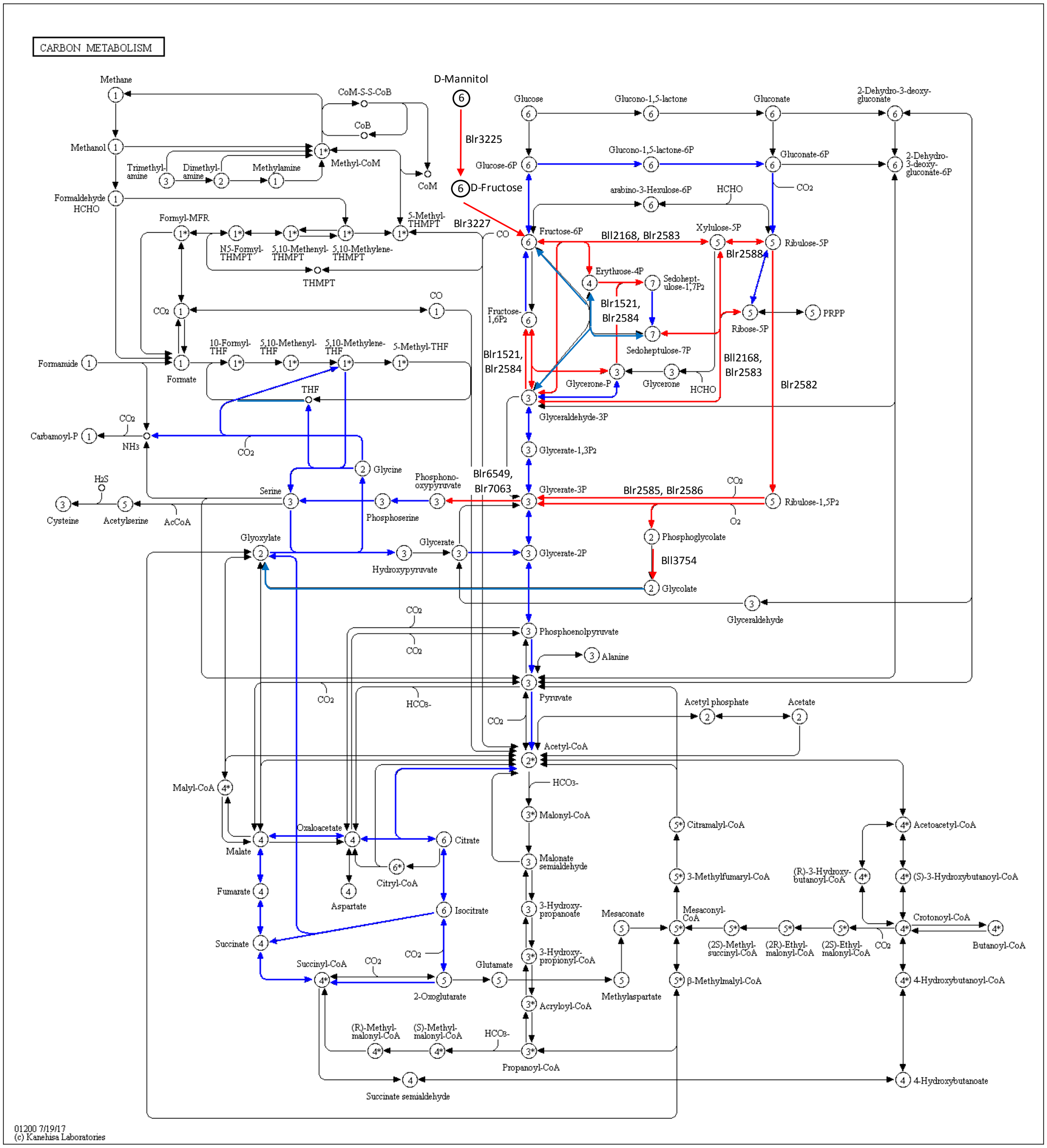

Fig. S6 Mtl catabolism through the CBB pathway with 50% oxygenase and 50% carboxylase activities in the RuBisCO catalysis and C-recycling though the THF cycle. The reactions (indicated by their Rhizobase code) in red are catalyzed by the enzymes detected in the MtlDP; in blue, by the enzymes detected in the general pool, but not differentially expressed between Ara- and Mtl-grown cultures. The number of C atoms of each metabolite is indicated inside circles

The map was constructed with KEGG Mapper (http://www.genome.jp/kegg/tool/map_pathway2.html) from the list of proteins from Table S8 and the carbon metabolism (bja01200) map as template. D-mannitol and D-fructose were added to the map by hand. The reactions catalyzed by Blr6758, Bll7540, Bll7541, Bll7543 (all in blue), and Bll3754 (in red) were colored by hand because, although those steps were annotated in the pentose-phosphate–pathway (bja00030) and the glyoxylate- and carboxylate-metabolism (bja00630) KEGG maps, the reactions were not annotated in the carbon-metabolism (bja01200) map.

Table S10. Stoichiometric balance of the predicted reactions for the catabolism of D-mannitol under the condition of 50% oxygenase and 50% carboxylase activities in the RuBisCO catalysis and with carbon recycling through the tetrahydrofolate (THF) and glyoxylate cycles. The enzymes in red belong to the MtlDP; the underlined enzymes were not observed in the Ara cultures. All the other enzymes were present in our samples, as detailed in Table S1.

**Substrates**	**Products**	**Enzymes detected (Rhizobase code)**
6 D-mannitol + 6 NAD^+	6 D-fructose + 6 NADH + 6 H^+	Blr3225
6 D-fructose + 6 ATP	6 D-fructose-6P + 6 ADP	Blr3227
12 D-fructose-6P	12 D-glucose-6P	Blr6758
12 D-glucose-6P + 12 NAD^+	12 D-glucono-1,5-lactone-6P + 12 NADH+ 12 H^+	Blr6760
12 D-glucono-1,5-lactone-6P + 12 H_2O	12 D-gluconate-6P	Blr0368
12 D-gluconate-6P + 12 NAD^+	12 D-ribulose-5P + 12 CO_2 + 12 NADH+ 12 H^+	Blr6759
2 D-ribulose-5P	2 D-ribose-5P	Blr3755
4 D-ribulose-5P	4 D-xylulose-5P	Blr2588
2 D-ribose-5P + 2 D-xylulose-5P	2 D-sedoheptulose-7P + 2 D-glyceraldehyde-3P	Bll2168, Blr2583
4 D-sedoheptulose-7P + 4 glyceraldehyde-3P	4 D-fructose-6P + 4 D-erythrose-4P	Blr6758
2 D-erythrose-4P + 2 D-xylulose-5P	2 D-fructose-6P + 2 D-glyceraldehyde-3P	Bll2168, Blr2583
2 D-glyceraldehyde-3P	2 glycerone-P	Bll4807
2 D-erythrose-4P + 2 glycerone-P	2 D-sedoheptulose-1,7P_2	Blr1521, Blr2584
2 D-sedoheptulose-1,7P_2 + 2 H_2O	2 D-sedoheptulose-7P + 2 Pi	Blr4363
6 D-ribulose-5P + 6 ATP	6 D-ribulose-1,5P_2 + 6 ADP	Blr2582
3 D-ribulose-1,5P_2 + 3 O_2	3 phosphoglycolate + 3 glycerate-3P	Blr2585, Blr2586
3 D-ribulose-1,5P_2+ 3 CO_2 + 3 H_2O	6 glycerate-3P	Blr2585, Blr2586
3 phosphoglycolate + 3 H_2O	3 glycolate + 3 Pi	Bll3754
3 glycolate + 3 O_2	3 glyoxylate + 3 H_2O_2	Bll7540, Bll7541, Bll7543
3 glyoxylate + 3 L-serine	3 hydroxypyruvate + 3 L-glycine	Bll6037, Blr0373
3 hydroxypyruvate + 3 NADH + 3 H^+	3 glycerate + 3 NAD^+	Non-annotated
3 glycerate + 3 ATP	3 glycerate-2P + 3 ADP	Blr7830
3 L-glycine + 3 5,10-methenyl-THF + 3 H_2O	3 L-serine + 3 THF	Bll5033, Bll5912
3 glycerate-3P + 3 NAD^+	3 3-phosphonooxypyruvate + 3 NADH + 3 H^+	Bll2918, Bll6549, Bll6814, Bll7401, Bll7965, Blr3173, Blr4558, Blr7063
3 3-Phosphonooxypyruvate + 3 L-glutamate	3 phospho-L-serine + 3 α-ketoglutarate	Bll7402
3 phospho-L-serine + 3 H_2O	3 L-serine + 3 Pi	Blr6505
3 L-glycine + 3 THF + 3 NAD^+	3 5,10-methenyl-THF + 3 NH_4^+ + 3 CO_2 + 3 NADH + 3 H^+	Bll0449, Bll4778, Blr5751, Blr5753, Blr6334
3 α-ketoglutarate + 3 NH_4^+ + 3 NADH + 3 H^+	3 L-glutamate + 3 NAD^+ + 3 H_2O	Blr7995
2 glycerate-3P + 2 ATP	2 glycerate-1,3P_2 + 2 ADP	Bll1522
2 glycerate-1,3P_2 + 2 NADH + 2 H^+	2 D-glyceraldehyde-3P + 2 Pi + 2 NAD^+	Bll1523
10 glycerate-2P	10 phosphoenolpyruvate + 10 H_2O	Bll4794
10 phosphoenolpyruvate + 10 ADP	10 pyruvate + 10 ATP	Blr7138
10 pyruvate + 10 CoA + 10 NAD^+	10 acetyl-CoA + 10 CO_2 + 10 NADH + 10 H^+	Bll0449, Bll4778, Bll4779, Bll4782, Bll4783, Blr2815, Blr4839

10 acetyl-CoA + 10 oxalacetate + 10 H_2O	10 citrate + 10 CoA	Bll0182, Blr4839
10 citrate	10 isocitrate	Bll0466
3 isocitrate	3 glyoxylate + 3 succinate	Blr2455
3 glyoxylate + 3 L-serine	3 hydroxypyruvate + 3 L-glycine	Blr0373, Bll6037
3 hydroxypyruvate + 3 NADH + 3 H^+	3 glycerate + 3 NAD^+	Non-annotated
3 glycerate + 3 ATP	3 glycerate-2P + 3 ADP	Blr7830
7 isocitrate + 7 NAD^+	7 α-ketoglutarate + 7 CO_2 + 7 NADH + 7 H^+	Blr5747
7 α-ketoglutarate + 7 CoA + 7 NAD^+	7 succinyl-CoA + 7 CO_2 + 7 NADH + 7 H^+	Bll0449, Bll0451, Bll0452, Bll4778, Blr6334
7 succinyl-CoA + 7 GDP + 7 Pi	7 succinate + 7 CoA + 7 GTP	Bll0455
10 succinate + 10 FAD	10 fumarate + 10 $FADH_2$	Blr0514, Blr0515
10 fumarate + 10 H_2O	10 malate	Bll0286, Bll5796, Blr6519
10 malate + 10 NAD^+	10 oxalacetate + 10 NADH + 10 H^+	Bll0456

Balance:

$$6\ C_6H_{14}O_6\ (Mtl) + 59\ NAD^+ + 10\ FAD + 10\ ATP + 7\ GDP + 33\ H_2O + 6\ O_2 \rightarrow$$
$$36\ CO_2 + 59\ NADH + 59\ H^+ + 10\ FADH_2 + 10\ ADP + 7\ GTP + 3\ Pi + 3\ H_2O_2$$

ATP yield:

[ATP + GTP]:	$-10 + 7$	=	-3
NADH:	59×3	=	177
$FADH_2$:	10×2	=	20
Total:			194
ATP/Mtl:			32.3
ATP/C-mole:			5.4

O_2 consumed:

From pathway			6
Rx 1: [$2\ NADH + 2\ H^+ + O_2 \rightarrow 2\ NAD^+ + 2\ H_2O$]:	59/2	=	29.5
Rx 2: [$2\ FADH_2 + O_2 \rightarrow 2\ FAD + 2\ H_2O$]:	10/2	=	5.0
Total:			40.5
O_2/Mtl:			6.7
O_2/C-mole:			1.1

Pyruvate produced (before TCA):

Total:	10
Pyruvate/Mtl:	1.7
Pyruvate/C-mole:	0.3

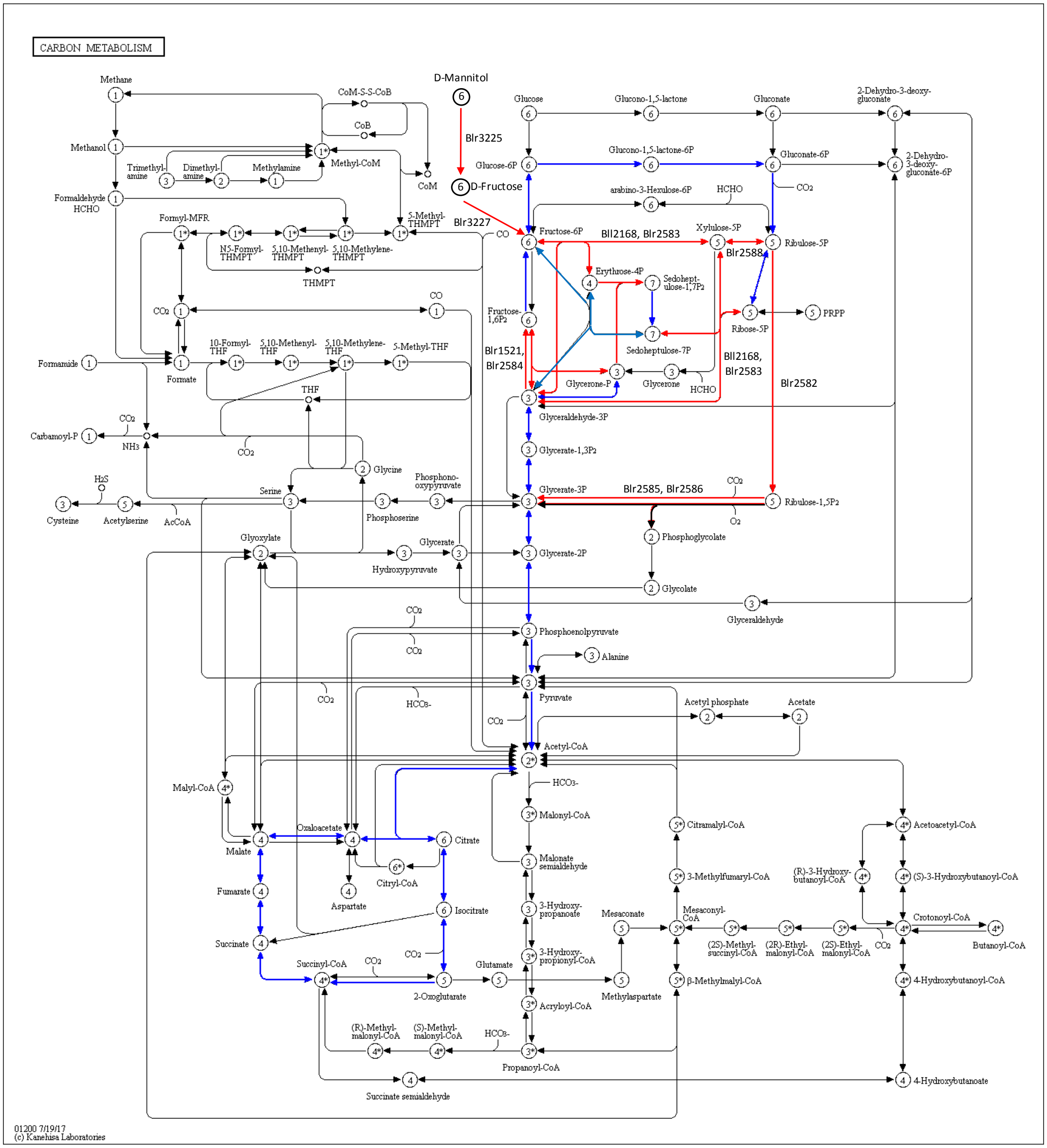

Fig. S7 Mtl catabolism through the CBB pathway with carboxylase as the sole activity in the RuBisCO catalysis. The reactions (indicated by their Rhizobase code) in red are catalyzed by the enzymes detected in the MtlDP; in blue, by the enzymes detected in the general pool, but not differentially expressed between Ara- and Mtl-grown cultures. The number of C atoms of each metabolite is indicated inside circles.

The map was constructed with KEGG Mapper (http://www.genome.jp/kegg/tool/map_pathway2.html) from the list of proteins from Table S11 and the carbon-metabolism (bja01200) map as a template. D-mannitol and D-fructose were added to the map by hand. The reaction catalyzed by Blr6758 (in blue) was colored by hand because, although that step was annotated in the pentose-phosphate–pathway KEGG map (bja00030), that reaction was not annotated in the carbon-metabolism (bja01200) map. The RuBisCO carboxylase activity has been colored in black to indicate the absence of that catalysis in this model.

Table S11. Stoichiometric balance of the predicted reactions for the catabolism of D-mannitol under the condition of 100% carboxylase activity in the RuBisCO catalysis. The enzymes in red belong to the MtlDP; the underlined enzymes were not observed in the Ara cultures. All the other enzymes were present in our samples, as detailed in Table S1.

Substrates	Products	Enzymes detected (Rhizobase code)
3 D-mannitol + 3 NAD^+	3 D-fructose + 3 NADH + 3 H^+	Blr3225
3 D-fructose + 3 ATP	3 D-fructose-6P + 3 ADP	<u>Blr3227</u>
6 D-fructose-6P	6 D-glucose-6P	Blr6758
6 D-glucose-6P + 6 NAD^+	6 D-glucono-1,5-lactone-6P + 6 NADH+ 6 H^+	Blr6760
6 D-glucono-1,5-lactone-6P + 6 H_2O	6 D-gluconate-6P	Blr0368
6 D-gluconate-6P + 6 NAD^+	6 D-ribulose-5P + 6 CO_2 + 6 NADH+ 6 H^+	Blr6759
1 D-ribulose-5P	1 D-ribose-5P	Blr3755
2 D-ribulose-5P	2 D-xylulose-5P	<u>Blr2588</u>
1 D-ribose-5P + 1 D-xylulose-5P	1 D-sedoheptulose-7P + 1 D-glyceraldehyde-3P	<u>Bll2168</u>, <u>Blr2583</u>
2 D-sedoheptulose-7P + 2 glyceraldehyde-3P	2 D-fructose-6P + 2 D-erythrose-4P	Blr6758
1 D-erythrose-4P + 1 D-xylulose-5P	1 D-fructose-6P + 1 D-glyceraldehyde-3P	<u>Bll2168</u>, <u>Blr2583</u>
1 D-glyceraldehyde-3P	1 glycerone-P	Bll4807
1 D-erythrose-4P + 1 glycerone-P	1 D-sedoheptulose-1,7P_2	Blr1521, <u>Blr2584</u>
1 D-sedoheptulose-1,7P_2 + 1 H_2O	1 D-sedoheptulose-7P + 1 Pi	Blr4363
3 D-ribulose-5P + 3 ATP	3 D-ribulose-1,5P_2 + 3 ADP	<u>Blr2582</u>
3 D-ribulose-1,5P_2+ 3 CO_2 + 3 H_2O	6 glycerate-3P	<u>Blr2585</u>, <u>Blr2586</u>
1 glycerate-3P + 1 ATP	1 glycerate-1,3P_2 + 1 ADP	Bll1522
1 glycerate-1,3P_2 + 1 NADH + 1 H^+	1 D-glyceraldehyde-3P + 1 Pi + 1 NAD^+	Bll1523
5 glycerate-3P	5 glycerate-2P	Blr2630
5 glycerate-2P	5 phosphoenolpyruvate + 5 H_2O	Bll4794
5 phosphoenolpyruvate + 5 ADP	5 pyruvate + 5 ATP	Blr7138
5 pyruvate + 5 CoA + 5 NAD^+	5 acetyl-CoA + 5 CO_2 + 5 NADH + 5 H^+	Bll0449, Bll4778, Bll4779, Bll4782, Bll4783, <u>Blr2815</u>, Blr4839
5 acetyl-CoA + 5 oxalacetate + 5 H_2O	5 citrate + 5 CoA	Bll0182, Blr4839
5 citrate	5 isocitrate	Bll0466
5 isocitrate + 5 NAD^+	5 α-ketoglutarate + 5 CO_2 + 5 NADH + 5 H^+	Blr5747
5 α-ketoglutarate + 5 CoA + 5 NAD^+	5 succinyl-CoA + 5 CO_2 + 5 NADH + 5 H^+	Bll0449, Bll0451, Bll0452, Bll4778, Blr6334
5 succinyl-CoA + 5 GDP + 5 Pi	5 succinate + 5 CoA + 5 GTP	Bll0455
5 succinate + 5 FAD	5 fumarate + 5 $FADH_2$	Blr0514, Blr0515
5 fumarate + 5 H_2O	5 malate	Bll0286, Bll5796, Blr6519
5 malate + 5 NAD^+	5 oxalacetate + 5 NADH + 5 H^+	Bll0456

Balance:

3 $C_6H_{14}O_6$ (Mtl) + 34 NAD^+ + 5 FAD + 2 ATP + 5 GDP + 3 Pi + 15 H_2O + $\rightarrow$
18 CO_2 + 34 NADH + 34 H^+ + 5 $FADH_2$ + 2 ADP + 5 GTP

ATP yield:

[ATP + GTP]:	$-2 + 5 =$	3
NADH:	$34 \times 3 =$	102
$FADH_2$:	$5 \times 2 =$	10
Total:		115
ATP/Mtl:		38.3
ATP/C-mole:		6.4

O_2 consumed:

From pathway:		0
Rx 1: [2 NADH + 2 H^+ + $O_2 \rightarrow$ 2 NAD^+ + 2 H_2O]:	34/2 =	17.0
Rx 2: [2 $FADH_2$ + $O_2 \rightarrow$ 2 FAD + 2 H_2O]:	5/2 =	2.5
Total:		19.5
O_2/Mtl:		6.5
O_2/C-mole:		1.1

Pyruvate produced (before TCA):

Total:	5
Pyruvate/Mtl:	1.7
Pyruvate/C-mole:	0.3

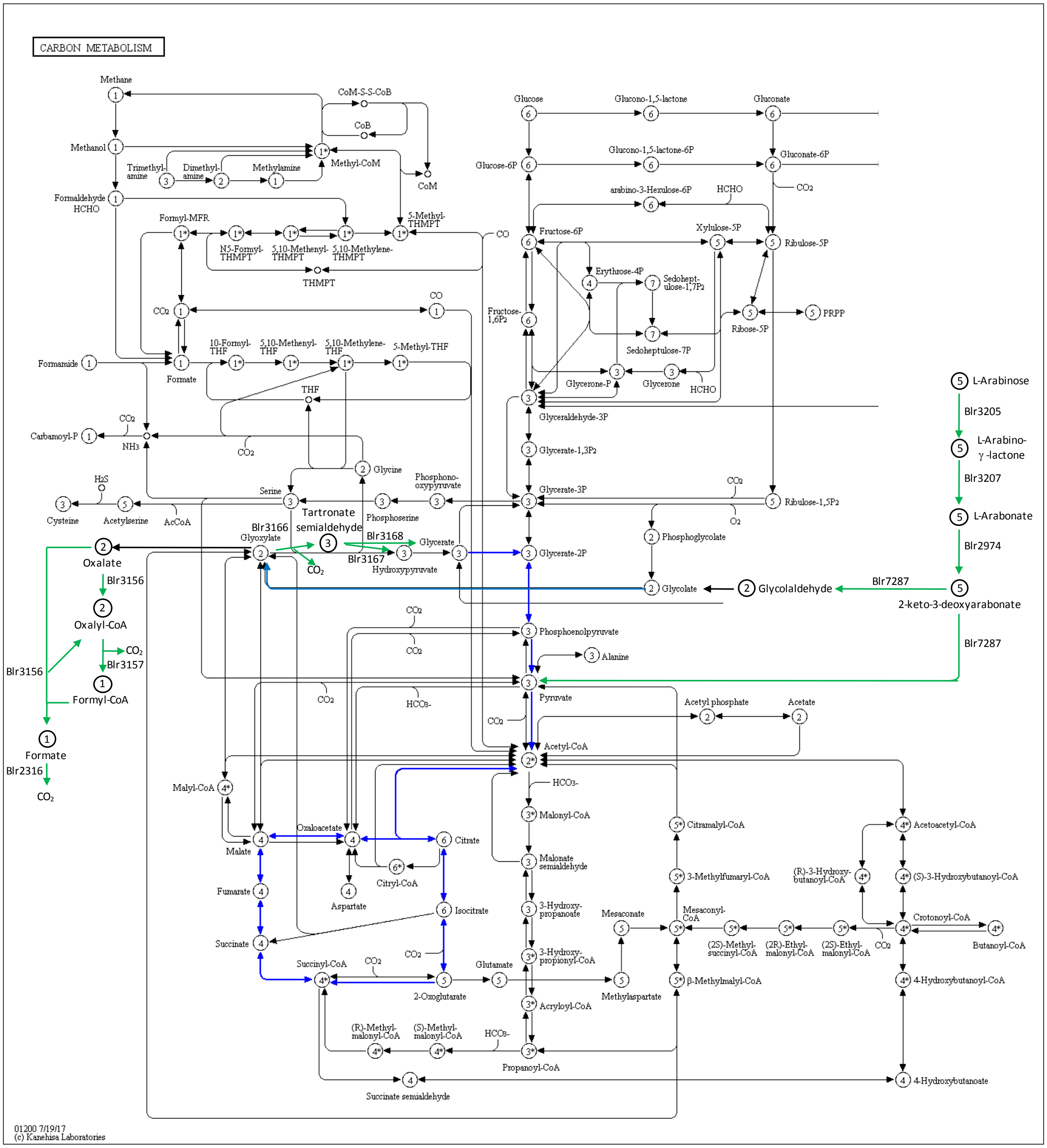

Fig. S8 Ara catabolism through the L-KDA pathway. The reactions (indicated by their Rhizobase code) in green are catalyzed by the enzymes detected in the AraDP; in blue, by the enzymes detected in the general pool, but not differentially expressed between Ara- and Mtl-grown cultures The number of C atoms of each metabolite is indicated inside circles.

The map was constructed with KEGG Mapper (http://www.genome.jp/kegg/tool/map_pathway2.html) from the list of proteins from Table S12 and the carbon-metabolism (bja01200) map as a template. The steps from Ara to glycolaldehyde and pyruvate and from glycolate to glycerate along with the oxalate-oxidation pathway were added to the map by hand. The reactions catalyzed by Bll7540, Bll7541, and/or Bll7543 (all in blue) were colored by hand because, although those steps were annotated in the glyoxylate- and carboxylate-metabolism KEGG map (bja00630), the reactions were not annotated in the carbon metabolism (bja01200) map.

Table S12. Stoichiometric balance of the predicted reactions for the catabolism of L-arabinose. The enzymes in green belong to the AraDP, while those in red belong to the MtlDP. Underlined enzymes belonging to the AraDP were not observed in the Mtl cultures. All the other enzymes were present in our samples, as detailed in Table S1.

Substrates	Products	Enzymes detected (Rhizobase code)
6 L-arabinose + 6 NAD^+	6 L-arabino-γ-lactone + 6 NADH + 6 H^+	Blr3205
6 L-arabino-γ-lactone + 6 H_2O	6 L-arabonate	Blr3207
6 L-arabonate	6 L-2-keto-3-deoxyarabonate + 6 H_2O	Blr2974
6 L-2-keto-3-deoxyarabonate	6 pyruvate + 6 glycolaldehyde	Blr7287
6 glycolaldehyde + 6 NAD^+ + 6 H_2O	6 glycolate + 6 NADH + 6 H^+	Non-annotated*.
6 glycolate + 6 O_2	6 glyoxylate + 6 H_2O_2	Bll7540, Bll7541, Bll7543, Blr4823
4 glyoxylate	2 tartronate semialdehyde + 2 CO_2	Blr3166
1 tartronate semialdehyde + NADH + H^+	1 D- glycerate	Blr3168
1 tartronate semialdehyde	1 hydroxypyruvate	Blr2929, Blr3167
1 hydroxypyruvate + NADH + H^+	1 D-glycerate + NAD^+	Bll0766, Blr7172
2 glyoxylate + 2 O_2 + 2 H_2O	2 oxalate + 2 H_2O_2	Non-annotated*
2 oxalate + 2 formyl-CoA	2 oxalyl-CoA + 2 formate	Bll3156
2 oxalyl-CoA	2 formyl-CoA + 2 CO_2	Bll3157
2 formate + 2 NAD^+	2 CO_2 + 2 NADH + 2 H^+	Blr2316, Blr2317
2 D-glycerate + 2 ATP	2 2-P-glycerate + 2 ADP	Blr7830
2 2-P-glycerate	2 phosphoenolpyruvate + 2 H_2O	Bll4794
2 phosphoenolpyruvate + 2 ADP	2 pyruvate + 2 ADP	Blr7138
8 pyruvate + 8 SH-CoA + 8 NAD^+	8 acetyl-CoA + 8 CO_2 + 8 NADH + 8H^+	Bll0449, Bll4778, Bll4779, Bll4782, Bll4783, Blr2815, Blr4839
8 acetyl-CoA + 8 oxalacetate + 8 H_2O	8 citrate + 8 SH-CoA	Bll0182, Blr4839
8 citrate	8 isocitrate	Bll0466
8 isocitrate + 8 NAD^+	8 α-ketoglutarate + 8 CO_2 + 8 NADH + 8 H^+	Blr5747
8 α-ketoglutarate + 8 SH-CoA + 8 NAD^+	8 succinyl-CoA + 8 CO_2 + 8 NADH + 8 H^+	Bll0449, Bll0451, Bll0452, Bll4778, Blr6334
8 succinyl-CoA + 8 GDP + 8 Pi	8 succinate + 8 SH-CoA + 8 GTP	Bll0455
8 succinate + 8 FAD	8 fumarate + 8 $FADH_2$	Blr0514, Blr0515
8 fumarate + 8 H_2O	8 malate	Bll0286, Bll5796, Blr6519
8 malate + 8 NAD^+	8 oxalacetate + 8 NADH + 8 H^+	Bll0456

*Non-annotated in Rhizobase, but its activity was experimentally determined (13).

Balance:

$$6\ C_5H_{10}O_5\ (Ara) + 44\ NAD^+ + 8\ FAD + 8\ GDP + 8\ Pi + 22\ H_2O + 8\ O_2 \rightarrow$$
$$30\ CO_2 + 44\ NADH + 44\ H^+ + 8\ FADH_2 + 8\ GTP + 8\ H_2O_2$$

ATP yield:

[ATP + GTP]:	0 + 8	=	8
NADH:	44×3	=	132
$FADH_2$:	8×2	=	16
Total:			156
ATP/Ara:			26.0
ATP/C-mole:			5.2

O_2 consumed:

From pathway			8
Rx 1: [$2\ NADH + 2\ H^+ + O_2 \rightarrow 2\ NAD^+ + 2\ H_2O$]:	44/2	=	22.0
Rx 2: [$2\ FADH_2 + O_2 \rightarrow 2\ FAD + 2\ H_2O$]:	8/2	=	4.0
Total:			34.0
O_2/Ara:			5.7
O_2/C-mole:			1.1

Pyruvate produced (before TCA):

Total:	8
Pyruvate/Ara:	1.3
Pyruvate/C-mole:	0.3
